# Structure-based design of CDC42 effector interaction inhibitors for the treatment of cancer

**DOI:** 10.1016/j.celrep.2022.110641

**Published:** 2022-04-05

**Authors:** Sohail Jahid, Jose A. Ortega, Linh M. Vuong, Isabella Maria Acquistapace, Stephanie J. Hachey, Jessica L. Flesher, Maria Antonietta La Serra, Nicoletta Brindani, Giuseppina La Sala, Jacopo Manigrasso, Jose M. Arencibia, Sine Mandrup Bertozzi, Maria Summa, Rosalia Bertorelli, Andrea Armirotti, Rongsheng Jin, Zheng Liu, Chi-Fen Chen, Robert Edwards, Christopher C.W. Hughes, Marco De Vivo, Anand K. Ganesan

**Affiliations:** 1 Department of Dermatology, University of California, Irvine, CA 92697, USA; 2 Laboratory of Molecular Modeling and Drug Design, Istituto Italiano di Tecnologia, Via Morego 30, 16163 Genoa, Italy; 3 Analytical Chemistry and Translational Pharmacology, Istituto Italiano di Tecnologia, Via Morego 30, 16163 Genoa, Italy; 4 Department of Biological Chemistry, University of California, Irvine, CA 92697, USA; 5 Department of Physiology and Biophysics, University of California, Irvine, CA 92697, USA; 6 Department of Pathology and Lab Medicine, University of California, Irvine, CA 92697, USA; 7 Department of Molecular Biology and Biochemistry, University of California, Irvine, CA 92697, USA; 8 These authors contributed equally; 9 Senior author; 10 Lead contact

## Abstract

CDC42 family GTPases (RHOJ, RHOQ, CDC42) are upregulated but rarely mutated in cancer and control both the ability of tumor cells to invade surrounding tissues and the ability of endothelial cells to vascularize tumors. Here, we use computer-aided drug design to discover a chemical entity (ARN22089) that has broad activity against a panel of cancer cell lines, inhibits S6 phosphorylation and MAPK activation, activates pro-inflammatory and apoptotic signaling, and blocks tumor growth and angiogenesis in 3D vascularized microtumor models (VMT) *in vitro*. Additionally, ARN22089 has a favorable pharmacokinetic profile and can inhibit the growth of BRAF mutant mouse melanomas and patient-derived xenografts *in vivo*. ARN22089 selectively blocks CDC42 effector interactions without affecting the binding between closely related GTPases and their downstream effectors. Taken together, we identify a class of therapeutic agents that influence tumor growth by modulating CDC42 signaling in both the tumor cell and its microenvironment.

## In brief

Jahid et al. report a multipronged approach to discover a drug candidate that can bind CDC42 family GTPases and can inhibit their interaction with downstream effectors. This molecule selectively targets CDC42 GTPases, inhibits MAPK and S6 signaling, and inhibits tumor growth and tumor angiogenesis.

## Graphical Abstract

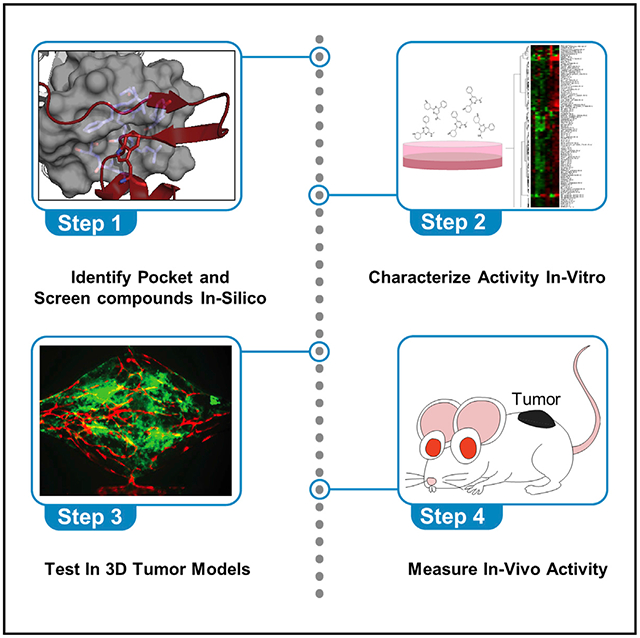

## INTRODUCTION

CDC42 family GTPases (RHOJ, RHOQ, CDC42) are linked to multiple human cancers and modulate cell-cycle progression, tumor cell migration/invasion, and tumor angiogenesis (Maldonado and Dharmawardhane, 2018). RHOJ, a known regulator of melanoma (Ruiz et al., 2017), breast cancer (Wang et al., 2017), and gastric cancer (Kim et al., 2016a) progression, activates signaling cascades in endothelial cells that are required for tumor angiogenesis (Wilson et al., 2014; Kim et al., 2014; Takase et al., 2012) and induces PAK signaling in tumor cells to promote growth (Ruiz et al., 2017). Similarly, CDC42 is a critical regulator of angiogenic sprouting (Boscher et al., 2019) and tubulogenesis (El Atat et al., 2019) in endothelial cells and promotes tumor cell proliferation and migration (Xiao et al., 2018). While RHOJ and CDC42 have not been found to be mutated in melanoma, they are often overexpressed (Tucci et al., 2007). CDC42 regulates melanoma invasion and is activated in BRAF-resistant melanoma cells (Gadea et al., 2008; Jobe et al., 2021; Lu et al., 2017). RHOJ regulates chemoresistance, invasion, and tumor angiogenesis in melanoma (Ho et al., 2012, 2013; Kim et al., 2014). In addition, CDC42 and its downstream p21-activated kinases (PAKs) are regulators of mitogen-activated protein kinase (MAPK) inhibitor resistance in BRAF mutant melanoma (Lu et al., 2017). Finally, RHOQ, while less studied, is also known to promote tumor invasion (Han et al., 2014) and angiogenesis (Bridges et al., 2020). Taken together, these observations suggest that CDC42 family GTPases are central players in several different processes critical for tumor progression/invasion, angiogenesis, and chemoresistance. Therefore, blocking signaling from CDC42 family members could have wide-ranging effects on tumor progression.

The GTPase activity of CDC42 family members is tightly regulated by (1) guanine nucleotide exchange factors (GEFs), (2) guanine nucleotide dissociation inhibitors (GDIs), and (3) GTPase-activating proteins (GAPs) (Haga and Ridley, 2016). CDC42 family member activity is also controlled by post-translational modification, including prenylation, which controls membrane localization (Haga and Ridley, 2016). CDC42 family members are considered “undruggable” due to their globular structure with limited small-molecule binding pockets and their high affinity for GTP/GDP (Maldonado and Dharmawardhane, 2018). Moreover, the tight regulation of CDC42 family member GTPases by GEFs, GAPs, and GDIs adds an additional level of complexity to targeting these proteins. Nonetheless, recent efforts have focused on developing small molecules that inhibit signaling from CDC42 family members and other closely related GTPases. Existing inhibitors block the ability of GEFs to activate CDC42 or the closely related GTPase RAC1 (Zins et al., 2013), prevent RAC/CDC42 from localizing to membranes (Pelish et al., 2006), or block RAC/CDC42 GTP binding (Hampsch et al., 2017). Unfortunately, the efficacy of these strategies is limited by the poor selectivity and/or poor bioavailability of the inhibitors (Maldonado and Dharmawardhane, 2018). Furthermore, as CDC42 and RAC1 have overlapping roles in platelet function (Pleines et al., 2013), targeting the signaling of both of these GTPases simultaneously often results in thrombocytopenia *in vivo* (Dutting et al., 2015). RAC1 also plays an important role in the cardiovascular system, and some CDC42/RAC1 inhibitors targeting RAC1 results in cardiotoxicity (Sawada et al., 2008, 2010; Tan et al., 2008). While newer inhibitors such as MBQ-167 can block the interaction of CDC42 and RAC1 with GEFs that activate them without inducing cardiotoxicity (Cruz-Collazo et al., 2021), these small molecules cannot target the CDC42 family GTPase RHOJ, which differs in its N-terminal sequence and is activated by a different mechanism (Ackermann et al., 2016; Florke et al., 2020). New structure-based, protein-protein interaction targeting strategies are needed to develop selective drug-like inhibitors that target all members of the CDC42 family, particularly given the important role of RHOJ in tumor angiogenesis.

Recent pioneering work has identified structural pockets in G12C mutant RAS and developed inhibitors that can covalently modify and inhibit G12C RAS function (O’bryan, 2019; De Vivo and Cavalli, 2017). While these approaches can be applied to RAS mutant cancers, they cannot be adapted to target CDC42 family GTPases as these proteins are rarely mutated. Here, we focus on CDC42 and RHOJ, two of the three CDC42 family members (CDC42, RHOJ, and RHOQ) that have the most defined roles in cancer. For clarity, we will refer to CDC42 family when describing effects that impact all of the family members (CDC42, RHOJ, RHOQ) and use the term CDC42 to describe effects only on CDC42 GTPase itself. Using a CDC42-PAK6 crystal structure (PDB: 2ODB), we generate an RHOJ-PAK1 structural homology model and identify a previously unappreciated binding pocket present only in GTP-bound CDC42 and RHOJ. We use structure-based virtual screening and molecular dynamics simulations (De Vivo et al., 2016) to identify compounds that putatively bind to the RHOJ/CDC42 effector interaction interface, measure the selective activity of these compounds against cancer cell lines and tumors, and demonstrate that they bind to CDC42 and inhibit CDC42 effector interactions.

## RESULTS

### Identification of a drug-binding pocket present in GTP-bound RHOJ and CDC42

**Figure 1.**
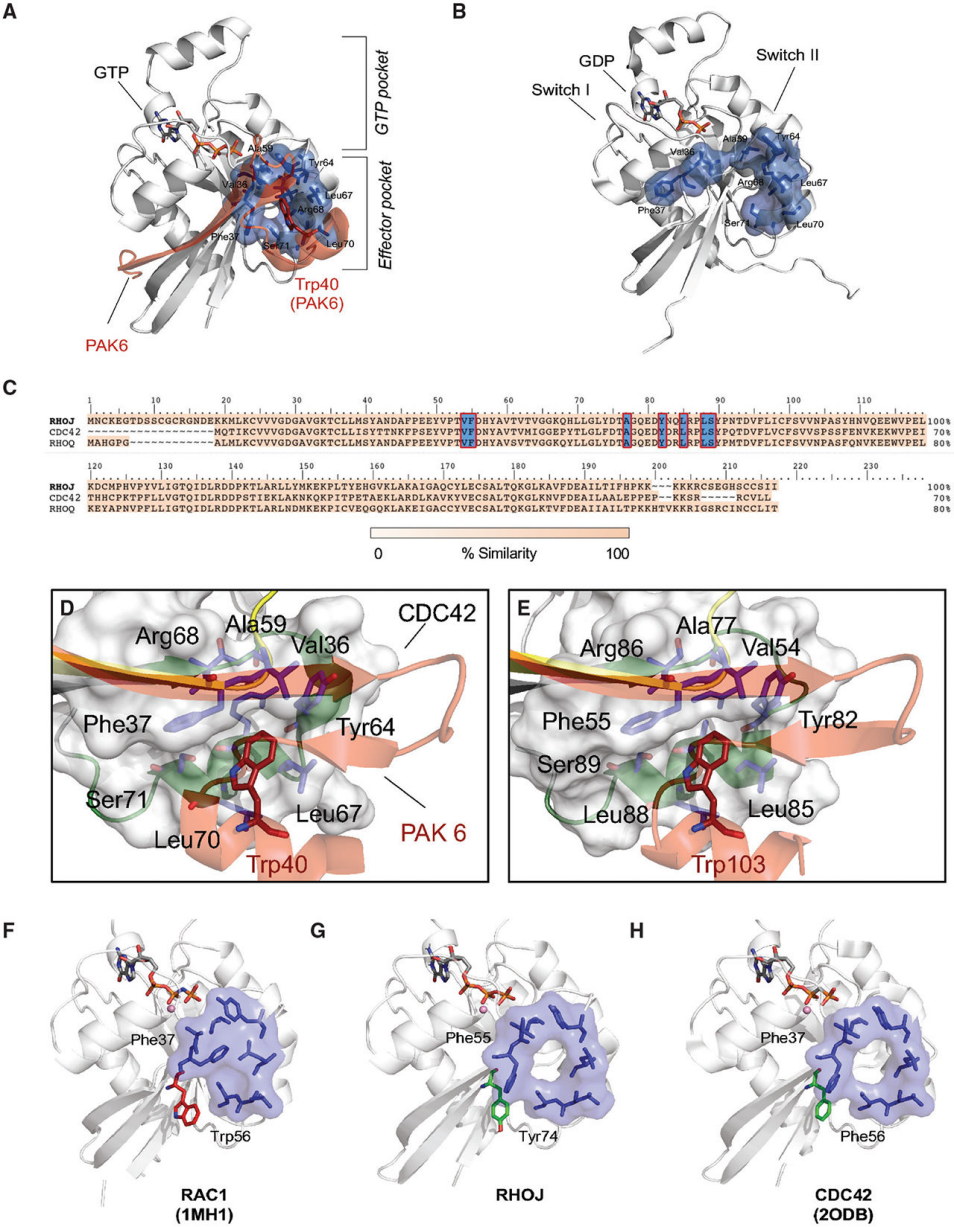
Structural identification of RHOJ/CDC42 allosteric effector binding pocket (A) Identification of a drug-binding pocket in GTP-bound CDC42. Structural modeling identified an effector pocket that is folded in the GTP-bound state (blue). CDC42 binds with PAK6 (transparent red) by Trp40 (red)-mediated interaction (PDB: 2ODB) in this putative drug-binding pocket. (B) Putative drug-binding pocket is not folded in the GDP-bound CDC42 structure. The structure of CDC42 is represented in GDP-bound (gray) state (PDB: 1DOA), where the effector pocket is unfolded (blue). (C) Key residues in the drug-binding pocket are conserved in CDC42-GTPases family. The alignments of CDC42, RHOJ, and RHOQ sequences are reported with overall and local sequence-similarity scores (white to pale yellow color scheme, 0%–100% local similarity). The residues defining the allosteric pocket are highlighted in blue. (D) Close view of the CDC42-PAK6 interaction interface. Interactions between Trp40 (red) of PAK6 (transparent red) and the effector binding pocket (light blue)of CDC42 (gray) are shown. Switch I and II regions are highlighted in yellow and green, respectively. (E) Close view of the RHOJ-PAK1 interaction interface. For comparison, modeling of the interaction between Trp103 (red) of PAK1 (transparent red) and the effector binding pocket (light blue) of RHOJ (gray) is shown. Switch I and II regions are again highlighted in yellow and green, respectively. The allosteric pocket is stable over 500-ns-long molecular dynamics simulations (Figures S1A and S1B). Adjacent residues may drive Rho-family specificity. (F) Structural representations of the “closed” conformation of Phe37 as captured in a RAC1 X-ray structure (PDB: 1MH1). (G and H) The open conformation of Phe37 and Phe55 found in CDC42 X-ray structures (H) and in the RHOJ homology model (G). The protein is represented in white cartoon, while guanine nucleotides and Mg^2+^ ions are sticks and balls, respectively. The drug-binding pocket is shown in stick and transparent surface (blue). The residues defining the drug-binding pocket are conserved in all the proteins, while the bulky Trp56 (red sticks) is present only in RAC1. In CDC42 and RHOJ, Trp56 is replaced by Phe56 and Tyr74, respectively (green sticks). Notably, the Trp56 may be responsible for the closed orientation of Phe37 in RAC1.

To identify putative drug-binding pockets in CDC42 family GTPases, we first examined the crystal structure of the CDC42 protein in both its GDP- and GTP-bound states. When comparing the crystal structures of GDP-bound CDC42 (PDB: 1DOA) (Hoffman et al., 2000) and GTP-bound CDC42 interacting with the CRIB domain of PAK6 (PDB: 2ODB), we discovered a previously unappreciated allosteric pocket at the CDC42-PAK6 protein-protein binding interface that is located on the CDC42 surface in proximity to Ser71, Arg68, and Tyr64 (Figures 1A and 1B). Specific residues of the structural motifs “switch I” (Val36, Phe37) and “switch II” (Ala59, Tyr64, Leu67, Leu70, Ser71) on the CDC42 surface define a pocket ~17 Å away from the GTP binding site, which accommodates the binding of Trp40 on PAK6 (Figure 1D). This pocket is conserved across structures of the CDC42 protein in complex with effectors such as PAK4 (PDB: 5UPK) (Ha and Boggon, 2018), PAK1 (PDB: 1EOA) (Morreale et al., 2000), and IQGAP (PDB: 5CJP) (Lecour et al., 2016) (a complete list and structural analyses are in Table S1; Figures S1A and S1B). This pocket is also found to be stable during 500-ns-long molecular dynamics (MD) simulations of GTP-bound CDC42 (root-mean-square deviation [RMSD]_pocket_ = 2.06 ± 0.33 Å; Figure S1C) and is the largest cavity at this protein-protein interface (Figures S1E and S1F) (La Sala et al., 2017). This distal pocket was unfolded in the CDC42-GDP complex (Figure 1B). CDC42 protein and all CDC42 family members activate PAK kinase. There are six PAKs that are grouped into two classes. Group I PAKs (PAK1–3) differ from group II PAKs (PAK4–6) secondary to the presence of an autoinhibitory domain. CDC42 itself has been shown to bind to group I PAKs and PAK4 (Ha and Boggon, 2018; Selamat et al., 2015; Baskaran et al., 2012), although the binding of other family members to PAK has been less well characterized.

Next, we sought to determine whether this same allosteric pocket could be used to target RHOJ effector interactions. As no crystal structure is available for RHOJ, we built a homology model for RHOJ to use as receptor for a virtual drug screening campaign. The alignment of RHOJ, CDC42, and RHOQ sequences revealed local sequence homology in the domains of RHOJ/RHOQ that interact with its downstream effector PAK, suggesting structural conservation of the pocket among all CDC42 family members (Figures 1C-1E and S1G). We verified that a similar allosteric binding pocket (involving the interaction of Trp103 of PAK1 with Ser89, Arg86, and Tyr82 of RHOJ) (Ho et al., 2012) was structurally conserved (Figure 1E) in RHOJ and stably maintained through additional 500-ns-long MD simulations of GTP-bound RHOJ. We observed that the overall structural fold of RHOJ was preserved during these simulations (RMSD_RHOJ_ = 1.20 ± 0.14 Å; Figure S1D), as it was the allosteric pocket (RMSD_pocket_ = 2.07 ± 0.27 Å; Figure S1D). This allosteric pocket is found always open in CDC42 and RHOJ, but not in RAC1, where Phe37 has been captured in a “closed” conformation that occludes the binding pocket (PDB: 1MH1) (Figures 1F-1H). This conformation is stably maintained during 500 ns of MD simulations (Figures S7H-S7J). Thus, this allosteric pocket could be targeted to specifically block RHOJ/CDC42 effector interactions and is structurally distinct from the CDC42 GEF interaction interface and the K-RAS domains targeted by others (Figures S2D-S2E) (Friesland et al., 2013).

### Identification of putative CDC42 family effector interaction inhibitors with anti-cancer activity

**Figure 2.**
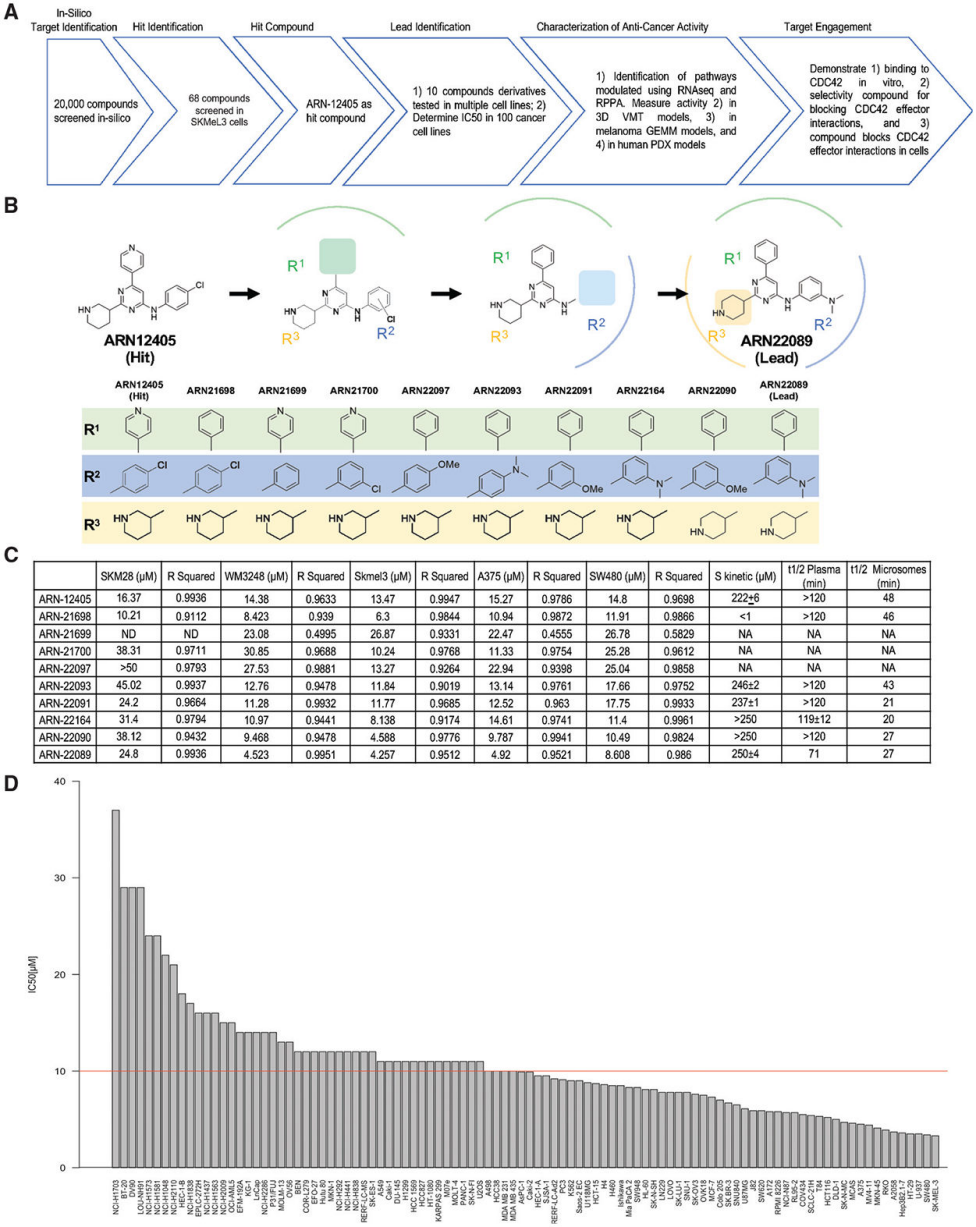
Structure-based screening identifies RHOJ/CDC42 allosteric inhibitors with broad-spectrum anti-cancer activity (A) Flow diagram of the screening steps to identify RHOJ/CDC42 allosteric inhibitors. (B) Chemical structures of the hit and synthesized derivatives. Names of the compounds and structures of the indicated R side chains are denoted below. (C) Table of IC_50_s, solubilities, and plasma and mouse microsomal half-life of lead and synthesized derivatives. Detailed IC_50_ curves are in Figure S2A. ND, no dose response for ARN220162 and ARN220163 (graphs in Figure S2F). (D) Bar graph shows IC_50_ values of ARN22089 in 100 cancer cell lines.

Next, we performed a virtual screening campaign of an internal chemical collection of compounds, which contains a diverse and non-redundant set of ~20,000 molecules, to identify RHOJ/CDC42 effector inhibitors. Initially, we selected 68 promising compounds and experimentally tested their half maximal inhibitory concentrations (IC_50_s) in SKMel28 melanoma cells—a cell line that we previously determined was sensitive to RHOJ depletion (Figure 2A) (Ho et al., 2012). We then selected 14 compounds with IC_50_s less than 50 μM (Table S2) for further analyses (54 screened compounds with IC_50_s greater than 50 μM or no IC_50_ at all are listed in Table S3). We first determined the compounds’ ability to inhibit the interaction between RHOJ or CDC42 and its downstream effector PAK using an established CDC42 interaction assay, which measures the interaction between RHOJ/CDC42 and the PAK-p21 binding domain (Figure S2B). We measured the kinetic solubility of the active compounds to evaluate their potential for further development. Our initial screening campaign identified ARN12405 as a promising hit (IC_50_ of 16.4 mM in the SKM28 cell line and a high kinetic solubility of 222 μM) that inhibits RHOJ/CDC42-PAK interactions (Figure S2B). Notably, ARN12405 features a functionalized pyrimidine scaffold bearing a 3-piperidine, a 4-chloro aniline, and a 4-pyridine, respectively, in the 2, 4, and 6 positions (Figure 2B). Modeling predicts that ARN12405 fits within the effector pocket of RHOJ and CDC42 (Figure S2C). To further assess the predicted binding poses from our docking results, we performed MD simulations of both RHOJ and CDC42 in complex with ARN12405. As shown in Figures S2D and S2E (left panels), our hit compound steadily binds the target pocket throughout the simulations (RMSD_ARN12405_ = 2.00 ± 0.40 and 3.24 ± 0.85 Å, for RHOJ- and CDC42-ligand complexes, respectively).

Next, we performed hit-to-lead optimization. We first generated new structural analogues and determined their IC_50_ in five different cancer cell lines (WM3248, SKMel3, A375, SW480, and SKM28), as well as kinetic solubility and half-life stability in mouse plasma and in mouse liver microsomes (Figures 2A-2C and S2A; synthesis details are in the STAR Methods). First, we simplified the starting scaffold to identify the more effective chemical features for RHOJ/CDC42 inhibition, replacing the pyridine heterocycle with a phenyl ring (ARN21698), which slightly increased the activity (IC_50_ 6.3–10.9 μM). Removal or moving the chlorine from the para to the meta position of the aniline moiety in position 4 (ARN21699, ARN21700), maintaining 4-pyridine, decreased the activity 2-fold compared with the hit in SKM28 cells. Next, we explored additional new aniline substituents while keeping the 3-piperidine heterocycle and the phenyl ring. The replacement of chlorine in the para position in ARN21698 by a methoxy (ARN22097) or a dimethyl amino group (ARN22093) resulted in a total or partial loss of activity. In contrast, introducing the same substituents (methoxy and dimethylamino groups) in the meta position (ARN22091 and ARN22164) moderately diminished the activity of the compounds. At this point, we explored different six-membered aliphatic heterocyclic compounds in position 2, moving first the piperidine nitrogen from position 3 to 4, keeping either a methoxy or a dimethyl amino substituent in the meta position (ARN22090 and ARN22089, respectively, which do not present a chiral center). These compounds are moderately active inhibitors in SKM28 cells (ARN22090 = IC_50_ 38.1 μM, ARN22089 = IC_50_ 24.8 μM). Notably, ARN22089 has a single-digit micromolar IC_50_ activity against the more sensitive cell lines that were tested (WM3248, SKMel3, A375, and SW480), an optimal kinetic and thermodynamic solubility (>250 and 268 μM, respectively), and a good half-life in mouse plasma (71 min) (Figures 2C and S6B). However, replacement of piperidine with a tetrahydropyran completely annihilated the activity as in ARN22162 and ARN22163 (Figure S2F, no dose response in SKM28 cell lines), suggesting that a basic free nitrogen center is critical for activity. In consideration of chemical tractability (synthesis and chirality), inhibitory activity, solubility, and half-life, ARN22089 was elected our lead compound. To examine whether ARN22089 had anti-cancer activity, we determined the IC_50_ of this compound in a panel of 100 cancer cell lines with various different mutations. Fifty-five of 100 cell lines, including several cell lines with neuroblastoma RAS (NRAS) mutations (Figure S2G), had an IC_50_ less than 10 μM Figures 2D and 2G; Table S4), demonstrating a broad spectrum of activity against cancer cells derived from many different tissues with different mutations.

### ARN22089 inhibits MAPK and S6 phosphorylation and influences NFkB signaling *in vitro*

**Figure 3.**
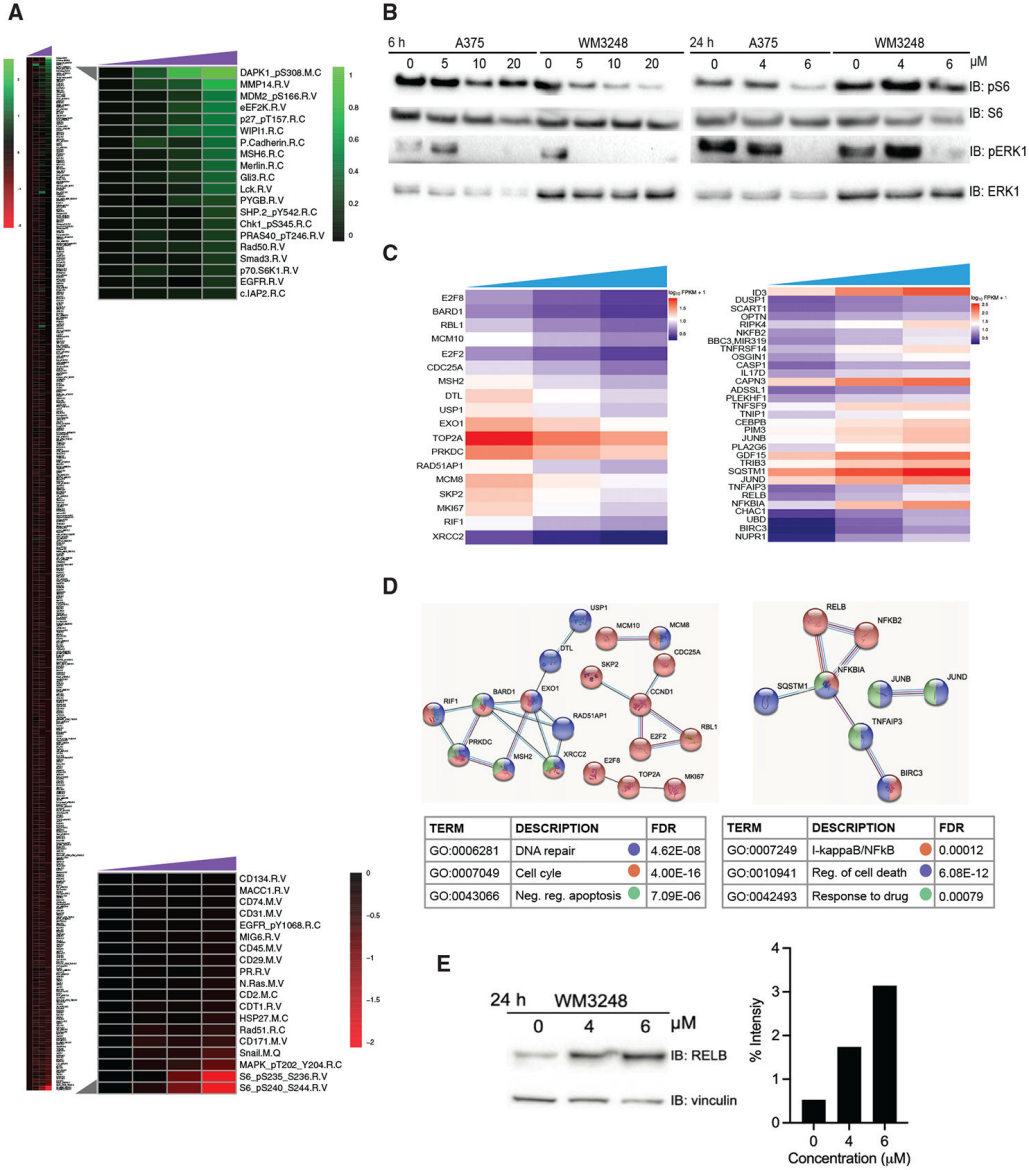
Characterization of the activity of ARN22089 in melanoma cells (A) RPPA heatmap showing effects of drug treatment on protein accumulation/phosphorylation. Colors represent values of each treated dose normalized to vehicle (0 μM) (Table S6). Left, heatmap shows results for 486 proteins assayed. Right, insets include proteins that were significantly modulated by drug treatment (Table S7). Purple triangles above the heatmap indicate increasing dosing (0, 5, 10, and 20 μM) for 6 h. (B) WM3248 or A375 cells were treated with the indicated doses of ARN22089, and the accumulation of pS6 and pERK (targets significantly modulated in the RPPA analysis) were measured by immunoblotting. (C) Profile heatmaps of mRNA sequences that were differentially transcribed after drug treatment (cutoff q value < 0.05, Table S8). Blue triangles above the heatmap indicate increasing dosing (0, 4, and 6 μM) for 24 h. (D) Protein-protein interaction networks modulated by drug treatment. Significant genes shown in (C) were subjected to STRING analysis to identify protein-protein interaction networks. Nodes are color-coded based on the Gene Ontology analysis shown in the table below. (E) Immunoblot for RELB from WM3248 cells that were treated with the indicated doses of ARN22089 for 24 h. ImageJ was used to quantify the bands; vinculin was used as a loading control. Bar graph represents percent intensity over increasing dosing.

Targeting protein-protein interaction interfaces has been notoriously difficult, secondary to the fact that these interaction interfaces are hydrophobic and span large contact areas (Lu et al., 2020). As a result, many designed protein-protein interaction (PPI) inhibitors fail early on in drug development, secondary to off-target activities. To understand the effect of ARN22089 on cancer cells, we first sought to examine how drug treatment influenced the activation of kinase signaling pathways. WM3248 melanoma cells were treated with 5, 10, or 20 μM ARN22089 for 6 h, cell lysates were prepared, and proteins were hybridized with a reverse-phase protein array consisting of 486 antibodies to quantitatively examine how the drug influenced protein abundance and protein phosphorylation. Remarkably, ARN22089 treatment significantly influenced the abundance of 38 proteins and the phosphorylation of only 10 proteins at both the 10 and/or 20 μM doses (Figures 3A, left, and S5A; Table S7). We observed that ARN22089 could inhibit S6 phosphorylation at both serine 235/236 and 240/244 residues with both 10 and 20 μM doses (Figure 3A, bottom inset). We also observed that ARN22089 could significantly inhibit phosphorylation at the 235/236 sites in both WM3248 and A375 melanoma cells with 10 and 20 μM doses at 6 h (Figure 3B) and with a dose of 5 μM at 24 h (Figure 3B). Another phosphorylation event that was inhibited by ARN22089 was MAPK1 (also known as ERK). We observed that ARN22089 inhibited ERK phosphorylation with 10 and 20 μM doses at 6 h (Figure 3B) and with a 5 μM dose at 24 h in A375 and WM3248 cells (Figure 3B). Although the reverse phase protein array (RPPA) analysis did include phospho-PAK antibodies in addition to phospho-ERK, as well as JNK and AKT, existing phospho-PAK antibodies have weak specificity, making changes difficult to detect on RPPAs dominated by more sensitive and selective antibodies. We did not observe an effect of ARN22089 on the abundance or phosphorylation of these proteins in our RPPA analysis (Figure S5B). Nonetheless, we did observe an inhibitory effect of ARN22089 on PAK phosphorylation in melanoma cells (Figure S5C). Taken together, these results indicate that ARN22089 is a selective inhibitor of PAK, ERK, and S6 phosphorylation *in vitro*.

Next, we sought to take a broader view of the pathways engaged by ARN22089. To approach this, we treated cells with either 0, 4, or 6 μM of ARN22089, harvested cells, and performed RNA sequencing to identify transcripts that were up- or downregulated upon ARN22089 treatment. We observed that ARN22089 treatment induced the expression of genes involved in cell death and nuclear factor κB (NF-κB) signaling, with resulting consequences on downstream RelB expression (Figures 3C and 3D; Table S8). We showed that the RELB protein level is increased with drug treatment compared with untreated cells, corroborating our RNA sequencing (RNA-seq) analysis (Figure 3E). GTP-bound CDC42 is known to bind to and activate p70S6K (Chou and Blenis, 1996). Active p70S6K goes on to phosphorylate the S6 ribosomal protein at two different locations, 234/235 and 240/244 (Ferrari et al., 1991). GTP-bound CDC42 family members can activate PAKs, whose downstream targets include members of the MAPK and NF-κB pathways (Basak et al., 2005). Taken together, the results presented here indicate that ARN22089 modulates two known CDC42 family effector functions: p70S6K activation and PAK activation.

Finally, we sought to examine whether ARN22089 engaged targets known to be an impediment to developing safe drugs. We verified that ARN22089 has no significant off-target effects as an agonist or an antagonist for a panel of 47 classical pharmacological targets, even at concentrations up to 25 μM (Figures S3A-S3E). Notably, ARN22089 does not target the hERG channel (Figure S3C), which in other cases has been an impediment in developing safe drugs (Kalyaanamoorthy and Barakat, 2018). Taken together, these results demonstrated that ARN22089 selectively inhibits two pathways known to be important in cancer progression, S6 phosphorylation, and MAPK activation.

### ARN22089 specifically inhibits tumor angiogenesis in 3D vascularized microtumors

**Figure 4.**
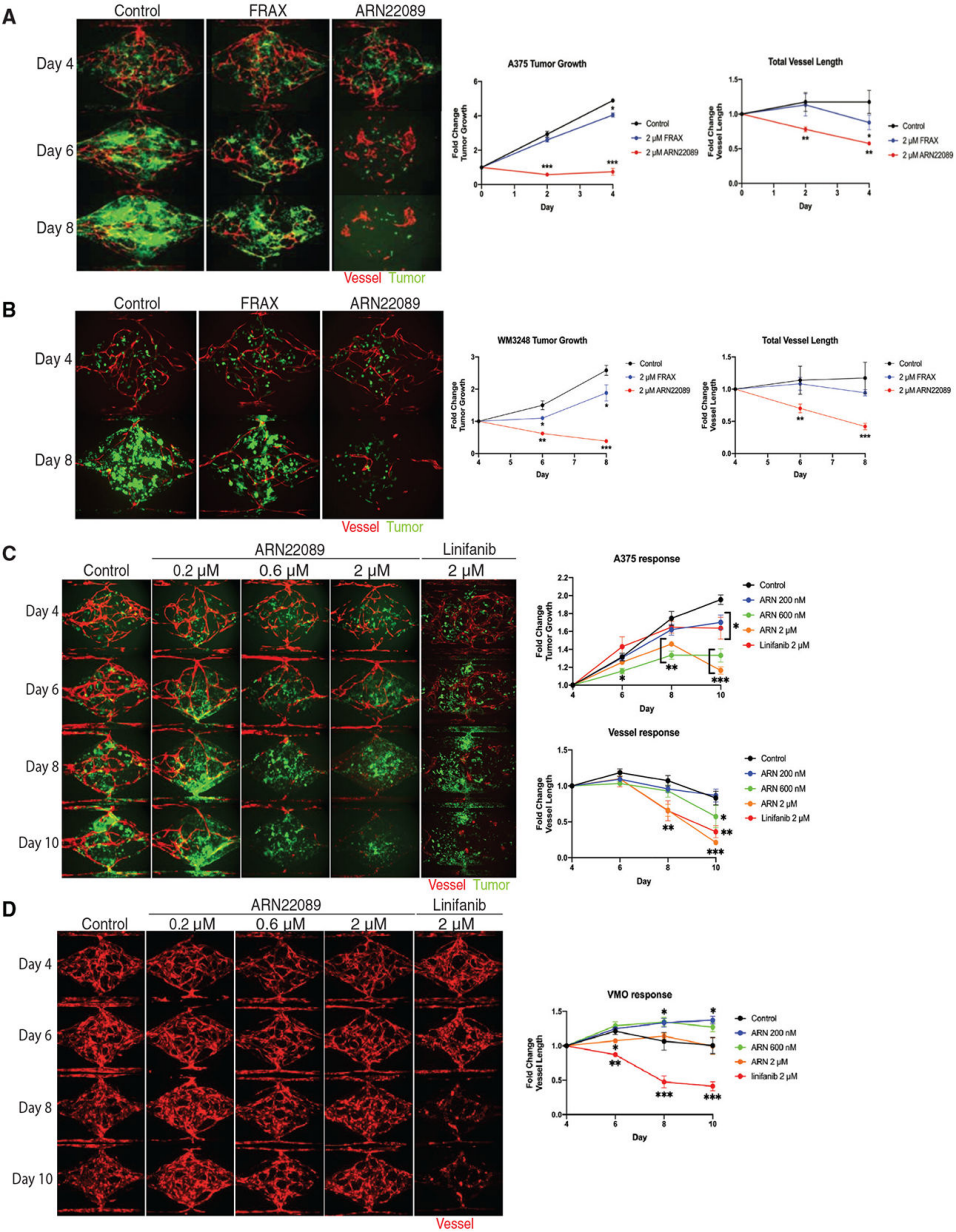
ARN22089 inhibits tumor growth in a vascularized microtumor (VMT) model (A and B) Representative micrographs of (A) A375 or (B) WM3248 VMTs (organoids that contain cancer cells and endothelial cells) treated with control, 2 μM FRAX, or ARN22089. Quantifications of tumor cell growth and vessel length are shown on right. (C) Micrographs of VMTs of WM3248 treated with increasing concentrations of ARN22089; corresponding quantification shown on right. The angiogenesis inhibitor linifanib at 2 μM is shown for comparison. (D) Micrographs of VMOs (organoids with only endothelial cells) treated with increasing concentrations of ARN22089 or linifanib with corresponding quantification of vessel length.

CDC42 and RHOJ are both known to have specific roles in tumor angiogenesis. To better understand how ARN22089 impacts tumor angiogenesis, we sought to test the ability of these compounds to inhibit vessel formation around tumors. Here, we utilized a vascularized microtumor platform (VMT)—a “tumor-on-a-chip” platform—that incorporates human melanoma cells, which are grown in a 3D extracellular matrix (ECM), and delivers nutrient to the cells via perfused micro-vessels (Hsu et al., 2013; Moya et al., 2013; Wang et al., 2016; Sobrino et al., 2016) (Figures S4A-S4C). Each chamber of the VMT was loaded with mCherry endothelial cells, GFP-labeled tumor cells (A375 and WM3248), fibroblasts, and pericytes. After 4 days, these cells formed a capillary bed that could feed the growing tumor, similar to newly formed vessels *in vivo* (Figure S4D). Nutrients and drug (ARN22089, FRAX597, vehicle) were perfused into the chamber from the high-pressure ”arterial side” and traversed to the tumor through the vascular network every 2 days at the indicated doses. The effects of each compound on the number of GFP tumor cells and the length of endothelial vessels was measured. Treatment of VMTs at a concentration of 2 μM ARN22089 inhibited the growth of both A375 cells and the mCherry-labeled blood vessels around the tumor (Figure 4A). In contrast, 2 μM FRAX597 did not significantly inhibit the growth of the A375 cells or the blood vessels (Figure 4A). These findings were confirmed in WM3248 cells, in which a dose of 2 μM ARN22089 caused significant tumor and vessel regression compared with both control and FRAX-treated VMT (Figure 4B). These observations indicate that ARN22089 inhibits both tumor growth and angiogenesis.

Next, we sought to more closely examine whether ARN22089 was a specific inhibitor of tumor angiogenesis. We generated VMTs that contained A375 cells and endothelial cells and vascularized micro-organs (VMOs) that contained vessels but no tumor cells. We observed that ARN22089 could inhibit the growth of both tumor cells and vessels in VMTs at 600 nM and 2 μM doses, which was significantly more effective than the angiogenesis inhibitor linifanib (Figure 4C). In contrast, ARN22089 did not inhibit the growth of VMOs (Figure 4D). We observed that linifanib, in contrast, did inhibit angiogenesis in VMOs (Figure 4D). These results suggest that ARN22089 specifically inhibits tumor, and not normal, angiogenesis, consistent with the selective role of CDC42 and RHOJ in tumor angiogenesis (Kim et al., 2014).

### ARN22089 has drug-like properties and inhibits tumor growth *in vivo*

**Figure 5.**
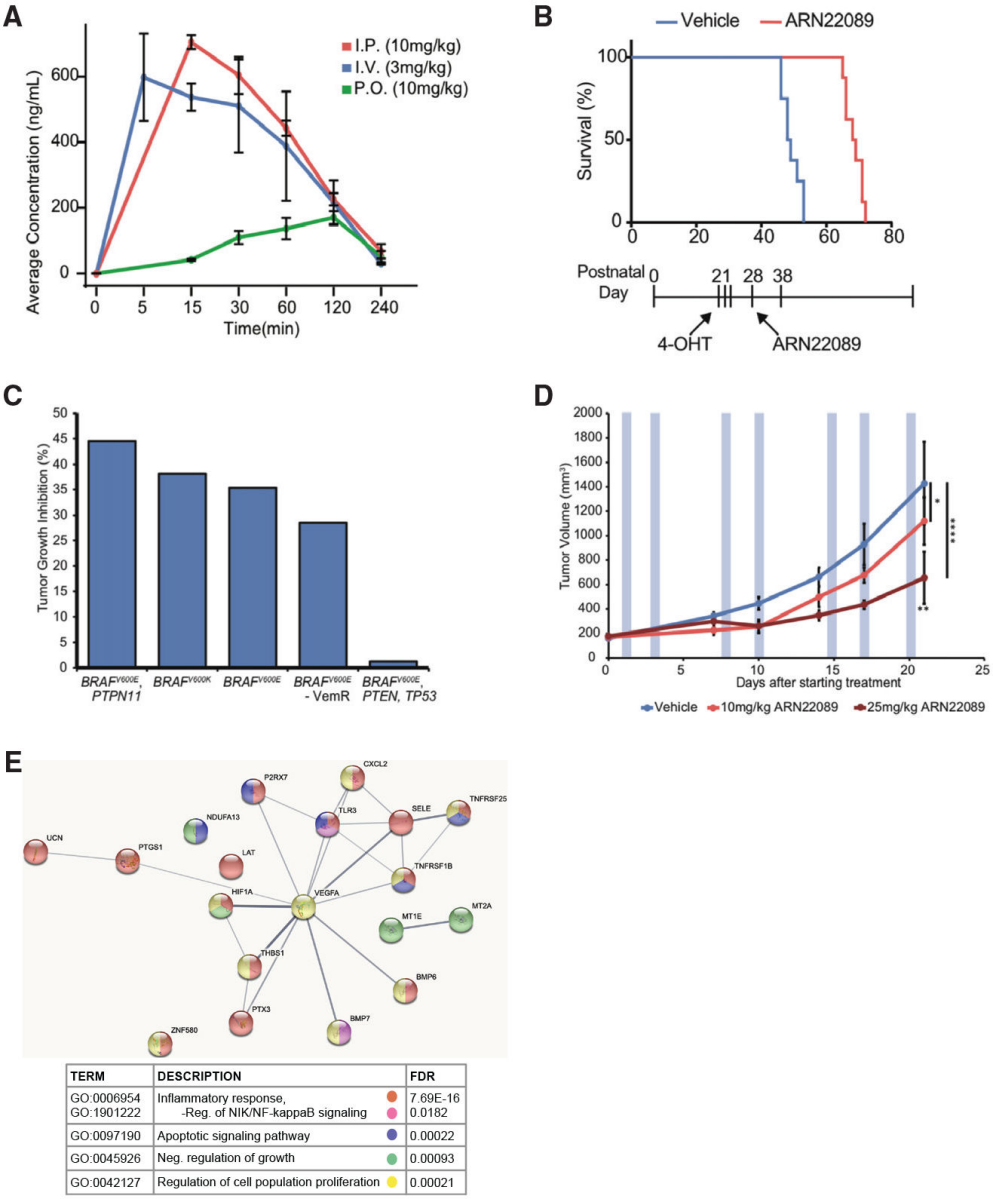
ARN22089 inhibits tumor growth in mouse models (A) ARN22089 delivered at 10 mg/kg i.p. and 3 mg/kg i.v. has drug-like properties. PK of compounds was calculated as described in the methods (complete data in Figure S6A). (B) ARN22089 inhibits melanoma development in a murine *BRAF^V600E^, PTEN^flox/flox^, Tyr:Cre^ERT2^* model. Melanoma was induced at P21–P23 with topical treatment of 25 mg/mL 4-OHT. Mice were then treated with vehicle (blue) or 10 mg/kg i.p. ARN22089 (red) for 10 days between P28–P38. Survival indicates time after tumor initiation to sacrifice when tumors in the mice become too large, moribund, or lost the ability to ambulate (n = 6 per group, p < .0001). (C) ARN22089 inhibits tumor growth in *BRAF* mutant PDX tumor models (listed by verified mutations). Mice were treated for 14 days with 10 mg/kg i.p. ARN22089 starting when PDX tumors reached 150–200 mm^3^. Tumor growth inhibition was compared with matched vehicle-treated mice (Figures S6C and S6D). (D) ARN22089 inhibits tumor growth in a dose-responsive manner. *BRAF^V600E^, PTPN11* PDX tumors (n = 6) were treated with vehicle (blue), 10 mg/kg ARN22089 (light red), or 25 mg/kg ARN22089 (dark red) twice weekly after tumors reached 150–200 mm^3^. Blue-shaded boxes denote i.v. treatments (Figure S6A). (E) RNA from the tumors described in (D) were sequenced. STRING was used to identify differentially expressed protein-protein interaction networks. Nodes are color-coded based on Gene Ontology indicated in the table below.

Once we determined that ARN22089 has selective effects on inhibiting cancer growth in 2D and 3D *in vitro* models, we next determined whether it has drug-like properties that would facilitate administration *in vivo*. We determined the pharmacokinetics of ARN22089 in experimental animals after intraperitoneal, intravenous, and oral administration (Figure 5A). The compound was well tolerated in experimental animals and had drug-like PK properties (Figures 5A and S6A). The low bioavailability observed in the oral administration is most likely related to the chemical structure of the compound and its pKa, which makes it positively charged in the acidic gastric environment. In the absence of transporters, the protonation makes the molecules much less prone to passive diffusion through endothelial cells. In addition, we incubated the compound with both human and rat liver microsomes and determined that the compound had a half-life of 107 min after incubation with rat microsomes and 510 min after incubation with human microsomes (Figure S6B).

To test the efficacy of the compound *in vivo*, we first induced BRAF mutant melanoma tumors with topical tamoxifen in *Tyrosinase:: CreERT2; Braf^CA/+^; Pten^fl/fl^;RhoJ^+/+^* animals at post-natal day 21 (P21), as described in our other publication (Ruiz et al., 2017), and treated the mice with ARN22089 for 10 days using a twice a day (BID) intraperitoneal (i.p.) dosing regimen. Inhibitor treatment prolonged the survival of tumor-carrying mice (mean survival: control 51.5, treated 68.5) (Figure 5B) after only 10 days of treatment. Once we had established that this compound could inhibit the growth of mouse tumors in immunocompetent mice, we next examined the ability of this compound to inhibit the growth of patient-derived xenografts (PDXs) in NOD scid gamma (NSG) mice. Animals were inoculated with PDX tumors, and tumor treatment was initiated after tumors reached a size of 150–200 mm^3^. Animals were treated with 10 mg/kg i.p. BID for 2 weeks, after which tumors were allowed to continue to grow until they reached endpoint (1,500–2,000 mm^3^). The compound significantly inhibited the growth of 2/5 PDX tumors tested, as measured by the percentage of tumor growth inhibition, and generally inhibited the growth of 4/5 of the tumors tested (Figures 5C and S6C). Interestingly, the PDX models tested included one PDX model that was derived from a patient who had failed both a BRAF inhibitor and immunotherapy, which also responded to the treatment (Figure S6D). Histologic evaluation of treated tumors revealed that ARN22089 treatment for 2 weeks increased the amount of tumor necrosis in three of the four tumors that responded to drugs (Figures S6E and S6F). To gauge the effects of long-term ARN22089 treatment on tumor growth, we inoculated mice with one BRAF mutant PDX that was known to have consistent growth properties and began treating the mice with ARN22089 with 0, 10, or 25 mg/kg intravenous (i.v.) twice a week (BIW) until control tumors reached endpoint. ARN22089 inhibited tumor growth in a dose-responsive manner without modulating weight of mice (Figures 5D and S6G). We harvested tumors treated at the 0 and 25 mg/kg i.v. doses and harvested RNA from tumors. RNA-seq analysis of tumors revealed that ARN22089 treatment modulated the expression of genes involved in NF-κB signaling in 25 mg/kg i.v.-treated tumors, as was observed in *in-vitro*-treated cells (Figure 5E; Table S9). Taken together, these results provide proof of principle that ARN22089 can inhibit tumor growth and vasculogenesis in vivo by modulating similar pathways that were observed *in vitro*.

### ARN22089 selectively binds and inhibits CDC42 effector interactions

**Figure 6.**
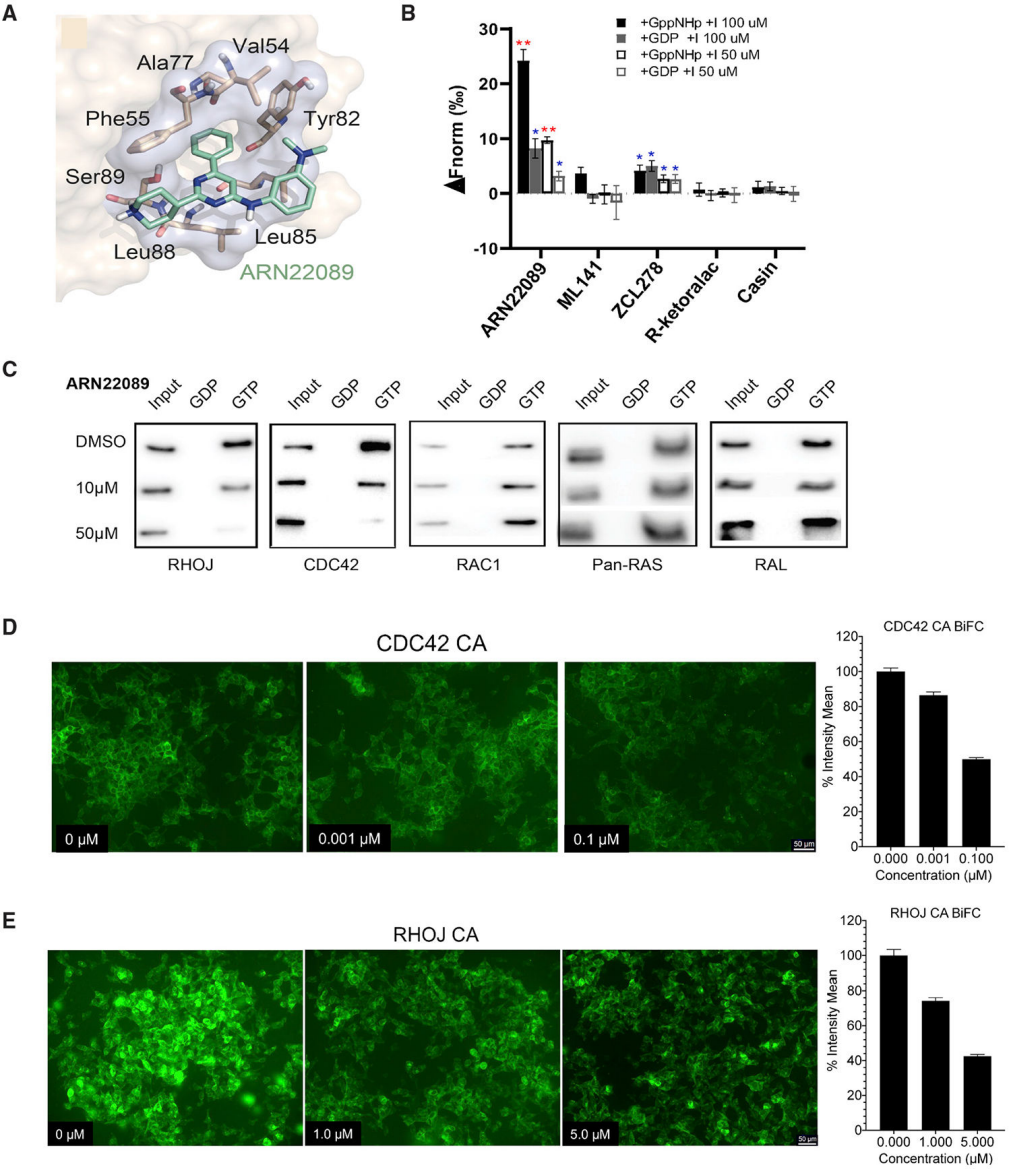
ARN22089 selectively blocks RHOJ/CDC42 effector interactions (A) Model structure of lead compound bound to the allosteric drug-binding pocket of RHOJ. (B) Microscale thermophoresis (MST) was used to screen for binding of the lead and known CDC42 inhibitors to a His-tagged CDC42 fragment. The graph displays the difference in normalized fluorescence (ΔF_norm_ [%] = ΔF_hot_/F_cold_) at 1.5–2.5 s between a protein:compound sample and a protein-only sample at the indicated drug concentrations after loading with the indicated nucleotides. A single asterisk highlights binding with a signal-to-noise ratio equal or higher than 5. Double asterisks are used for binding with a signal-to-noise ratio higher than 12. (C) ARN22089 selectively blocks CDC42 effector interactions. WM3248 cells were treated with ARN22089 for 24 h, and lysates were subjected to pull-down assays with PAK1 and RAF1 to measure the ability of the compound to block RHOJ, CDC42, RAC1, RAS, or RAL effector interactions. (D and E) Cells that expressed a constitutively active (CA) mutant form of the (D) CDC42 (Q61L) and (E) RHOJ (Q79L) and the wild-type (WT) form of the PAK1 were treated with 2 μg/mL of DOX to induce CDC42/RHOJ and PAK1 expression and treated with the indicated doses of ARN22089 for 24 and 8 h, respectively. Bar graphs (far right) show mean percentage of GFP intensity for each condition. The 0 μM condition was set to 100%. No DOX condition was used for background subtraction.

Modeling predicts that ARN22089 fits within the effector pocket of RHOJ and CDC42 (Figures 6A, S7A, and S7B). For both RHOJ- and CDC42-ARN22089 complexes, the binding pose is preserved during 500-ns-long MD simulations (RMSD_ARN22089_ = 3.55 ± 0.62 and 2.80 ± 0.61 Å, for RHOJ- and CDC42-ligand complexes, respectively; Figures S7C and S7D, right panels). As a further proof of principle, we examined the ability of the lead compound ARN22089 to bind to a purified CDC42 protein fragment. Initial studies by native mass spectrometry determined that purified CDC42 could be effectively loaded with GDP (90% efficiency of loading) or with the GTP analog GppNHp (>98% efficiency of loading; Figures S7E-S7G). Then, we observed by microscale thermophoresis that our lead ARN22089 binds better to the purified CDC42 compared with other known CDC42 inhibitors (ZCL278, ML141, R-ketoralac, and Casin) (Umbayev et al., 2018) (Figure 6B). Moreover, we found that ARN22089 binds CDC42 preferentially when the protein is in the GppNHp-loaded state, confirming our modeling results.

Next, we tested whether our compound was selective for blocking CDC42 family effector interactions in cells. To do this, we tested whether ARN22089 could inhibit the interactions between RHOJ or CDC42 and its downstream effector PAK without affecting the interaction between RAC1 and PAK using an established CDC42 effector assay (Kim et al., 2016b), which measures the binding between GTPases and their downstream effectors. In parallel, we performed a similar set of experiments to examine the ability of ARN22089 to inhibit the interaction between less closely related members of the RAS family and their downstream effectors (Rudolph et al., 2001). WM3248 cells were treated with the indicated doses of ARN22089, and cell lysates were prepared and incubated with EDTA to strip GTP and GDP, followed by incubation with GTP or GDP to load the GTPase. The samples were then incubated with PAK1-p21 binding domain (PAK1-PBD)-coupled beads or RAF1-RAS binding domain (RAF1-RBD)-coupled beads, which were then precipitated, and interacting proteins were identified by immunoblotting. ARN22089 inhibited the interaction between RHOJ or CDC42 and PAK1-PBD at 10 and 50 μM concentrations only when cell lysates were incubated with GTP (Figure 6C). No interactions were detected between RHOJ or CDC42 and PAK1-PBD when cell lysates were loaded with GDP (Figure 6C), consistent with previously published work (Ruiz et al., 2017). Notably, ARN22089 did not inhibit the interaction between the most closely related GTPase RAC1 and PAK1-PBD beads or between RAS or RAL and RAF1-RBD beads at either the 10 or 50 μM concentrations (Figure 6C), indicating that our lead compound was selective for CDC42 family members and does not inhibit RAC1, RAS, or RAL effector interactions. We also ran the assay at lower concentrations and observed that the compound inhibited CDC42-PAK interactions at concentrations that are consistent with the *in-vitro*-determined IC_50_ (Figure S8A). We also observed that the compound could inhibit the interaction prior to GTP loading (Figure S8A). Our hit compound (ARN12405) similarly blocked the RHOJ/CDC42 PAK interaction but not the RAC1 PAK interaction (Figure S2B). Taken together, these results indicate that ARN22089 selectively inhibits CDC42 family effector interactions without disrupting signaling from closely related GTPases (RAC1) that can sometimes be responsible for cardiotoxicity associated with existing inhibitors of this GTPase family (Sawada et al., 2010).

To further validate that ARN22089 inhibits CDC42 family effector interactions, we examined whether ARN22089 could inhibit CDC42/RHOJ-PAK interactions in cells. To measure CDC42 family effector interactions in cells, we used a bifluorescence complementation (BiFC) assay (Lepur and Vugrek, 2018), where CDC42 or RHOJ was linked to the N terminus of a Venus fluorescence protein (FP) fragment, while PAK1 is linked to a C-terminal Venus FP fragment (Figure S8B). We then measured CDC42/RHOJ PAK interactions by measuring Venus fluorescence in cells. Initial optimization studies revealed that we could visualize Venus indicative of CDC42 PAK interactions in cells that expressed constitutively active (CA) forms of RHOJ/CDC42 and PAK in the presence, but not the absence, of doxycycline (DOX), which induces the transcription of both proteins (Figure S8C). We next determined the percent intensity of CDC42-PAK interactions using different doses of ARN22089. We observed that ARN22089 could inhibit CDC42-PAK interactions, as measured by our BiFC assay with an estimated half maximal effective concentration (EC_50_) of 100 nM (Figures 6D and S8D), consistent with the observation that our hit compound bound to the CDC42 fragment with comparable affinities to compounds with nanomolar affinities (Figure 6B) (Maldonado and Dharmawardhane, 2018). We observed that ARN22089 could inhibit RHOJ/PAK interactions at an approximate EC_50_ between 1 and 5 μM (Figures 6E and S8E), consistent with the IC_50_s observed in the cancer cell lines tested.

## DISCUSSION

Targeting CDC42 family GTPases has been difficult secondary to its globular structure and limited small-molecule binding pockets (Maldonado and Dharmawardhane, 2018), its extensive post-translational modification in cells (Olson, 2018), and the fact that these proteins are localized to discrete nanodomains (Croise et al., 2014). We took a multipronged approach to identify molecules that would inhibit the PPI between CDC42 family members and their downstream effectors. We identify a structurally conserved, allosteric drug-binding pocket that is folded in GTP-bound CDC42 and RHOJ, is stable through 500-ns-long MD simulations, and is only partially formed and solvent exposed in GDP-bound CDC42 (Figure 1). This pocket directly interacts with Trp40 of PAK, indicating that molecules targeting this pocket would inhibit RHOJ/CDC42 and PAK interactions. Our modeling simulations revealed that RAC1 contains key structural differences in the drug-binding pocket that would make it unable to bind to ARN22089 (Figures S7H-S7J). As the drug-binding pocket occurs at the CDC42 PAK interface, it is conserved between RHOJ and CDC42, in contrast to the GEF interacting service, which is not conserved between these two family members (Figures 1F-1H). This allows us to selectively target CDC42 family members with ARN22089 without interfering with RAC1 signaling.

Starting from the discovery of the initial hit compound ARN12405, we built a structure-activity relationship (SAR) study that allowed us to develop the most promising new molecular entity, ARN22089, which we further characterized *in vitro*. We verified that (1) the lead (ARN22089) could bind to CDC42, (2) it bound preferentially to active CDC42, and (3) it bound more avidly than other known CDC42 inhibitors (Figure 6B). Importantly, in contrast to other CDC42 inhibitors, our drug selectively targeted the CDC42 family without targeting RAC1, which is known to activate PAK2 and induce cardiotoxicity in some cases (Moller et al., 2020). We did observe that many of the analogues in the SAR had similar anti-cancer activity, which is not surprising as the compounds target a large hydrophobic PPI interface as has been described for other PPI inhibitors (Lu et al., 2020). The identification of several compounds in our SAR that have similar structures but no activity, however, points to the specificity of our approach for identifying compounds that have a specific anti-cancer activity. To further verify target engagement, we examined whether ARN22089 has selective activity against CDC42 GTPases. ARN22089 could inhibit the interaction between RHOJ/CDC42 and PAK but did not block the interactions between the closely related RAC1 GTPase and PAK or RAS/RAL and Raf at doses as high as 50 μM (Figure 6C). As a further validation, we established a BiFC assay, which has been used to measure RHOJ PPIs (Lepur and Vugrek, 2018), to measure whether our compound could inhibit the PPI between RHOJ/CDC42 and PAK1. ARN22089 could inhibit the interaction between RHOJ/CDC42 and PAK1 with an EC_50_ in the nanomolar range for CDC42 and in the uni-digit micromolar range for RHOJ (Figures 6D and 6E). The data presented here are as extensive as data presented by others to validate the mechanism of action of CDC42 inhibitors (Liu et al., 2019). While co-crystallization, detailed mutagenesis studies, and further biochemical studies are planned for the future, the results presented here are nonetheless more than sufficient to establish that ARN22089 modulates CDC42 signaling.

Many groups have sought to develop small molecules that inhibit PPIs, including between Ras family members and their downstream effectors (Bery et al., 2018; Quevedo et al., 2018). While several of these agents have made it to the clinic, others failed secondary to their suboptimal selectivity and off-target effects (Murphy et al., 2021). ARN22089 had single-digit micromolar EC_50_s in a panel of cell lines (Figure 2D), consistent with the EC_50_ values observed with other RAC/CDC42 family inhibitors (Maldonado et al., 2020) and non-covalent RAS family PPI inhibitors (Bery et al., 2018; Quevedo et al., 2018). Using a RPPA, we observed that ARN22089 selectively modulated signaling pathways known to be downstream of CDC42 with little off-target effects on other kinase cascades when cells were treated with 10 or 20 μM of drug. ARN22089 significantly modulated the expression of only 38 targets and the phosphorylation of only 10 targets (Table S7 and Figure S5A). CDC42 can directly bind and activate p70S6K (Chou and Blenis, 1996), inducing the phosphorylation of S6K at 234/235. This specific phosphorylation event was inhibited by ARN22089 (Figure 3A). Moreover, we saw a more potent effect on this phosphorylation event when melanoma cell lines were incubated with this compound for longer periods of time (Figure 3B). PAK kinases can modulate ERK signaling by phosphorylating MEK at Ser298 (Wang et al., 2013) and also by phosphorylating Raf-1 (Coles and Shaw, 2002). We observed that ARN22089 could inhibit PAK and ERK phosphorylation (Figures S5C, 3A, and 3B). Taken together, these data indicate that while ARN22089 has a similar affinity for the PPI interface as other CDC42 inhibitors, it is highly selective, as we observed (1) no off-target activity as an agonist or an antagonist against a panel of 47 classical pharmacological targets (Figures S3A-S3E), and (2) a high degree of on-target modulation of CDC42 signaling (Figure 3).

Several other lines of evidence support the development of ARN22089 as a treatment for cancer. ARN22089 shows excellent solubility and has good chemical and metabolic stability, has a favorable absorption-distribution-metabolism-excretion (ADME) profile *in vitro*, and has favorable pharmacokinetics *in vivo* (intraperitoneral, intravenous, and oral administration). CDC42 family members regulate not only tumor cell proliferation but also tumor angiogenesis (Wilson et al., 2014; Kim et al., 2014; Haga and Ridley, 2016; Wang et al., 2016). ARN22089 inhibited not only tumor cell growth but also inhibited vessel elongation in 3D vascularized tumor models, in contrast to PAK inhibitors, which affect tumor proliferation but not vessel growth (Figures 4A-4C). Anti-angiogenic effects were specific for tumor-associated vessels, as ARN22089 had no impact on vessel growth when tumors were not present (Figure 4D). Finally, ARN22089 modulated the expression of genes involved in inflammation and apoptosis (Figures 3D and 5E), consistent with a role for the drug in inducing inflammation and apoptosis in tumors (Gan et al., 2016). Others have demonstrated that CDC42 and p70S6K directly interact and inhibit p70S6K’s ability to phosphorylate S6 (Chou and Blenis, 1996). Our RPPA analysis showed that ARN20089 modulates S6 phosphorylation, and S6 can also be activated by upstream signals that induce angiogenesis, such as vascular endothelial growth factor (VEGF) (Trinh et al., 2009). However, given the observations previously reported by others (Chou and Blenis, 1996), ARN22089 likely acts downstream of VEGF signaling by inhibiting S6 phosphorylation directly. Consistent with this, we did not observe a direct effect of ARN22089 on VEGF signaling in our RPPA analysis (Figure S5B). Of note, others have shown that CDKN2A deletion activates mTOR in melanoma tumors and induces S6 phosphorylation (Damsky et al., 2015). This model would be an ideal one to test the activity of our inhibitors and is planned for the future.

Before detailed formulation development, toxicokinetic, and pharmacokinetic characterization of the compound, we wanted to get a sense of the *in vivo* anti-cancer activity of the compound using a crude formulation. We demonstrated that short-term treatment of mice with ARN22089 could prolong survival in a BRAF mutant autochthonous mouse model of melanoma (Figure 5B). In addition, short-term treatment could slow the growth of 4/5 BRAF mutant human PDXs to some extent (Figure 5C), including one PDX model that was generated from a BRAF-inhibitor-resistant tumor (Figure S6D). Drug treatment induced necrosis in treated tumors, a phenomenon that is often observed with angiogenesis inhibitors (Yoshida et al., 2019), consistent with the data obtained in the VMT models (Figures S6E and S6F). Although we did not observe any overt toxicity in our mouse studies, these studies are still incomplete as we have not yet completed formulation-optimization and maximum-tolerated-dose studies with full *in vivo* toxicologic evaluation. This would be required to properly evaluate normal tissue toxicity of the drug. Notably, prolonged treatment of tumors with increasing doses of ARN22089 induced more profound tumor growth inhibition, an effect that was dose responsive (Figure 5D). Although further optimization of dosing is still needed, gene expression analysis of tumors revealed that ARN22089 modulates many of the same pathways *in vivo* that were observed *in vitro*. Given the unique spectrum of pathways modulated by ARN22089 and the effects of the drug on the tumor and its microenvironment, this therapy has logical applications both as a primary treatment, as a combination treatment to reduce the dose-limiting toxicities of existing agents, or as a treatment for tumors that have developed resistance to other agents. In a broader context, these studies provide a roadmap for the rational structure-based design of conformation-selective RHO superfamily GTPase inhibitors for other applications.

### Limitations of the study

The precise location of binding of ARN22089 to CDC42 was not determined through a crystal structure of the target/inhibitor complex. Disruption of the CDC42 and PAK1 interaction was deduced from the CDC42 activation and bifluorescence complementation assays as an *in vitro* system to recapitulate these interactions was unavailable. Limited drug-formulation studies were completed, and a maximum tolerated dose of ARN22089 in mice was not determined. As such, the proof-of-concept *in vivo* data presented here would benefit from further study.

## STAR★METHODS

### RESOURCE AVAILABILITY

#### Lead contact

Further information and requests for resources and reagents should be directed to and will be fulfilled by the lead contact, Anand K. Ganesan (aganesan@uci.edu)

#### Materials availability

All materials generated will be made available upon request after establishing relevant material transfer agreements.

#### Data and code availability

RNA-seq data have been deposited at GEO and are publicly available as of the date of publication. Accession numbers are listed in the key resources table. This paper does not report any original code. Any additional information required to reanalyze the data reported in this work is available from the lead contact upon request.

### EXPERIMENTAL MODEL AND SUBJECT DETAILS

#### Cell line

All cell lines were maintained according to the manufacturers. Details of cell culture and clone generations are described in Method details below.

#### Mice

All animal experiments were approved by the UC Irvine Institutional Animal Care and Use Committee (IACUC) (AUP-17-230). NOD.Cg-*Prkdc^scid^ IL2rg^tm1Wjl^*/SzJ (NSG) mice were purchased from The Jackson Laboratories (stock number 005557). *BRAF^V600E^, PTEN^flox/flox^, Tyr:Cre^ERT2^* mice were generated as described in Ruiz et al. (2017). Six to seven week mice, with equal number of males and females, were used in the experiments. Details of experimental procedure in mice are described in Method details below.

### METHOD DETAILS

#### Computations

##### Homology modeling

As a template structure, we employed the CDC42 protein in complex with the CRIB domain of Pak6 (PDB code PDB: 2ODB, resolution of 2.4 Å). By means of Prime (Jacobson et al., 2004) software implemented in Maestro, we modeled the FASTA sequence of RHOJ on the 2ODB X-ray structure (i.e., Cdc42 template), and then the resulting structure was refined by using the Protein Preparation Wizard (Sastry et al., 2013) workflow implemented in Maestro. According to this procedure, hydrogen atoms were added, and charges and protonation states were assigned titrating the protein at physiologic pH. The steric clashes were relieved by performing a small number of minimization steps, until the RMSD of the non-hydrogen atoms reached 0.30 Å.

##### Virtual screening

In the CDC42-PAK6 complex, we identified a pocket on the CDC42 surface in proximity of Ser71, Arg68 and Tyr64. Since this surface cavity is conserved in RHOJ, displaying the same residues as in CDC42 (i.e. Ser89, Arg86 and Tyr82), and also the tryptophan is conserved in PAK1, we used this pocket to center the grid. The cubic grid box was centered on Ser89 of RHOJ, having a dimension of 26 × 26 × 26 Å3. We screened a set of nonredundant ~20,000 molecules. These molecules belong to a proprietary chemical collection available in the D3 Department at IIT. The molecules database was prepared using LigPrep software implemented in Maestro. First, we added hydrogens and generated ionization states at pH 7.4 ± 0.5. We then generated tautomers and all stereochemical isomers. For each structure containing a ring moiety, the low-energy conformation was computed and retained. Last, a short minimization step was carried out to relax the 3D structure of each molecule. At this point, we filtered the resulting database to discard molecules that are not endowed with drug-like properties. To do so, we first computed the ADME descriptors for each molecule using QikProp software implemented in Maestro. As filter, we discarded all the molecules that do not respect the Lipinsky’s rule of five (Lipinski, 2004). We used Glide to perform the virtual screening, using Single Precision and retaining one pose for each ligand.

#### Molecular dynamics simulations

Molecular dynamics (MD) simulations were performed considering both our RhoJ structural model and Cdc42 X-Ray structure (PDB: 2ODB) in their ligand-free state, as well as four different protein-ligand complexes as obtained from our docking calculations. Specifically, two systems were built using our RhoJ structural model bound to either ARN22089 or ARN12405. Other two systems included Cdc42 (PDB: 2ODB) in complex with either ARN22089 or ARN12405. Additionally, the GTP substrate as well as the catalytic Mg^2+^ ion are present at the active site of the proteins, in all the systems. These models were hydrated with a 14Å layer of TIP3P water molecules (Jorgensen et al., 1983) from the protein center. The coordinates of the water molecules at the catalytic center were taken from PDB: 2ODB. Sodium ions were added to neutralize the charge of the systems. The final models are enclosed in a box of ~89·89·89 Å3, containing ~18,500 water molecules, resulting in ~59,000 atoms for each system.

The AMBER-ff14SB force field (Maier et al., 2015) was used for the parametrization of the protein. The parameters for the ligands ARN12405 and ARN22089 were determined via Hartree-Fock calculation, with 6-31G* basis set, convergence criterium SCF = Tight after structure optimization (DFT B3LYP functional; 6-31G* basis set). Merz-Singh-Kollman scheme (Singh and Kollman, 1984) was used for the atomic charge assignment. The GTP and the Mg^2+^ were parametrized according to Meagher KL et al. (Meagher et al., 2003) and Allner et al. (Allner et al., 2012), respectively. Joung-Cheatham parameters (Joung and Cheatham, 2008) were used for monovalent ions.

All MD simulations were performed with Amber 20 (Case et al., 2020) and all the systems were object of the following equilibration protocol. To relax the water molecule and the ions, we performed an energy minimization imposing a harmonic potential of 300 kcal/mol Å2 on the backbone, the GTP and the docked compound, when present. Then, two consecutive MD simulations in NVT and NPT ensembles (1 ns and 10 ns, respectively) were carried out, imposing the previous positional restraints. To relax the solute, two additional energy minimizations steps were performed imposing positional restraints of 20 kcal/mol Å2 and without any restraints, respectively. Such minimized systems were heated up to 303 K with four consecutive MD simulations in NVT (~0.1 ns, 100 K) and NPT ensembles (~0.1 ns, 100 K; ~0.1 ns, 200 K; ~0.2 ns, 303 K), imposing the previous positional restraints of 20 kcal/mol Å2. We used the Andersen-like temperature-coupling scheme (Andrea et al., 1983) while pressure control was achieved with Monte Carlo barostat at reference pressure of 1 atm. Long-range electrostatics were treated with particle mesh Ewald method. We performed an additional MD simulation (~1.5 ns) in the NPT ensemble at 303 K without any restraint to relax the system at such temperature. Finally, multiple replicas of 500 ns were performed in the NPT ensemble for each system with an integration time step of 2 fs.

#### Cancer cell line viability assay

Cancer cell line viability assay was performed using a CellTiterGlo assay. Cells were cultured in their appropriate media and seeded in white multi-well plates. For the assays, cells were incubated with compound at 37°C overnight, and a CellTiterGlo assay was performed to quantify viable cells. The IC_50_ was calculated in a panel of 100 cell lines.

#### Immunoblotting

Melanoma cells treated with ARN22089 for 6 or 24 h and lysed in RIPA buffer (EMD Millipore or an optimized cocktail (250 mM NaCl, 50 mM TrisCl pH 7.5, 0125% Nadeoxycholate, 0.375% Triton-X100, 0.15% NP-40, 4 mM EDTA) containing protease and phosphatase inhibitors cocktail (Thermo Scientific) and 1 mM PMSF and DTT. Lysates were then subjected to SDS-PAGE, transferred to PVDF membranes, followed by incubation with the indicated primary antibody and appropriate HRP-secondary antibody. ImageJ was used to perform densitometry.

A375 cells were maintained in DMEM (plus high glucose, L-glutamine, sodium pyruvate) supplemented with 10% FBS, 1% MEM NEAA and 1% antibiotic-antimycotic. WM3248 cells were maintained in 80% MCDB153 pH 7.30, 20% Leibovitz’s L-15, 2% FBS, 5 ug/mL insulin (bovine), 1.68 mM CaCl_2_, and 1% antibiotic-antimycotic. All cells were kept in 5% CO_2_ incubator at 37°C.

#### Reverse phase protein arrays (RPPA)

Melanoma cells (WM3248) were seeded at 3.6 × 10^6^ cells per 150 mm plate and, 24 h later, treated with ARN22089 at 0, 5, 10, or 20 μM. Six hours later, cells were scraped and centrifuged at 1.3 KRPM for 15 min cold (between 4-16°C range). Pellets were snap frozen in liquid nitrogen and shipped to MD Anderson Cancer Center core RPPA facility for RPPA analysis (Table S5). Three biological replicates for each sample were generated.

Multiple t test in Prism8 software was used to determine proteins with significant difference between 0 and 5, 10 or 20 μM. Normalized linear values were used to perform the t test with the following parameters: (1) Individual p values were computed with fewer assumptions by analyzing each row individually and did not assume consistent standard deviation; (2) Multiple comparisons were not corrected for; (3) alpha 0.05 was selected.

Heatmaps of RPPA represent normalized values (normalized linear values of 5, 10 or 20 μM divided by normalized linear values of 0 μM) (Tables S5-S7). Nomenclatures on the RPPA heatmaps: name_of_antibody.species_of_antibody.status_of_antibody. V - antibody validated; C - validation of antibody in progress; Q - antibody nonspecific; E – under evaluation; antibodies raised in M - mouse, G - goat, R - rabbit, or T - rat.

#### RNA-seq analysis

WM3248 melanoma cells were seeded at 8.4 × 10^4^ cells. The next day, cells were treated with ARN22089 at 0, 4, or 6 μM and harvested 24 h later by adding RLT lysis buffer (Qiagen). Similarly, a small chunk of PDX tumor was collected and lysed in RLT lysis buffer. RNA was extracted using a RNeasy kit (Qiagen) and sent to the Genomic Core Facility at UCI for library construction and sequencing.

For the cell lines, paired-end sequencing reads were aligned to the human reference genome (GRCh37/hg19) with Tophat v2.1 (used in conjunction with Bowtie2 v2.2.7 and Samtools v1.9) and processed with Tuxedo Suite (Cufflinks v2.2.1) (Trapnell et al., 2009, 2010). Heatmaps and other visualizations were generated using CummeRbund in R v4.0.3 (Goff et al., 2021).

For PDX tumors, paired-end sequencing reads were aligned to both human and mouse reference genome (NCBI/GRCh38 and UCSC/mm10, respectively) using HISAT2 v2.1.0 (Kim et al., 2019); and Samtools v0.1.19 to convert to bam files and sort. Then XenofilteR (Kluin et al., 2018) was used to remove mouse reads, and reads were counted using featureCounts (subread v1.5.0-p3) (Liao et al., 2014). Differential expression analysis was done using DESeq2 (Love et al., 2014) in R.

Additional analyses of gene expressions were performed using STRING (‘Search Tool for Retrieval of Interacting Genes/Proteins’) and DAVID (‘Database for Annotation, Visualization and Integrated Discovery’) to determine protein-protein network and functional annotation (Szklarczyk et al., 2019; Dennis et al., 2003).

#### Vascularized microtumor assay

Methods for microfluidic device fabrication and establishing VMTs have been described previously (Sobrino et al., 2016). Briefly, human endothelial colony-forming cell-derived endothelial cells (ECFC-EC) are isolated from cord blood via selection for the CD31 + cell population and cultured in EGM2 medium (Lonza). Normal human lung fibroblasts (LF) are purchased from Lonza (Hachey et al., 2021). Cancer cells and LF are cultured in DMEM (Corning) containing 10% FBS (Gemini Bio). The ECFC-EC and cancer cells were transduced with lentivirus expressing mCherry (LeGO-C2, plasmid # 27339) or green fluorescent protein (GFP) (LeGO-V2, plasmid # 27340) (Addgene, Cambridge, Massachusetts). To load the microfluidic device, ECFC-EC and LF (both 8 × 10^6^ cells/mL) and cancer cells (2.5 × 10^6^ cells/mL) were resuspended in fibrinogen solution (10 mg/mL basal medium). The cell slurry was then mixed with 1 mL thrombin (3 U/mL) to catalyze gel solidification and quickly loaded into the tissue chambers of each VMT unit. Fibrin ECM was allowed to solidify at 37°C for 15 minutes prior to introducing ECM and EGM2 medium through the microfluidic channels (Hachey et al., 2021). All cells and VMTs are cultured in a 37°C, 20% O_2_, 5% O_2_ environment.

#### Vascularized microtumor treatment

After culturing for 5 days to allow full development of each VMT, culture medium is replaced by medium containing the drugs (ARN22089, FRAX, Linifanib or vehicle) at the desired concentration and delivered through the microfluidic channels using the hydrostatic pressure gradient. Fluorescence images were acquired with an Olympus IX70 inverted microscope using SPOT software (SPOT Imaging, Sterling Heights, Michigan). AngioTool software (National Cancer Institute) (Zudaire et al., 2011) was used to quantify vessel length in the VMT and ImageJ software (National Institutes of Health) (Schneider et al., 2012) was utilized to measure the total fluorescence intensity (i.e. mean grey value) for each tumor image to quantify tumor growth. Each chamber was normalized to time 0 baseline.

#### PK determination

ARN22089 was administered at 10 mg/kg PO and IP, while it was injected at 3 mg/kg IV to male mice. The vehicle for all administration routes was PEG400/Tween 80/Saline solution at 10/10/80% in volume, respectively. Three animals per dose/time point were treated, and blood samples were collected at selected time points up to 480 min. Plasma was separated from blood by centrifugation for 15 min at 1500 rpm a 4°C, collected in eppendorf tubes and frozen (−80°C). Control animals treated with vehicle only were also included in the experimental protocol. Plasma samples were centrifuged at 21,100 g for 15 min at 4°C. A 50 μL aliquot was transferred into a 96-Deep Well plate and 150 μL of extraction solution was added. The extraction solution was consisting of cold CH_3_CN spiked with 200 nM of internal standard. The plate was centrifuged at 21.100 g for 15 min at 4°C. Eight microliters of supernatant were then transferred into a 96-Deep Well plate and 80μL of H_2_O was added. A reference standard of the compound was spiked in naïve mouse plasma to prepare a calibration curve over a 1 nM – 10 μM range. Three quality control samples were prepared by spiking the compound in blank mouse plasma to the final concentrations of 20,200 and 2000 nM. Calibrators and quality controls were extracted with the same extraction solution used for the plasma samples. The samples were analyzed on a Waters ACQUITY UPLC/MS TQD system (Waters Inc. Milford, USA) consisting of a TQD (Triple Quadrupole Detector) Mass Spectrometer equipped with an Electrospray Ionization interface and a Photodiode Array eλ Detector. The analyses were run on an ACQUITY UPLC BEH C18 (50 × 2.1 mmID, particle size 1.7 μm) with a VanGuard BEH C18 pre-column (5 × 2.1 mmID, particle size 1.7 μm) at 40°C. 0.1% HCOOH in H_2_O (A) and 0.1% HCOOH in CH_3_CN (B) were used as mobile phase with a linear gradient from 50 to 100% B in 2 min with the flow rate set to 0.5 mL/min. Electrospray ionization was applied in positive mode. Plasma levels of the parent compound was quantified by monitoring the MRM peak areas.

#### Aqueous kinetic solubility assay

The aqueous kinetic solubility was determined from a 10 mM DMSO stock solution of test compound in Phosphate Buffered Saline (PBS) at pH 7.4. The study was performed by incubation of an aliquot of 10 mM DMSO stock solution in PBS (pH 7.4) at a target concentration of 250 μM resulting in a final concentration of 2.5% DMSO. The incubation was carried out under shaking at 25°C for 24 h followed by centrifugation at 14.800 rpm for 30 min. The supernatant was analyzed by UPLC/MS for the quantification of dissolved compound (in μM) by UV at a specific wavelength (215 nm). The UPLC/MS analyses were performed on a Waters ACQUITY UPLC/MS system consisting of a Single Quadrupole Detector (SQD) Mass Spectrometer (MS) equipped with an Electrospray Ionization (ESI) interface and a Photodiode Array Detector (PDA). The PDA range was 210-400 nm. ESI in positive mode was used in the mass scan range 100-650 Da. The analyses were run on an ACQUITY UPLC BEH C18 column (50 x2.1 mmID, particle size 1.7 μm) with a VanGuard BEH C18 pre-column (5 × 2.1 mmID, particle size 1.7 μm), using 10 mM NH_4_OAc in H_2_O at pH 5 adjusted with AcOH (A) and 10 mM NH_4_OAc in CH_3_CN-H_2_O (95:5) at pH 5 (B) as mobile phase.

The thermodynamic solubility was determined by addition of Phosphate Buffered Saline (PBS) at pH 7.4 to an excess of solid test compound. The study was performed by incubation of an aliquot of 2.5 mg of test compound in 500 μL of PBS at pH 7.4 (final target concentration: 5 mg/mL). The suspension was shaken at 300 rpm for 24 h at 25°C. The pH of the suspension was measured at the beginning and at the end of the incubation. At the end of the incubation, the saturated solution was filtered and analyzed by LC-MS for the quantification of dissolved compound by UV at 254 nm using a calibration curve. The analyses were performed on a Waters ACQUITY UPLC-MS system consisting of a Single Quadrupole Detector (SQD) Mass Spectrometer equipped with an Electrospray Ionization interface and a Photodiode Array Detector from Waters Inc. (Milford, MA, USA). Electrospray ionization in positive mode was used in the mass scan range 100-650 Da. The PDA range was 210-400 nm. The analyses were run on an ACQUITY UPLC BEH C18 column (100 × 2.1mmID, particle size 1.7 μm) with a VanGuard BEH C18 pre-column (5 × 2.1mmID, particle size 1.7μm), using 10 mM NH_4_OAc in H_2_O at pH 5 adjusted with CH_3_COOH (A) and 10 mM NH_4_OAc in CH_3_CN-H_2_O (95:5) at pH 5 (B) as mobile phase.

#### *In vitro* mouse plasma stability assay

10 mM DMSO stock solution of test compound was diluted 50-fold with DMSO-H2O (1:1) and incubated at 37°C for 2 h with mouse plasma added 5% DMSO (pre-heated at 37°C for 10 min). The final concentration was 2 μM. At each time point (0, 5, 15, 30, 60, 120 min), 50 μL of incubation mixture was diluted with 200 μL cold CH_3_CN spiked with 200 nM of internal standard, followed by centrifugation at 3.750 rpm for 20 min. The supernatant was further diluted with H_2_O (1:1) for analysis. The concentration of test compound was quantified by LC/MS-MS on a Waters ACQUITY UPLC/MS TQD system consisting of a Triple Quadrupole Detector (TQD) Mass Spectrometer (MS) equipped with an Electrospray Ionization (ESI) interface. The analyses were run on an ACQUITY UPLC BEH C18 column (50 × 2.1 mmID, particle size 1.7 μm) with a VanGuard BEH C18 pre-column (5 × 2.1 mmID, particle size 1.7μm) at 40°C, using H_2_O + 0.1% HCOOH in H2O (A) and CH3CN + 0.1% HCOOH (B) as mobile phase. ESI was applied in positive mode. The response factors, calculated on the basis of the internal standard peak area, were plotted over time. Response factor versus time profiles were fitted with Prism (GraphPad Software, Inc., USA) to estimate compounds half-life (t½) in mouse plasma.

#### *In vitro* microsomal stability assay

10 mM DMSO stock solution of each compound was pre-incubated at 37°C for 15 min with liver microsomes from all the three species (mouse, rat, human) in 0.1M Tris-HCl buffer (pH 7.4). The final concentration was 4.6 μM. After pre-incubation, the co-factors (NADPH, G6P, G6PDH and MgCl_2_ pre-dissolved in 0.1M Tris-HCl) were added to the mixture and the incubation was continued at 37°C for 1 h. At each time point (0, 5, 15, 30, 60 min), 30 μL of mixture was diluted with 200 μL cold CH_3_CN spiked with 200 nM of internal standard, followed by centrifugation at 3500 g for 15 min. The supernatant was then further diluted with H_2_O (1:1) for analysis. The concentration of each compound was quantified by LC/MS-MS on a Waters ACQUITY UPLC/MS TQD system consisting of a TQD (Triple Quadrupole Detector) Mass Spectrometer equipped with an Electrospray Ionization interface. The analyses were run on an ACQUITY UPLC BEH C18 (50 × 2.1mm ID, particle size 1.7 mm) with a VanGuard BEH C18 pre-column (5 × 2.1mm ID, particle size 1.7 μm) at 40°C, using 0.1% HCOOH in H_2_O (A) and 0.1% HCOOH in CH_3_CN (B) as mobile phases. The percentage of test compound remaining at each time point relative to the amount observed at t = 0 was calculated. The half-lives (t½) were determined by a one-phase decay equation using a non-linear regression of compound concentration versus time.

#### Mouse strains and PDX tumors

All animal experiments were approved by the UC Irvine Institutional Animal Care and Use Committee (IACUC) (AUP-17-230). NOD.Cg-*Prkdc^scid^ IL2rg^tm1Wj^*/SzJ (NSG) mice were purchased from The Jackson Laboratories (stock number 005557). Human melanoma PDX tumors (563396-261-R, 128128-338-R, 174941-126-T, 156681-154-R, 425362-254-T) were purchased from Patient Derived Materials Repository (National Cancer Institute). Initial frozen tumors were divided into evenly sized pieces and kept viable in ice-cold RPMI (Corning) with 10% FBS. Small incisions were made on both flanks of anesthetized NSG mice, with a single tumor piece placed under the skin near each incision. Tumors were allowed to form and reach 10% body mass before passaging into subsequent mice for drug studies. Excess portions of the tumor were frozen in RPMI with 10% FBS and 10% DMSO. Following tumor placement, mice were monitored until the tumors reached 100-200 mm^3^ to start drug treatment.

#### Drug treatment for PDX tumors

ARN22089 was dissolved for 10 mg/kg doses in 100 μL of vehicle (80% Saline, 10% Tween80, and 10% PEG400). Stocks of concentrated drug were frozen at −20°C for long term and kept at 4°C for up to 5 days. Equal number of male and female NSG mice were used in the melanoma PDX experiment. Mice were treated with 100 μL of vehicle or RhoJ inhibitor twice daily for two weeks (morning and evening) through interperitoneal (IP) injections. Starting the same day as injections, tumor volumes were measured by caliper twice a week. Estimated tumor volumes were calculated by multiplying length by the width squared. At least four mice were imaged for each PDX during the course of treatment and continued until the tumors reached endpoint (1500 mm^3^). At endpoint, tumors were collected for histological analysis. For I.P. *BRAF^V600E^, PTPN11^N58S^* tumors; *BRAF^V600E^*– Vemurafenib Resistant tumors; *BRAF^V600E^, PTEN^H259Y^, TP53^C227Y^* tumors both male and female mice used in vehicle and ARN22089 treatment groups. Statistical analysis was completed on the tumor growth measurements using a two-way ANOVA comparing treatment groups for each PDX over time with TukeyHSD pairwise comparisons between timepoints in R (version 3.5.3).

Intravenous (IV) tail vein injections of ARN22089 were prepared at 10 mg/kg and 25 mg/kg as described above. Female mice were treated by IV twice a week as the *BRAF^V600E^, PTPN11^N58S^* tumors grew, for four weeks. Tumor and tissues were collected for histological analysis. Statistical analysis on tumor growth were completed in R (version 3.5.3) with a two-way ANOVA comparing treatment groups for each PDX over time with TukeyHSD pairwise comparisons between timepoints.

#### Tissue collection

Once tumors reached endpoint, various tissues were collected including the tumors, liver, and lymph nodes. All organs were fixed overnight in 10% formalin prior to washing and an ethanol dehydration series. Fixed tissue was sent to the Experimental Tissue Resource (UCI Department of Pathology) for embedding and preliminary staining by H&E and S100. The percentage of tumor necrosis was determined from H&E sections by a pathologist blinded to treatment groups. Statistical test on necrosis scores was completed using a student’s t-test in R (version 3.5.3).

#### His-Cdc42 production and purification

His-Cdc42 wild-type (amino acids Ile4-Pro182) was expressed in *E. coli* BL21 (DE3) cells using the pET28a expression vector. Overexpression was induced by 0.1 mM IPTG at OD_600_ 0.8 in Luria Bertani broth and incubated over night at 18°C. Cells were harvested by centrifugation at 6000 xg for 30 min and the pellet was stored at −80°C. Cells were defrosted by incubation at room temperature in 50 mM Tris-Cl buffer pH 7.5, 400 mM NaCl, 5 mM MgCl_2_, 50 μM GDP, 40 mM imidazole, 10 μg/mL DNAse (Sigma) and 30 μg/mL Lysozyme (Sigma). Cells were lysed by sonication and centrifuged at 43000 xg at 4°C for 1 h. The supernatant was incubated for 3h at 4°C, rotating, with Ni-NTA resin prior in batch purification. His-Cdc42 was eluted with 300 mM imidazole.

#### Preparation of GppNHP/GDP-bound GTPase

For the loading of GDP, the purified protein was dialyzed over night at 4°C in 50 mM Tris pH 8.5, 200 mM ammonium sulfate, 50 μM GDP (Jena Bioscience). For the loading of GppNHp, His-Cdc42 was instead dialyzed with 20 μM GppNHp (Jena Bioscience) in the presence of 5 U of Quick-CIP alkaline phosphatase (New England Biolabs). After dialysis, 2 mM MgCl_2_ was added to the solution to stabilize nucleotide binding. The two samples were buffer exchanged in 20 mM Hepes pH 7.5, 40 mM NaCl, 5 mM MgCl_2_, 1 mM DTT and loaded on a RESOURCE Q (Cytiva) column for anion exchange chromatography. The efficiency of nucleotide loading was evaluated by native state mass spectrometry (Vimer et al., 2020; Geoghegan et al., 1999). The purified proteins were buffer exchanged in 10 mM ammonium acetate pH 6.8 and diluted to 3 μM in 10 mM ammonium bicarbonate pH 6.5 added with final 3% acetonitrile. The samples were then infused at 40 μL/min in an electrospray ion source, coupled to a Synapt G2 QToF mass spectrometer operating in positive ion mode. Spectra were acquired over the 500-4000 m/z range.

#### Target binding by microscale thermophoresis

MicroScale Thermophoresis (MST) experiments were performed according to the NanoTemper technologies protocols in a Monolith NT.115 Pico (Pico Red / Nano Blue - NanoTemper Technologies). His-Cdc42 affinity for the RED-tris-NTA label was not optimal, therefore Alexa Fluor 647– NHS dye was used. His-Cdc42 was labeled following the instructions of the MO-L001 Monolith NT Protein Labeling Kit RED – NHS (NanoTemper Technologies). Labelled protein concentration in the binding reactions was 10 nM while compounds concentration was either 50 or 100 μM. DMSO concentration was maintained constant across samples at either 0.5% or 1%. Solutions were prepared in 100 mM Trizma® base (Sigma) pH 7.5, 40 mM NaCl, 0.05% v/v Tween 20 and incubated 5 min before loading on Premium Capillaries and analysis. Binding was detected at 24°C, MST power high and 20% LED power. The MST traces were recorded as follows: 3 s MST power off, 20 s MST power on and 1 s MST power off. The difference in normalised fluorescence (ΔF_norm_ [%] = F_hot_/F_cold_) between protein:compound sample and a protein only sample at 1.5-2-5 sec is calculated and plotted through MO.Affinity analysis v2.3 (NanoTemper Technologies) and GraphPad Prism 8.0.0 (GraphPad Software, San Diego, California USA). Signal to noise ratio was used to evaluate the quality of the binding data according to NanoPedia instructions (NanoTemper Technologies). Only a signal-to-noise ratio of more than 5 was considered acceptable while a signal to noise of more than 12 was considered excellent.

#### CDC42 interaction assay

A CDC42 activation assay was performed according to the manufacturer’s protocol (Cell Biolabs, San Diego, CA) as described previously (Ruiz et al., 2017). Briefly, cells expressing high RHOJ (WM3248 or WM983B) were treated with ARN22089 at the indicated doses. 24 hours later the lysates from treated and untreated cells were unloaded of guanosine nucleotides and either loaded with GDP or GTPγS. Agarose beads conjugated with the PAK1 PBD or RAF RBD were used in pull down assays for RHOJ, CDC42, RAC1 or RAS and RAL, respectively. Precipitated lysates were then immunoblotted with indicated antibodies.

#### Bi-fluorescence complementation assay

Inducible HEK293 Flp-In T-REx cell line was a gift from Dr. Jean-Francois Cote in the Montreal Clinical Research Institute (IRCM), Montreal, Quebec, Canada (Bagci et al., 2020). Cells were maintained in DMEM supplemented with 10% fetal bovine serum (TET tested, heat inactivated, R&D SYSTEMS INC), 1% antibiotic-antimycotic, 10 μg/mL blasticidin and 100 μg/mL zeocin at 37°C in 5% CO_2_. Stable BiFC lines were generated by co-transfecting pOG44 and pKK-BiFC-Venus-CDC42/RHOJ-PAK1 plasmids (9:1 ratio) and selected with 100 μg/mL hygromycin and 10 μg/mL blasticidin 24-48 h after transfection. The CDC42 and RHOJ genes in the pKK-BiFC-Venus-CDC42/RHOJ-PAK1 plasmid, the following residues were modified to generate constitutively active form: CDC42, Q61L; RHOJ, Q79L (Ziman et al., 1991; Vignal et al., 2000).

For CDC42 constitutively active (CA) assays, cells were seeded at 0.5 × 10^5^ cells per well of a 4-chamber slide. The next day, cells were treated with indicated doses and induced with 2 μg/mL doxycycline (DOX), simultaneously. Twenty-four hours later, cells were washed in PBS and fixed with 4% para-formaldehyde and 0.10% Triton-X100 in PBS for 15-20 min. Cells were then washed with PBS and incubated with blocking buffer (10% goat serum in PBS) for 30 min at RT and incubated with primary antibody (anti-GFP Invitrogen chicken Y fraction) at 1:1000 in blocking buffer 4 h RT, slow nutating. Cells were washed once with PBS buffer for 5 min. Secondary antibody (Alexa 488 nm) at 1:1000 in blocking buffer and cells were incubated for 1 h at RT, followed by DAPI 1:1000 in PBS for 20 min at RT. Cells were washed 3 times with PBS buffer, each time 5 min at RT. Slides were mounted with mounting medium (Vectorshield).

For RHOJ CA assays, cells were seeded at 1.5 × 10^5^ cells per well of a 4-chamber slide. Next day, cells were treated with indicated doses and induced with 2 μg/mL doxycycline (DOX), simultaneously. Eight hours later, cells were washed in PBS and fixed with 4% para-formaldehyde and 0.10% Triton-X100 in PBS for 15-20 min. Subsequent steps both clones were processed the same. Cells were then washed with PBS and incubated with blocking buffer (10% goat serum in 1 x PBS) for 30 min at RT and incubated with primary antibody (anti-GFP Invitrogen chicken Y fraction) at 1:2000 in blocking buffer overnight at 4°C, slow nutating. Cells were washed once with PBS buffer for 5 min. Secondary antibody (Alexa 488 nm) at 1:1000 in blocking buffer and cells were incubated for 1 h at RT, followed by DAPI 1:1000 in PBS for 20 min at RT. Cells were washed 3 times with PBS buffer, each time 5 min at RT. Slides were mounted with mounting medium (Vectorshield).

Slides were viewed using the Keyence BZ-X810 Wide-Field Microscope in the Stem Cell Research Center at the University of California, Irvine (UCI). Images were captured at high resolution with the same exposure time. Fluorescent images were quantified using Imaris software (BitPlane). Measurement setting includes surface masking [parameters: segment only a region of interest (excluding bright and blurry spots), classify surfaces, object-object statistics] to measure absolute intensity (threshold intensity adjusted for each image), and filter was set to “Number of Voxels Img = 1” > 10. Intensity mean of all areas average from 5-6 images per condition at 20× magnification. Percent average intensity mean was calculated as follow: ((Dose_1-NoDOX)/(DOX-NoDOX)) x 100). The standard error of mean was calculated taking the percent mean of intensity mean and divided by the square root of the number of surface areas measured.

#### Chemical synthesis and information

##### Chemistry: General experimental

###### Chemistry general considerations.

All the commercial available reagents and solvents were used as purchased from vendors without further purification. Dry solvents were purchased from Sigma-Aldrich. Automated column chromatography purifications were done using a Teledyne ISCO apparatus (CombiFlash® Rf) with pre-packed silica gel columns of different sizes (from 4 g up to 24 g) and mixtures of increasing polarity of cyclohexane and ethyl acetate (EtOAc) or dichloromethane (DCM) and methanol (MeOH). NMR experiments were run on a Bruker Avance III 400 system (400.13 MHz for ^1^H, and 100.62 MHz for ^13^C), equipped with a BBI probe and Z-gradients. Spectra were acquired at 300 K, using deuterated dimethylsulfoxide (DMSO–*d_6_*) or deuterated chloroform (CDCl_3_) as solvents. For ^1^H NMR, data are reported as follows: chemical shift, multiplicity (s= singlet, d= doublet, dd= double of doublets, t= triplet, q= quartet, p= pentuplet, m= multiplet), coupling constants (Hz) and integration. UPLC/MS analyses were run on a Waters ACQUITY UPLC/MS system consisting of a SQD (Single Quadrupole Detector) Mass Spectrometer equipped with an Electrospray Ionization interface and a Photodiode Array Detector. PDA range was 210-400 nm. The analyses were performed on an ACQUITY UPLC BEH C18 (50 × 2.1 mmID, particle size 1.7μm) with a VanGuard BEH C18 pre-column (5 × 2.1 mmID, particle size 1.7 μm) (LogD>1). The mobile phase was 10mM NH_4_OAc in H_2_O at pH 5 adjusted with AcOH (A) and 10mM NH_4_OAc in CH_3_CN-H_2_O (95:5) at pH 5 (B). Electrospray ionization in positive and negative mode was applied in the mass scan range 100-500 Da. Depending on the analysis method used, a different gradient increasing the proportion of mobile phase B was applied. For analysis method A, the mobile-phase B proportion increased from 5% to 95% in 3 min. For analysis method B, the mobile-phase B proportion increased from 50% to 100% in 3 min. All final compounds displayed ≥93% purity as determined by UPLC/MS analysis. Compounds ARN22097, ARN22091, ARN22093, ARN22089, ARN22090, ARN22164 were synthesized in house, compounds ARN12405, ARN21698, ARN21700, 21699 were purchased from Sigma-Aldrich and Asinex.

##### General scheme of total synthesis of compounds ARN22097, ARN22093, ARN22091, ARN22164, ARN22090, ARN22089, ARN22162, ARN22163


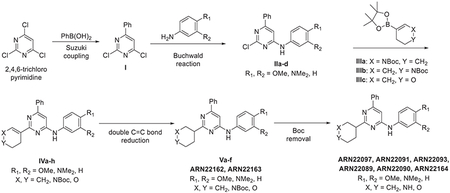


##### Synthesis of compound ARN22097

###### Preparation of 2,4-dichloro-6-phenylpyrimidine (I).


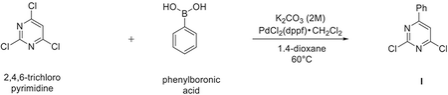


A suspension of 2,4,6-trichloropyrimidine (1000 mg, 5.29 mmol), phenyl boronic acid (665 mg, 5.29 mmol), dichloro[1,1′-bis(diphenylphosphino)ferrocene] palladium dichloromethane complex (204 mg, 0.26 mmol) and K_2_CO_3_ 2 M solution (5.3 mL, 10.58 mmol) in 1,4-dioxane (26.4 mL) was stirred in a CEM® microwave apparatus at 60°C (200 W) for 1 hour. Resulting crude was portioned between dichloromethane (150 mL), NaHCO_3_ saturated solution (100 mL), the organic layer dried over Na_2_SO_4_ and concentrated to dryness at low pressure. Final normal phase purification (cyclohexane/DCM from 100/0 to 85/15) afforded pure title compound (857 mg, yield 72%). Rt = 1.38 min (analysis method 2); MS (ESI) m/z: 225.1 [M-H]^+^, [M-H]^+^ calculated: 225.0. ^1^H NMR (400 MHz, CDCl_3_) *δ* 8.13 – 8.03 (m, 2H), 7.68 (s, 1H), 7.62 – 7.48 (m, 3H).

###### *Representative procedure 1. Preparation of 2-chloro-N*-(4-methoxyphenyl)-6-phenylpyrimidin-4-amine (IIa).


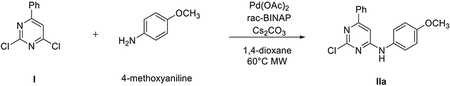


A mixture of Pd(OAc)_2_ (4.5 mg, 0.02 mmol, 0.05 equiv) and *rac*-BINAP (12.5 mg, 0.02 mmol, 0.05 equiv) in 1,4-dioxane (1.3 mL) was stirred under Ar flushing for 10 minutes. Then were stepwise added a solution of intermediate **I** (100 mg, 0.44 mmol, 1 equiv) in 1,4-dioxane (0.44 mL), a solution of 4-methoxyaniline (54.2 mg, 0.44 mmol, 1 equiv) in 1,4-dioxane (0.44 mL) and Cs_2_CO_3_ (172.0 mg, 0.52 mmol, 1.2 equiv). The reaction mixture was stirred in a CEM^®^ microwave apparatus at 60°C (200 W) for 4 hours, filtrated through a celite coarse patch, rinsed with DCM and concentrated to dryness at low pressure. Final normal phase purification (cyclohexane/TBME from 95/5 to 75/25) afforded pure title compound **IIa** (86.8 mg, yield 63 %). Rt = 1.39 min (analysis method 2); MS (ESI) m/z: 312.1 [M-H]^+^, [M-H]^+^ calculated: 312.1. ^1^H NMR (400 MHz, DMSO-*d_6_*) *δ* 9.91 (s, 1H), 8.06 – 7.84 (m, 2H), 7.65 – 7.37 (m,5H), 7.06 (s, 1H), 7.02 – 6.92 (m, 2H), 3.76 (s, 3H).

###### Preparation of tert-butyl 5-(4,4,5,5-tetramethyl-1,3,2-dioxaborolan-2-yl)-3,4-dihydropyridine-1(2H)-carboxylate (IIIa).


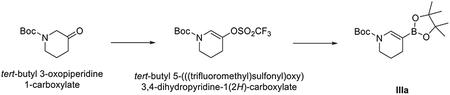


####### Step1: Preparation of tert-butyl 5-(((trifluoromethyl)sulfonyl)oxy)-3,4-dihydropyridine-1(2H)-carboxylate.

At −78°C, to a solution of lithium diisopropylamide 2.0 M in cyclohexane (8.8 mL, 17.53 mmol) in dry tetrahydrofuran (15.6 mL) was added dropwise a solution of 3-oxo-piperidine-1- carboxyilic acid tert-butyl ester (3000 mg, 14.60 mmol) in dry tetrahydrofuran (15.6 mL). The mixture was stirred at −78°C for 1 h and a solution of N-phenyl bis trifluoromethanesulfonamide (5855.6 mg, 16.07 mmol) in dry tetrahydrofuran (15.8 mL) was added. The mixture was stirred at −78°C for 2 h and then was allowed to warm up to room temperature and stirred 16 additional hours at room temperature. The mixture was evaporated to dryness and the residue was taken with diethyl ether (50 mL), washed with water (50 mL), a 2 M solution of sodium hydroxide (50 mL) and brine (50 mL), dried over sodium sulfate and concentrated to dryness at low pressure. Final normal phase purification (cHexane/DCM from 100/0 to 50/50) afforded pure title compound (1354 mg, 28% yield). Rt = 2.66 min (analysis method 1). ^1^H NMR (400 MHz, CDCl_3_) *δ* 7.07 (s, 1H), 3.52 (s, 2H), 2.43 (td, *J* = 6.4, 1.5 Hz, 2H), 1.93 (tt, *J* = 6.3, 5.0 Hz, 2H), 1.49 (s, 9 H).

####### Step2: Preparation of tert-butyl 5-(4,4,5,5-tetramethyl-1,3,2-dioxaborolan-2-yl)-3,4-dihydropyridine-1(2H)-carboxylate (IIIa).

To a degassed solution of sulfonate derived from step 1 (600 mg, 1.81 mmol) in 1,4-dioxane (10.7 mL) was added bis-(pinacolato)diboron (603.8 mg, 2.35 mmol), potassium acetate (502.7 mg, 5.07 mmol) and dichloro[1,1′-bis(diphenylphosphino)ferrocene] palladium dichloromethane complex (139.5 mg, 0.18 mmol) were added. The mixture was stirred at 80°C for 3 h. After cooling down, the mixture was filtered and resulting filtrate concentrated to dryness at low pressure. Final normal phase purification (cHexane/DCM from 70/30 to 50/50) afforded pure title compound **IIIa** (448 mg, 80% yield). Rt = 1.85 min (analysis method 2). MS (ESI) m/z 310.2 [M-H]^+^, [M-H]^+^ calculated: 310.2.^1^H NMR (400 MHz, CDCl_3_) *δ* 5.29 (s, 1H), 3.67–3.39 (m, 2H), 2.15 – 1.96 (m, 2H), 1.81 – 1.73 (m, 2H), 1.49 (s, 9H), 1.32 – 1.17 (m, 12H).

###### Representative procedure 2. Preparation of tert-butyl 5-(4-((4-methoxyphenyl)amino)-6-phenylpyrimidin-2-yl)-3,4-dihydropyridine-1(2H)-carboxylate (IVa).


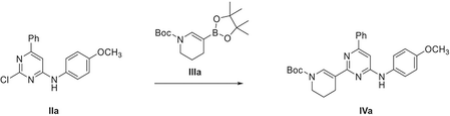


A suspension of intermediate **IIa** (100 mg, 0.32 mmol, 1 equiv), 4,4,5,5-tetramethylboronate **IIIa** (119.0 mg, 0.38 mmol, 1.2 equiv dichloro[1,1′-bis(diphenylphosphino)ferrocene] palladium dichloromethane complex (26.1 mg, 0.03 mmol, 0.1 equiv) and K_2_CO_3_ 2M solution (0.32 mL, 0.64 mmol, 2 equiv) in 1,4-dioxane (10 mL) was stirred in a CEM^®^ microwave apparatus at 120 °C (200 W) for 2 hours. Resulting crude was portioned between dichloromethane (25 mL), NaHCO_3_ saturated solution (25 mL), the organic layer dried over Na_2_SO_4_ and concentrated to dryness at low pressure. Final normal phase purification (cyclohexane/AcOEt from 100/0 to 80/20) afforded pure title compound **IVa** (64.7 mg, yield 44%). Rt = 2.37 min (analysis method 2); MS (ESI) m/z 459.6 [M-H]^+^, [M-H]^+^ calculated: 459.2. ^1^H NMR (400 MHz, CDCl_3_) *δ* 8.05–7.97 (m, 2H), 7.46 – 7.37 (m, 3H), 7.31 (t, *J* = 6.5 Hz, 2H), 6.98 – 6.90 (m, 2H), 6.71 (s, 1H), 6.68 – 6.46 (m, 1H), 3.84 (s, 3H), 3.66 (d, *J* = 9.5 Hz, 2H), 2.82 – 2.60 (m, 2H), 2.02 – 1.86 (m, 2H), 1.56 (s, 9H). *Representative procedure 3. Preparation of tert-butyl 3-(4-((4-methoxyphenyl)amino)-6-phenylpyrimidin-2-yl)piperidine-1-carboxylate (Va).*


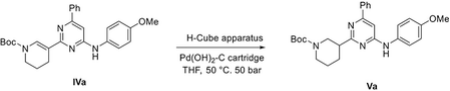


A solution of intermediate **IVa** (65.0 mg, 0.14 mmol) in THF (1.4 mL) was eluted in a H-cube apparatus through a Pd(OH)_2_-C cartridge at 50°C under 50 bar H_2_ pressure until reaction completion. Final normal phase purification (cHexane/TBME from 100/0 to 80/20) afforded pure title compound **Va** (40 mg, yield 62%). Rt = 1.94 min (analysis method 2); MS (ESI) m/z: 461.2 [M-H]^+^, [M-H]^+^ calculated: 461.2. ^1^H NMR (400 MHz, CDCl_3_) *δ* 8.07 – 7.80 (m, 2H), 7.46 – 7.38 (m, 3H), 7.26 (s, 1H), 7.01 – 6.89 (m, 2H), 6.77 (s, 1H), 6.71 (s, 1H), 3.84 (s, 3H), 3.01 – 2.68 (m, 2H), 2.23 (d, *J* = 12.3 Hz, 1H), 1.80 (qd, *J* = 13.0, 3.8 Hz, 2H), 1.73 – 1.52 (m, 4H), 1.47 (s, 9H).

###### Representative procedure 4. Preparation of N-(4-methoxyphenyl)-6-phenyl-2-(piperidin-3-yl)pyrimidin-4-amine (ARN22097).


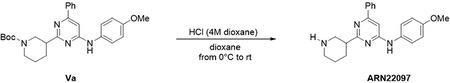


To a 0°C solution of protected intermediate **Va** (65.0 mg, 0.14 mmol, 1 equiv) in 1,4-dioxane (0.36 mL) was dropwise added a HCl (4M) solution in 1,4-dioxane (0.35 mL, 1.4 mmol, 10 equiv) and the reaction mixture stirred at room temperature for 1 h, then the reaction crude was concentrated to dryness at low pressure, the resulting crude portioned between DCM (4 mL) and NaOH 0.1 M (4 mL), the organic layer dried over Na_2_SO_4_ and concentrated to dryness at low pressure. Final normal phase purification (elution by gradient from 85/15 to 65:35 DCM/DCM:NH_3_ 1M MeOH 4:1) afforded pure title compound **ARN22097** (31 mg, yield 99%). Rt = 0.49 min (analysis method 2); MS (ESI) m/z: 361.6 [M-H]^+^, [M-H]^+^ calculated: 361.2. ^1^H NMR (400 MHz, DMSO-*d_6_*) *δ* 9.60 (s, 1H), 8.15 – 7.90 (m, 2H), 7.59 (d, *J* = 8.4 Hz, 2H), 7.56 – 7.47 (m, 3H), 7.05 (d, *J* = 4.2 Hz, 1H), 7.00 – 6.90 (m, 2H), 3.76 (s, 3H), 3.59 (dd, *J* = 18.0, 10.2 Hz, 1H), 3.27 – 3.18 (m, 3H), 2.94 – 2.87 (m, 1H), 2.25 – 2.19 (m, 1H), 1.91 – 1.83 (m, 2H), 1.79-1.69 (m, 1H). ^13^C NMR (100 MHz, DMSO-*d_6_*) *δ* 168.5 (Cq), 161.3 (Cq), 161.0 (Cq), 155.0 (Cq), 137.1 (Cq), 132.8 (Cq), 130.2 (CH), 128.8 (CH, 2C), 126.4 (CH, 2C), 121.7 (CH, 2C), 114.0 (CH, 2C), 99.4 (CH), 55.2 (CH_3_), 46.1 (CH_2_), 43.1 (CH_2_), 41.1 (CH), 27.9 (CH_2_), 21.5 (CH_2_).

##### Synthesis of compound ARN22093

###### *Preparation of N*1-(2-chloro-6-phenylpyrimidin-4-yl)-*N*4,*N*4-dimethylbenzene-1,4-diamine (IIb).


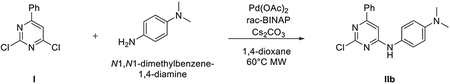


Compound **IIb** was prepared according Representative Procedure 1: using intermediate **I** (300 mg, 1.33 mmol) and *N*1,*N*1-dimethylbenzene-1,4-diamine (191.1 mg, 1.33 mmol). Final normal phase purification (cyclohexane/TBME from 100/0 to 80/20) afforded pure title compound **IIb** (272 mg, yield 63%). Rt = 1.56 min (analysis method 2); MS (ESI) m/z: 325.1 [M-H]^+^, [M-H]^+^ calculated: 325.1. ^1^H NMR (400 MHz, DMSO-*d_6_*) *δ* 9.76 (s, 1H), 7.93 (dd, J = 6.7, 3.0 Hz, 2H), 7.52 (dd, J = 4.6, 2.4 Hz, 3H), 7.36 (s, 2H), 7.00 (s, 1H), 6.87 – 6.64 (m, 2H), 2.89 (s, 6H).

###### Preparation of tert-butyl 5-(4-((4-(dimethylamino)phenyl)amino)-6-phenylpyrimidin-2-yl)-3,4-dihydropyridine-1(2H)-carboxylate (IVb)


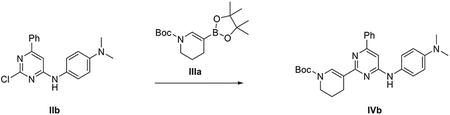


Compound **IVb** was prepared according Representative Procedure 2: using intermediates **IIb** (100 mg, 0.35 mmol) and **IIIa** (117.8 mg, 0.37 mmol). Final normal phase purification (cyclohexane/AcOEt from 100/0 to 85/15) afforded pure title compound **IVb** (107.4 mg, yield 74%). Rt = 2.58 min (analysis method 2); MS (ESI) m/z 472.4 [M-H]^+^, [M-H]^+^ calculated: 472.3. ^1^H NMR (400 MHz, DMSO-*d_6_*) *δ* 9.28 (s, 1H), 8.12 – 7.96 (m, 2H), 7.64 – 7.42 (m, 5H), 7.15 (s, 1H), 6.96 (s, 1H), 6.86 – 6.69 (m, 2H), 4.11 (d, *J* = 4.0 Hz, 2H), 3.55 (t, *J* = 5.7 Hz, 2H), 2.87 (s, 6H), 2.67 (d, *J* = 7.0 Hz, 2H), 1.44 (s, 9H).

###### Preparation of tert-butyl 3-(4-((4-(dimethylamino)phenyl)amino)-6-phenylpyrimidin-2-yl)piperidine-1-carboxylate (Vb).


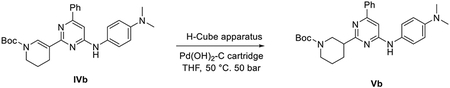


Compound **Vb** was prepared according Representative Procedure 3: using intermediate **IVb** (60.5 mg, 0.12 mmol). Final normal phase purification (cHexane/TBME from 90/10 to 70:30) afforded pure title compound **Vb** (60 mg, yield 99%). Rt = 2.22 min (analysis method 2); MS (ESI) m/z: 474.4 [M-H]^+^, [M-H]^+^ calculated: 474.3. ^1^H NMR (400 MHz, DMSO-*d_6_*) *δ* 9.38 (s, 1H), 8.24 – 7.80 (m, 2H), 7.59 – 7.34 (m, 5H), 6.98 (s, 1H), 6.91 – 6.59 (m, 2H), 3.37 (d, *J* = 9.8 Hz, 1H), 3.16 – 3.02 (m, 1H), 3.02 – 2.91 (m, 2H), 2.87 (s, 6H), 2.68 (td, *J* = 11.9, 3.0 Hz, 1H), 2.23 – 2.05 (m, 1H), 1.89 – 1.70 (m, 2H), 1.63 (q, *J* = 12.8 Hz, 1H), 1.45 (s, 9H).

###### *Preparation of N1,N1*-dimethyl-*N4*-(6-phenyl-2-(piperidin-3-yl)pyrimidin-4-yl)benzene-1,4-diamine (ARN22093).


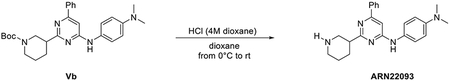


Compound ARN22093 was prepared according Representative Procedure 4: using intermediate **Vb** (61 mg, 0.13 mmol), and HCl (4M) solution in 1,4-dioxane (0.35 mL) in 1,4-dioxane (0.36 mL). Final normal phase purification (elution by gradient from 80/20 to 60/40 DCM/DCM:NH_3_ 1M MeOH 4:1) afforded pure title compound **ARN22093** (46 mg, yield 95%). UPLC_MS: Rt = 1.96 min (analysis method 1); MS (ESI) m/z: 374.6 [M-H]^+^, [M-H]^+^ calculated: 374.2. ^1^H NMR (400 MHz, DMSO-*d_6_*) *δ* 9.38 (s, 1H), 7.99–7.96 (m, 2H), 7.52 – 7.44 (m, 5H), 6.98 (s, 1H), 6.67 – 6.33 (m, 2H), 3.37 (d, *J* = 9.8 Hz, 1H), 3.16 – 3.02 (m, 1H), 3.00 – 2.90 (m, 2H), 2.87 (s, 6H), 2.68 (td, *J* = 12.1, 3.0 Hz, 1H), 2.16 – 2.11 (m, 1H), 1.89 – 1.58 (m, 3H). ^13^C NMR (100 MHz, DMSO-*d_6_*) *δ* 170.2 (Cq), 161.4 (Cq), 160.8 (Cq), 146.8 (Cq), 137.4 (Cq), 136.9 (Cq), 129.9 (CH), 128.7 (CH, 2C), 126.3 (CH, 2C), 121.9 (CH, 2C), 112.9 (CH, 2C), 98.6 (CH), 49.0 (CH_2_), 44.9 (CH_2_), 44.0 (CH), 40.5 (CH_3_, 2C), 29.0 (CH_2_), 24.1 (CH_2_).

##### Total synthesis of compound ARN22091

###### *Preparation of 2-chloro-N*-(3-methoxyphenyl)-6-phenylpyrimidin-4-amine (IIc).


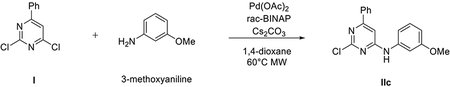


Compound **IIc** was prepared according Representative Procedure 1: using intermediate **I** (300 mg, 1.13 mmol) and 3-methoxyaniline (154 μL, 1.33 mmol). Final normal phase purification (cyclohexane/TBME from 100/0 to 85/15) afforded pure title compound **IIc** (150 mg, yield 36%). Rt = 1.52 min (analysis method 2); MS (ESI) m/z: 312.1 [M-H]^+^, [M-H]^+^ calculated: 312.1. ^1^H NMR (400 MHz, DMSO-*d_6_*) *δ* 10.07 (s, 1H), 8.00–7.90 (m, 2H), 7.59 – 7.50 (m, 3H), 7.34 (t, *J* = 2.3 Hz, 1H), 7.29 (t, *J* = 8.1 Hz, 1H), 7.18 (s, 2H), 6.69 (ddd, *J* = 8.2, 2.5, 0.9 Hz, 1H), 3.77 (s, 3H).

###### Preparation of tert-butyl 5-(4-((3-methoxyphenyl)amino)-6-phenylpyrimidin-2-yl)-3,4-dihydropyridine-1(2H)-carboxylate (IVc)


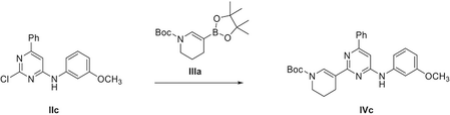


Compound **IVc** was prepared according Representative Procedure 2: using intermediate **IIc** (100 mg, 0.32 mmol) and intermediate **IIIa** (119.0 mg, 0.38 mmol) following the general procedure reaction C previously described. Final normal phase purification (cyclohexane/TBME from 100/0 to 80/20) afforded pure title compound **IVc** (65.0 mg, yield 44%). Rt = 2.40 min (method 2); MS (ESI) m/z 459.6 [M-H]^+^, [M-H]^+^ calculated: 459.2.

###### Preparation of tert-butyl 3-(4-((3-methoxyphenyl)amino)-6-phenylpyrimidin-2-yl)piperidine-1-carboxylate (Vc).


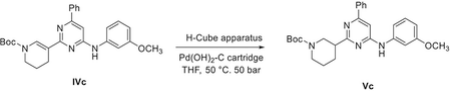


Compound **Vc** was prepared according Representative Procedure 3: using intermediate **IVc** (65.0 mg, 0.14 mmol). Final normal phase purification (cHexane/AcOEt from 100/0 to 80:20) afforded pure title compound (32.6 mg, yield 50%). Rt = 2.13 min (analysis method 2); MS (ESI) m/z: 461.6 [M-H]^+^, [M-H]^+^ calculated: 461.2. ^1^H NMR (400 MHz, CDCl_3_) *δ* 8.04 – 7.92 (m, 2H), 7.45 (p, *J* = 3.9, 3.2 Hz, 3H), 7.30 (t, *J* = 8.1 Hz, 1H), 7.08 (t, *J* = 2.2 Hz, 1H), 6.99 (s, 1H), 6.95 (dd, *J* = 7.9, 2.0 Hz, 1H), 6.89 (s, 1H), 6.72 (dd, *J* = 8.3, 2.4 Hz, 1H), 3.84 (s, 3H), 3.20 (s, 1H), 2.99 – 2.88 (m, 1H), 2.80 (t, *J* = 12.5 Hz, 1H), 2.26 (d, *J* = 12.5 Hz, 1H), 1.93 – 1.74 (m, 2H), 1.75 – (m, 3H), 1.47 (s, 9H).

###### *Preparation of N*-(3-methoxyphenyl)-6-phenyl-2-(piperidin-3-yl)pyrimidin-4-amine (ARN22091).


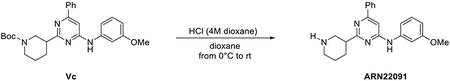


Compound ARN22091 was prepared according Representative Procedure 4: using intermediate **Vc**, (61 mg, 0.13 mmol), and HCl (4M) solution in 1,4-dioxane (0.35 mL) in 1,4- (0.36). Final normal phase purification (elution by gradient from 85/15 to 60/40 DCM/DCM:NH_3_ 1M MeOH 4:1) afforded pure **ARN22091** (39 mg, yield 82%). UPLC_MS: Rt = 1.91 min (analysis method 1); MS (ESI) m/z: 361.6 [M-H]^+^, [M-H]^+^ calculated: 361.2. ^1^H NMR (400 MHz, DMSO-*d_6_*) 9.89 (s, 1H), 8.17–7.94 (m, 2H), 7.67 – 7.44 (m, 4H), 7.34 – 7.12 (m, 3H), 6.61 (dt, *J* = 5.4, 2.4 Hz, 1H), 3.79 (s, 3H), 3.63 (d, *J* = 8.1 Hz, 1H), 3.27 – 3.18 (m, 3H), 2.89 (d, *J* = 14.1 Hz, 1H), 2.26 (d, *J* = 11.7 Hz, 1H), 2.01 – 1.67 (m, 3H). ^13^C NMR (100 MHz, DMSO-*d_6_*) *δ* 168.5 (Cq), 161.3 (Cq), 161.1 (Cq), 159.6 (Cq), 141.2 (Cq), 136.9 (Cq), 130.3 (CH), 129.4 (CH), 128.8 (CH, 2C), 126.4 (CH, 2C), 111.8 (CH), 108.0 (CH), 105.1 (CH), 100.5 (CH), 55.0 (CH_3_), 46.1 (CH_2_), 43.1 (CH_2_), 41.2 (CH), 27.9 (CH_2_), 21.5 (CH_2_).

##### Total synthesis of compound ARN22164

###### *Preparation of N1*-(2-chloro-6-phenylpyrimidin-4-yl)-*N3,N3*-dimethylbenzene-1,3-diamine.


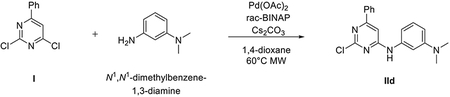


Compound **IId** was prepared according Representative Procedure 1: using intermediate **I** (300 mg, 1.13 mmol) and N1,N1-dimethylbenzene-1,3-diamine (181.5 mg, 1.33 mmol). Final normal phase purification (cyclohexane/TBME from 100/0 to 80/20) afforded pure title compound (246 mg, yield 57%). Rt = 1.73 min (analysis method 2); MS (ESI) m/z: 325.1 [M-H]^+^, [M-H]^+^ calculated: 325.1. ^1^H NMR (400 MHz, DMSO-*d_6_*) *δ* 9.93 (s, 1H), 8.14 – 7.82 (m, 2H), 7.60 – 7.47 (m, 3H), 7.29 – 7.09 (m, 2H), 7.04 (s, 1H), 6.99 – 6.85 (m, 1H), 6.51 (ddd, *J* = 8.4, 2.5, 0.8 Hz, 1H), 2.92 (s, 6H).

###### Preparation of tert-butyl 5-(4-((3-(dimethylamino)phenyl)amino)-6-phenylpyrimidin-2-yl)-3,4-dihydropyridine-1(2H)-carboxylate (IVd).


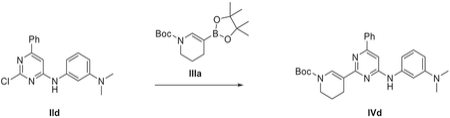


Compound **IVd** was prepared according Representative Procedure 2: using **IId** (175 mg, 0.54 mmol) and intermediate **IIIa** (199.9 mg, 0.65 mmol). Final normal phase purification (cyclohexane/AcOEt from 100/0 to 85/15) afforded pure title compound (109.2 mg, yield 43%). Rt = 2.65 min (analysis method 2); MS (ESI) m/z 472.3 [M-H]^+^, [M-H]^+^ calculated: 472.3. ^1^H NMR (400 MHz, DMSO-*d_6_*) *δ* 9.34 (s, 1H), 8.40 (s, 1H), 8.04 (dd, *J* = 7.7, 1.9 Hz, 2H), 7.59 – 7.44 (m, 4H), 7.11 (t, *J* = 8.0 Hz, 2H), 6.99 (s, 1H), 6.40 (dd, *J* = 9.1, 2.5 Hz, 1H), 3.59 (t, *J* = 5.6 Hz, 2H), 2.92 (s, 6H), 2.65 – 2.59 (m, 2H), 1.88 (p, *J* = 6.0 Hz, 2H), 1.50 (s, 9H).

###### Preparation of tert-butyl 3-(4-((3-(dimethylamino)phenyl)amino)-6-phenylpyrimidin-2-yl)piperidine-1-carboxylate (Vd).


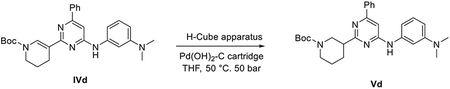


Compound **Vd** was prepared according Representative Procedure 3: using intermediate **IVd** (105 mg, 0.22 mmol). Final normal phase purification (cHexane/AcOEt from 100/0 to 80/20) afforded pure title compound Vd (12 mg, yield 12 %). Rt = 2.35 min (analysis method 2); MS (ESI) m/z: 474.6 [M-H]^+^, [M-H]^+^ calculated: 474.3. ^1^H NMR (400 MHz, CDCl_3_) *δ* 8.04 – 7.93 (m, 2H), 7.49 – 7.37 (m, 4H), 7.04 (s, 1H), 6.78 (s, 1H), 6.69 (d, *J* = 7.7 Hz, 1H), 6.59 (d, *J* = 8.5 Hz, 1H), 4.20 – 4.08 (m, 1H), 3.23 – 3.16 (m, 1H), 2.99 (s, 6 H), 2.98 – 2.91 (m, 1H), 2.88 – 2.77 (m, *J* = 14.3 Hz, 1H), 2.31 – 2.20 (m, 1H), 1.82 – 1.58 (m, 2H), 1.51 – 1.45 (m, 11H).

###### *Preparation of N1,N1*-dimethyl-*N3*-(6-phenyl-2-(piperidin-3-yl)pyrimidin-4-yl)benzene-1,3-diamine (ARN22164).


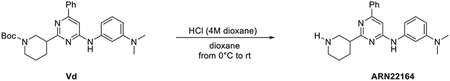


Compound ARN22164 was prepared according Representative Procedure 4: using intermediate **Vd**, (34 mg, 0.08 mmol), and HCl (4M) solution in 1,4-dioxane (0.22 mL) in 1,4-dioxane (0.22). Final normal phase purification (elution by gradient from from 95/5 to 45/55 DCM/DCM:NH_3_ 1M MeOH 4:1) afforded pure title compound **ARN22164** (16 mg, yield 61%). UPLC_MS: Rt = 2.07 min (analysis method 1); MS (ESI) m/z: 374.5 [M-H]^+^, [M-H]^+^ calculated: 374.2. ^1^H NMR (400 MHz, DMSO-*d_6_*) *δ* 9.50 (s, 1H), 8.01 (dd, *J* = 7.8, 1.8 Hz, 2H), 7.56–7.48 (m, 3H), 7.45 (s, 1H), 7.12 (t, *J* = 8.1 Hz, 1H), 7.10 – 7.06 (m, 1H), 6.90 (d, *J* = 8.0 Hz, 1H), 6.40 (dd, *J* = 8.2, 2.5 Hz, 1H), 3.26 – 3.17 (m, 1H), 2.94 (s, 6H), 2.94 – 2.87 (m, 1H), 2.82– 2.75 (m, 2H), 2.46 (dd, *J* = 12.1, 2.9 Hz, 1H), 2.16 – 2.03 (m, 1H), 1.90 – 1.72 (m, 1H), 1.71 – 1.58 (m, 1H), 1.57 – 1.39 (m, 1H). ^13^C NMR (100 MHz, DMSO-*d_6_*) *δ* 171.5 (Cq), 161.3 (Cq), 160.9 (Cq), 150.9 (Cq), 141.0 (Cq), 137.4 (Cq), 130.0 (CH), 129.0 (CH), 128.8 (CH, 2C), 126.4 (CH, 2C), 107.8 (CH), 106.7 (CH), 103.8 (CH), 99.7 (CH), 51.4 (CH_2_), 46.4 (CH), 46.3 (CH_2_), 40.3 (CH_3_, 2C), 30.0 (CH_2_), 26.1 (CH_2_).

##### Total synthesis of compound ARN22090

###### Preparation of tert-butyl 4-(4-((3-methoxyphenyl)amino)-6-phenylpyrimidin-2-yl)-3,6-dihydropyridine-1(2H)-carboxylate (IVe).


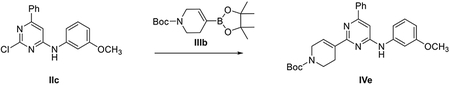


Compound **IVe** was prepared according Representative Procedure 2: using intermediate **IIc** (180 mg, 0.58 mmol) and tert-butyl 4-(4,4,5,5-tetramethyl-1,3,2-dioxaborolan-2-yl)-3,6-dihydro-2H-pyridine-1-carboxylate **IIIb** (202.4 mg, 0.64 mmol). Final normal phase purification (cyclohexane/AcOEt from 100/0 to 85/15) afforded title compound **IVe** (241.2 mg, yield 91%). Rt = 2.31 min (analysis method 2); MS (ESI) m/z 459.3 [M-H]^+^, [M-H]^+^ calculated: 459.2. ^1^H NMR (400 MHz, DMSO-*d_6_*) *δ* 9.63 (s, 1H), 8.19 – 8.03 (m, 2H), 7.62 – 7.46 (m, 4H), 7.33 – 7.22 (m, 2H), 7.20 (s, 1H), 7.11 (s, 1H), 6.67 – 6.54 (m, 1H), 4.13 (d, *J* = 3.3 Hz, 2H), 3.79 (s, 3H), 3.57 (t, *J* = 5.7 Hz, 2H), 2.71 (s, 2H), 1.44 (s, 9H).

###### Representative Procedure 5. Preparation of tert-butyl 4-(4-((3-methoxyphenyl)amino)-6-phenylpyrimidin-2-yl)piperidine-1-carboxylate (Ve).


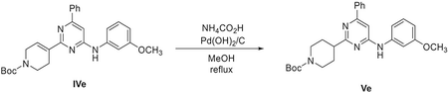


Under N_2_ atmosphere, a suspension of intermediate **IVe** (240 mg, 0.52 mmol, 1 equiv), ammonium formate (131 mg, 2.08 mmol, 4 equiv), Pd(OH)_2_/C (48 mg, 20% of starting material weight) in MeOH dry was stirred at reflux temperature until reaction completion. Catalyst was filtered off through a celite coarse patch and resulting filtrate concentrated to dryness at low pressure. Final normal phase purification (cyclohexane/AcOEt from 100/0 to 80/20) afforded pure title compound **Ve** (120 mg, yield 50%). Rt = 2.11 min (analysis method 2); MS (ESI) m/z: 461.4 [M-H]^+^, [M-H]^+^ calculated: 461.2. ^1^H NMR (400 MHz, DMSO-*d_6_*) *δ* 9.61 (s, 1H), 8.07 – 7.94 (m, 2H), 7.63 (t, *J* = 2.2 Hz, 1H), 7.57 – 7.45 (m, 3H), 7.22 (t, *J* = 8.0 Hz, 1H), 7.17 (dt, *J* = 8.3, 1.4 Hz, 1H), 7.08 (s, 1H), 6.59 (ddd, *J* = 8.0, 2.5, 1.1 Hz, 1H), 4.12 – 3.96 (m, 2H), 3.77 (s, 3H), 2.93 (tt, *J* = 11.4, 3.7 Hz, 3H), 2.06 – 1.95 (m, 2H), 1.72 (qd, *J* = 12.4, 4.2 Hz, 2H), 1.42 (s, 9H).

###### *Preparation of N*-(3-methoxyphenyl)-6-phenyl-2-(piperidin-4-yl)pyrimidin-4-amine (ARN22090).


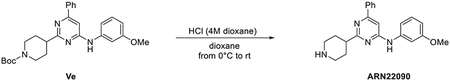


Compound **ARN22090** was prepared according Representative Procedure 4: using intermediate **Ve**, (115 mg, 0.25 mmol), and HCl (4M) solution in 1,4-dioxane (0.69 mL) in 1,4-dioxane (0.69). Final normal phase purification (elution by gradient from from 95/5 to 50/50 DCM/DCM:NH_3_ 1M MeOH 4:1) afforded pure title compound **ARN22090** (80.1 mg, yield 89%). UPLC_MS: Rt = 1.85 min (analysis method 1); MS (ESI) m/z: 361.6 [M-H]^+^, [M-H]^+^ calculated: 361.2. ^1^H NMR (400 MHz, DMSO-*d_6_*) *δ* 9.58 (s, 1H), 8.04 – 8.00 (m, 2H), 7.65 (bs, 1H), 7.55 – 7.47 (m, 3H), 7.25 – 7.19 (m, 2H), 7.07 (s, 1H), 6.58 (dt, *J* = 7.2, 2.3 Hz, 1H), 3.78 (s, 3H), 3.04 (dt, *J* = 12.2, 3.3 Hz, 2H), 2.80 (tt, *J* = 11.9, 3.9 Hz, 1H), 2.61 (td, *J* = 11.9, 2.5 Hz, 2H), 2.03 – 1.85 (m, 2H), 1.74 (qd, *J* = 12.2, 4.0 Hz, 2H). ^13^C NMR (100 MHz, DMSO-*d_6_*) *δ* 172.5 (Cq), 161.3 (Cq), 161.2 (Cq), 159.6 (Cq), 141.5 (Cq), 137.3 (Cq), 130.1 (CH), 129.4 (CH), 128.8 (CH, 2C), 126.4 (CH, 2C), 111.6 (CH), 107.6 (CH), 105.0 (CH), 99.7 (CH), 55.0 (CH_3_), 46.1 (CH_2_, 2C), 45.6 (CH), 31.8 (CH_2_, 2C).

##### Synthesis of compound ARN22089

###### Preparation of tert-butyl 4-(4-((3-(dimethylamino)phenyl)amino)-6-phenylpyrimidin-2-yl)-3,6-dihydropyridine-1(2H)-carboxylate (IVf).


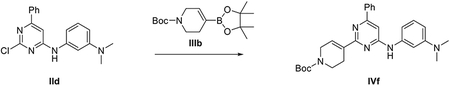


Compound **IVf** was prepared according Representative Procedure 2: using intermediate **IId** (120 mg, 0.37 mmol) and tert-butyl 4-(4,4,5,5-tetramethyl-1,3,2-dioxaborolan-2-yl)-3,6-dihydro-2H-pyridine-1-carboxylate **IIIb** (129.5 mg, 0.41 mmol). Final normal phase purification (cyclohexane/AcOEt from 100/0 to 80/20) afforded title compound **IVf** (155.1 mg, yield 89%). Rt = 2.48 min (analysis method 2); MS (ESI) m/z 472.3 [M-H]^+^, [M-H]^+^ calculated: 472.3.^1^H NMR (400 MHz, DMSO-*d_6_*) *δ* 9.44 (s, 1H), 8.17–7.95 (m, 2H), 7.67 – 7.41 (m, 3H), 7.31 (s, 1H), 7.21 (s, 1H), 7.14 (t, *J* = 8.1 Hz, 1H), 7.11 (s, 1H), 7.03 – 6.93 (m, 1H), 6.42 (ddd, *J* = 8.3, 2.7, 0.8 Hz, 1H), 4.11 (s, 2H), 3.56 (t, *J* = 5.6 Hz, 2H), 2.93 (s, 6H), 2.81 – 2.62 (m, 2H), 1.44 (s, 9H).

###### Preparation of tert-butyl 4-(4-((3-(dimethylamino)phenyl)amino)-6-phenylpyrimidin-2-yl)piperidine-1-carboxylate (Vf).


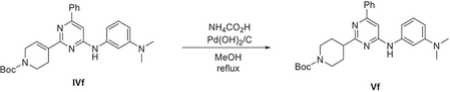


Compound **Vf** was prepared according Representative Procedure 5: using intermediate **IVf** (150 mg, 0.32 mmol). Final normal phase purification (cyclohexane/AcOEt from 100/0 to 80/20) afforded pure title compound **Vf** (149 mg, yield 99%). Rt = 2.30 min (analysis method 2); MS (ESI) m/z: 474.4 [M-H]^+^, [M-H]^+^ calculated: 474.3. ^1^H NMR (400 MHz, DMSO-*d_6_*) *δ* 9.43 (s, 1H), 8.08 – 7.94 (m, 2H), 7.58 – 7.44 (m, 3H), 7.40 (s, 1H), 7.11 (t, *J* = 8.1 Hz, 1H), 7.06 (s, 1H), 6.88 (ddd, *J* = 7.9, 2.1, 0.8 Hz, 1H), 6.41 (ddd, *J* = 8.4, 2.5, 0.8 Hz, 1H), 4.05 (d, *J* = 13.1 Hz, 2H), 1.98 (dd, *J* = 13.6, 3.5 Hz, 2H), 2.95 – 2.87 (m, 9H), 1.73 (qd, *J* = 12.5, 4.2 Hz, 2H), 1.42 (s, 9H).

###### *Preparation of N1,N1*-dimethyl-*N3*-(6-phenyl-2-(piperidin-4-yl)pyrimidin-4-yl)benzene-1,3-diamine (ARN22089).


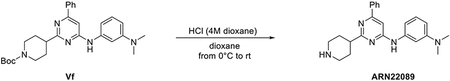


Compound ARN22089 was prepared according Representative Procedure 4: using intermediate **Vf**, (80 mg, 0.22 mmol), and HCl (4M) solution in 1,4-dioxane (0.61 mL) in 1,4-dioxane (0.61). Final normal phase purification (elution by gradient from from 95/5 to 60/40 DCM/DCM:NH_3_ 1M MeOH 4:1) afforded pure title compound ARN22089 (39.6 mg, yield 34%). UPLC_MS: Rt = 1.97 min (analysis method 1); MS (ESI) m/z: 374.6 [M-H]^+^, [M-H]^+^ calculated: 374.2. ^1^H NMR (400 MHz, DMSO-*d_6_*). *δ* 9.42 (s, 1H), 8.01 (d, *J* = 7.0 Hz, 2H), 7.51 (d, *J* = 7.0 Hz, 3H), 7.40 (s, 1H), 7.12 (t, *J* = 8.1 Hz, 1H), 7.07 (s, 1H), 6.94 (d, *J* = 8.0 Hz, 1H), 6.51 – 6.33 (m, 1H), 3.03 (d, *J* = 11.9 Hz, 2H), 2.93 (s, 6H), 2.85 – 2.70 (m, 1H), 2.61 (t, *J* = 11.9 Hz, 2H), 2.00 – 1.85 (m, 2H), 1.75 (qd, *J* = 12.3, 4.3 Hz, 2H). ^13^C NMR (100 MHz, DMSO-*d_6_*) *δ* 161.9 (Cq), 161.5 (Cq), 151.4 (Cq), 141.4 (Cq), 137.9 (Cq), 130.5 (CH), 129.5 (CH), 129.2 (CH, 2C), 126.4 (CH, 2C), 108.3 (CH), 107.2 (CH), 104.3 (CH), 100.0 (CH), 45.8 (CH_2_, 2C), 45.2 (CH), 40.2 (CH_3_), 31.4 (CH_2_, 2C).

##### Synthesis of ARN22162

###### *Preparation of N*-(4-methoxyphenyl)-6-phenyl-2-tetrahydropyran-4-yl-pyrimidin-4-amine (IVg).


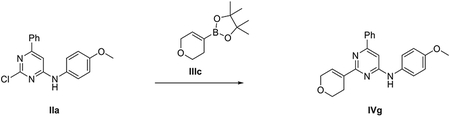


Compound **IVg** was prepared according Representative Procedure 2: using intermediate **IIa** (90 mg, 0.29 mmol) and 3,6-Dihydro-2H-pyran-4-boronic acid pinacol ester (74.3 mg, 0.35 mmol). Final normal phase purification (cyclohexane/TBME from 100/0 to 80/20) afforded title compound (44.2 mg, yield 43%). Rt = 1.54 min (analysis method 2); MS (ESI) m/z 360.5 [M-H]^+^, [M-H]^+^ calculated: 360.2. ^1^H NMR (400 MHz, DMSO-*d_6_*) *δ* 9.45 (s, 1H), 8.12 - 8.01 (m, 2H), 7.65 (d, J = 8.9 Hz, 2H), 7.57 - 7.45 (m, 5H), 7.01 (s, 1H), 6.98 - 6.92 (m, 1H), 4.33 (d, J = 2.9 Hz, 2H), 3.76 - 3.72 (m, 5H), 2.71 - 2.61 (m, 2H).

###### Preparation of N-(4-methoxyphenyl)-6-phenyl-2-tetrahydropyran-4-yl-pyrimidin-4-amine (ARN22162).


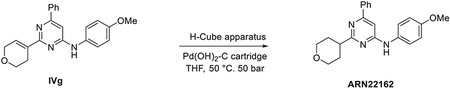


Compound ARN22162 was prepared according Representative Procedure 3: using intermediate **IVg** (42 mg, 0.12 mmol). Final normal phase purification (cyclohexane/TBME from 90/10 to 70/30) afforded pure title compound (16 mg, yield 38%). Rt = 1.31 min (analysis method 2); MS (ESI) m/z: 362.5 [M-H]^+^, [MH]^+^ calculated: 362.2. ^1^H NMR (400 MHz, DMSO-*d_6_*) *δ* 9.43 (s, 1H), 8.11 - 7.88 (m, 2H), 7.63 (d, J = 8.5 Hz, 2H), 7.55 - 7.45 (m, 3H), 6.98 (s, 1H), 6.96 - 6.90 (m, 2H), 3.96 (ddd, J = 11.3, 4.2, 2.3 Hz, 2H), 3.75 (s, 3H), 3.47 (td, J = 11.3, 3.0 Hz, 2H), 3.03 - 2.88 (m, 1H), 1.98 - 1.77 (m, 4H). ^13^C NMR (100 MHz, DMSO-*d_6_*) *δ* 171.7 (Cq), 161.4 (Cq), 161.1 (Cq), 154.8 (Cq), 137.4 (Cq), 133.1 (Cq), 130.1 (CH), 128.8 (CH, 2C), 126.4 (CH, 2C), 121.5 (CH, 2C), 114.1 (CH, 2C), 98.9 (CH), 67.0 (CH_2_, 2C), 55.2 (CH_3_), 43.6 (CH), 31.2 (CH_2_, 2C).

##### Synthesis of ARN22163

###### *Preparation of N1*-[2-(3,6-dihydro-2H-pyran-4-yl)-6-phenyl-pyrimidin-4-yl]-*N4,N4*-dimethyl-benzene-1,4-diamine (IVh).


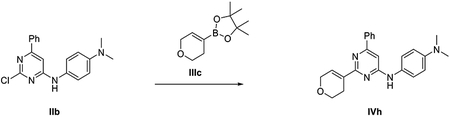


Compound **IVh** was prepared according Representative Procedure 2: using intermediate **IIb** (115 mg, 0.35 mmol) and 3,6-Dihydro-2H-pyran-4-boronic acid pinacol ester (91.1 mg, 0.42 mmol). Final normal phase purification (cyclohexane/AcOEt from 90/10 to 70/30) afforded title compound (37.1 mg, yield 28%). Rt = 1.70 min (analysis method 2); MS (ESI) m/z 373.5 [M-H]^+^, [M-H]^+^ calculated: 373.2. 1H NMR (400 MHz, DMSO-*d_6_*) *δ* 9.29 (s, 1H), 8.03 (dd, J = 7.7, 1.9 Hz, 2H), 7.58 - 7.43 (m, 5H), 7.25 - 7.12 (m, 1H), 6.96 (s, 1H), 6.81 - 6.71 (m, 2H), 4.32 (q, J = 2.7 Hz, 2H), 3.83 (t, J = 5.4 Hz, 2H), 2.87 (s, 6H), 2.71 - 2.58 (m, 2H).

###### *Preparation of N4,N4*-dimethyl-*N1*-(6-phenyl-2-tetrahydropyran-4-yl-pyrimidin-4-yl)benzene-1,4-diamine (ARN22163).


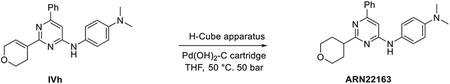


Compound ARN22163 was prepared according Representative Procedure 3: using intermediate **IVh** (35 mg, 0.09 mmol). Final normal phase purification (cyclohexane/AcOEt from 95/5 to 75/25) afforded pure title compound (27 mg, yield 77%). Rt = 1.48 min (analysis method 2); MS (ESI) m/z: 375.5 [M-H]^+^, [MH]^+^ calculated: 375.2. 1H NMR (400 MHz, DMSO-*d_6_*) *δ* 9.27 (s, 1H), 8.05 - 7.91 (m, 2H), 7.63 - 7.40 (m, 4H), 6.93 (s, 1H), 6.83 - 6.68 (m, 2H), 3.96 (dt, J = 11.4, 2.6 Hz, 2H), 3.48 (td, J = 11.1, 3.5 Hz, 2H), 2.93 (dq, J = 10.7, 5.8, 5.3 Hz, 1H), 2.88 (s, 5H), 1.96 - 1.77 (m, 4H). ^13^C NMR (101 MHz, DMSO-*d_6_*) *δ* 171.7 (Cq), 169.4 (Cq), 164.7 (Cq), 142.7 (Cq), 140.6 (Cq), 137.6 (Cq), 130.0 (CH), 128.76 (CH), 126.4 (CH), 113.0 (CH), 98.8 (CH), 66.9 (CH_2_), 43.6 (CH), 40.6 (CH_3_), 31.2 (CH_2_).

### QUANTIFICATION AND STATISTICAL ANALYSIS

Any quantification and statistical analysis that were applied in the experiments are described in Method details, as well as in the main and supplemental figure legends.

## Supplementary Material

1

2

3

4

5

6

## Figures and Tables

**Table:** KEY RESOURCES TABLE

REAGENT or RESOURCE	SOURCE	IDENTIFIER
Antibodies		
GFP Tag Polyclonal Antibody	Thermo Fisher Scientific	Cat# A10262, RRID:AB_2534023
CDC42 Antibody	Cell Signaling Tech	Cat# 2462, RRID:AB_2078085
RHOJ Antibody	Abnova	Cat# H00057381-M01, RRID:AB_2179421
RAC1/2/3 Antibody	Cell Signaling Tech	Cat# 2465, RRID:AB_2176152
S6 Ribosomal Protein (54D2) mouse mAb	Cell Signaling Tech	Cat# 2317, RRID:AB_2238583
Phospho S6 Ribosomal Protein (Ser235/236)	Cell Signaling Tech	Cat# 2211, RRID:AB_331679
p44/42 MAPK (Erk1/2)	Cell Signaling Tech	Cat# 9102, RRID:AB_330744
Phospho p44/42 MAPK (Erk1/2) (Thr202/Tyr204)	Cell Signaling Tech	Cat# 4376, RRID:AB_331772
PAK1 Antibody	Cell Signaling Tech	Cat# 2602, RRID:AB_330222
Phospho-PAK1 (Ser199/204)/PAK2	Cell Signaling Tech	Cat# 2605, RRID:AB_2160222
Vinculin	Cell Signaling Tech	Cat# 4650, RRID:AB_10559207
Beta-actin (13E5) Rabbit mAb	Cell Signaling Tech	Cat# 4970, RRID:AB_2223172
RALB	Cell Signaling Tech	Cat# 3523, RRID:AB_2176036
pan-RAS	Cell Signaling Tech	Cat# 4691, RRID:AB_915783
RelB (D7D7W) Rabbit mAb	Cell Signaling Tech	Cat# 10544, RRID:AB_2797727
Biological samples		
BRAF^V600K^	National Cancer Institute	128128-338-R
BRAF^V600K^, PTPN11^N58S^	National Cancer Institute	563396-261-R
BRAF^V600E^ Vemurafenib Resistant	National Cancer Institute	156681-154-R
BRAF^V600E^, PTEN^H259Y^, TP53^C227Y^	National Cancer Institute	425362-245-T
BRAF^V600E^	National Cancer Institute	174941-126-T
Chemicals, peptides, and recombinant proteins		
FRAX597	Tocris	6029
Linifanib (ABT869)	SellekChem	S1003
Critical commercial assays		
Cdc42 Activation Assay	Cell Biolabs	Sta-402
Ras and Pan-Ras Activation Assays	Cell Biolabs	Sta-400
Rhoa/rac1/cdc42 activation assay combo	Cell Biolabs	Sta-405
Promega CellTiter-Glo Luminescent	Fisher Scientific	G7571
RNesay Mini Kit	Qiagen	74104
Deposited data		
RNA sequencing data	This paper	GEO: GSE197216
Experimental models: Cell lines		
Human: A375	ATCC	CRL-1619
Human: WM3248	Coriell Medical Institute	WC00081
Human: SKMeL28	ATCC	HTB-72
Human: SKMeL3	ATCC	HTB-69
Human: SW480	ATCC	CCL-228
Human: cell-derived endothelial cells	Lab of Hughes	Hachey et al., 2021
Human: Lung Fibroblasts	Lonza	CC-2512
Human: Inducible HEK293 Flp-In T-Rex	Lab of Cote	Bagci et al., 2020
Experimental models: Organisms/strains		
NOD.Cg-*Prkdc^scid^ IL2rg^tm1Wjl^*/SzJ (NSG)	Jackson	#005557
*BRAF^V600E^, PTEN^flox/flox^, Tyr:Cre^ERT2^*	Lab of Ganesan	Ruiz et al., 2017
Recombinant DNA		
pKK-BiFC-Venus	Addgene	105804
pOG44	ThermoFisher Scientific	V600520
mCherry (LeGO-C2)	Addgene	Plasmid#27339
GFP (LeGO-V2)	Addgene	Plasmid#27340
Software and algorithms		
ImageJ	National Institute of Health Bethesda USA	https://imagej.nih.gov/ij/
AngioTool	National Cancer Institute	Zudaire et al., 2011
SPOT	SPOT Imaging	https://www.spotimaging.com/software/
Imaris	BitPlane	https://imaris.oxinst.com/
GraphPad Prism 8.0.0	GraphPad	https://www.graphpad.com
Tophat v2.1 Bowtie2 v2.2.7 Samtools v1.9, v0.1.19 Cufflinks v2.2.1	Lab of Trapnell	Trapnell et al., 2009, 2010
XenofilteR		Kluin et al., 2018
HISAT2 v2.1.0		Kim et al., 2019
featureCounts (subread v1.5.0-p3)		Liao et al., 2014
CummeRbund, R v4.0.3		Goff et al., 2021
DESeq2		Love et al., 2014
LigPrep		Lipinski 2004
QikProp		https://www.schrodinger.com/products/qikprop
Prime		Jacobson et al., 2004
DAVID (‘Database for Annotation, Visualization and Integrated Discovery’)		Szklarczyk et al., 2019
STRING (‘Search Tool for Retrieval of Interacting Genes/Proteins’)		Dennis et al., 2003
TukeyHSD	RDocumentation	https://www.rdocumentation.org/packages/stats/versions/3.6.2/topics/TukeyHSD

## Highlights

- Identified an allosteric pocket at the CDC42/RHOJ effector interaction interface
- Used *in silico* methods to identify and optimize ARN22089, which binds to CDC42
- ARN22089 has broad anti-cancer activity and inhibits MAPK and S6 signaling
- ARN22089 inhibits tumor angiogenesis *in vitro* and tumor growth *in vivo*
