# Supplementary material for: Structure-based design of CDC42 effector interaction inhibitors for the treatment of cancer
Source: Cell Rep. Author manuscript; Available in PMC 2022 May 24. (PMC9127750; doi:10.1016/j.celrep.2022.110641)
Supplement: 1 [file NIHMS1795991-supplement-1.pdf]

Cell Reports, Volume *39*

Supplemental information

Structure-based design of CDC42 effector interaction inhibitors for the treatment of cancer

Sohail Jahid, Jose A. Ortega, Linh M. Vuong, Isabella Maria Acquistapace, Stephanie J. Hachey, Jessica L. Flesher, Maria Antonietta La Serra, Nicoletta Brindani, Giuseppina La Sala, Jacopo Manigrasso, Jose M. Arencibia, Sine Mandrup Bertozzi, Maria Summa, Rosalia Bertorelli, Andrea Armirotti, Rongsheng Jin, Zheng Liu, Chi-Fen Chen, Robert Edwards, Christopher C.W. Hughes, Marco De Vivo, and Anand K. Ganesan

Figure S1

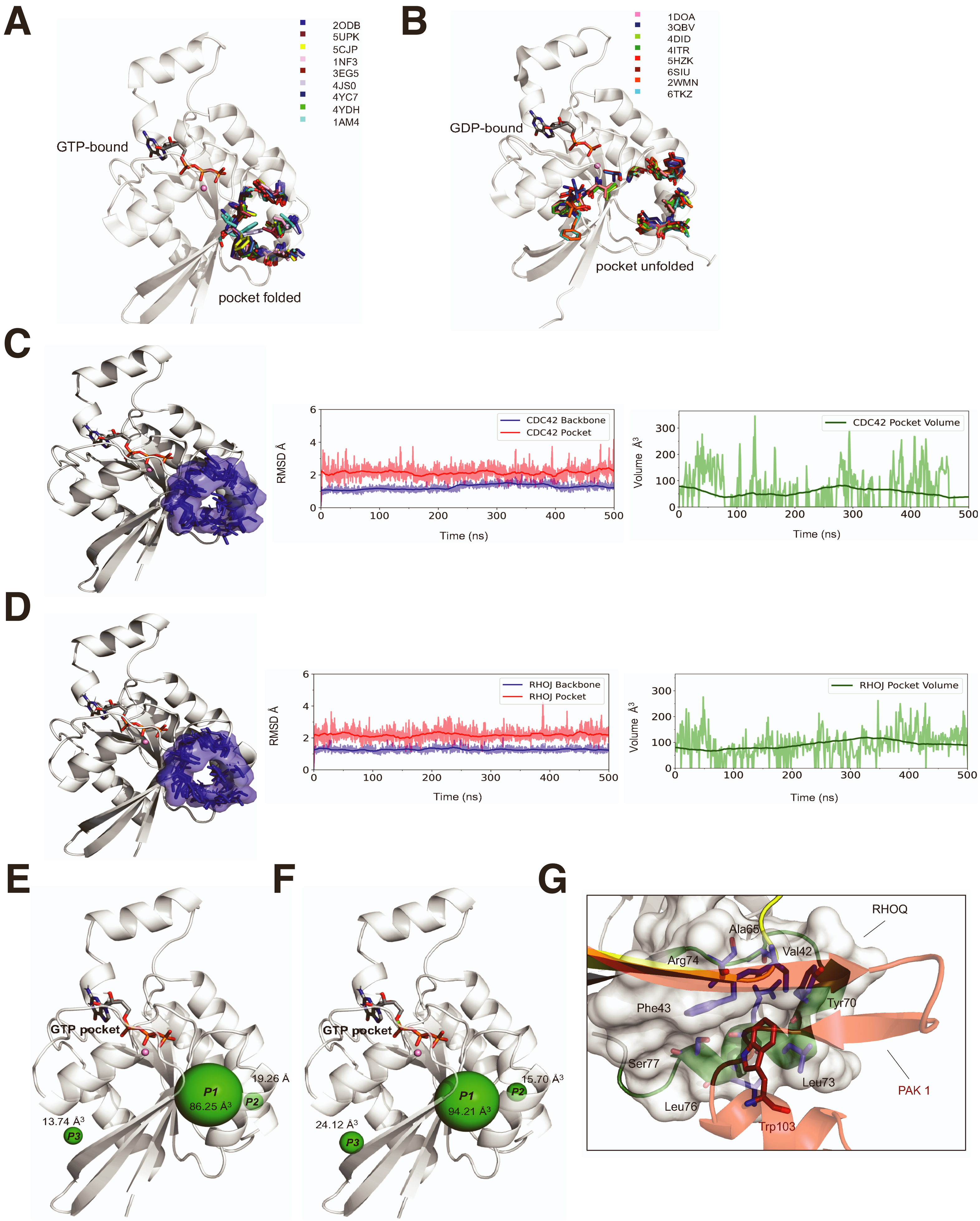
A
2ODB
5UPK
5CJP
1NF3
3EG5
4JS0
4YC7
4YDH
1AM4
GTP-bound
pocket folded
B
1DOA
3QBV
4DID
4ITR
5HZK
6SIU
2WMN
6TKZ
GDP-bound
pocket unfolded
C
CDC42 Backbone
CDC42 Pocket
RMSD Å
Time (ns)
CDC42 Pocket Volume
Volume Å3
Time (ns)
D
RHOJ Backbone
RHOJ Pocket
RMSD Å
Time (ns)
RHOJ Pocket Volume
Volume Å3
Time (ns)
E
GTP pocket
P1
86.25 Å3
P2
19.26 Å
P3
13.74 Å3
F
GTP pocket
P1
94.21 Å3
P2
15.70 Å3
P3
24.12 Å3
G
RHOQ
Ala65
Val42
Arg74
Phe43
Tyr70
Ser77
Leu73
Leu76
Trp103
PAK 1

Figure S1: Structural analysis of the CDC42 crystal structure (related to **Fig. 1A-B**; see also **Table S1**). Structures of GTP-bound CDC42 (**A**) and GDP-bound CDC42 (**B**) are reported. The protein is represented as cartoon, while the residues at the CDC42-effector interface are highlighted as sticks. Residue folds define the allosteric pocket only in the GTP-bound state (**A**). **MD simulations of GTP-bound CDC42 and RHOJ** (related to **Fig.1A-B**). The structural representation of both GTP-bound CDC42 (**C**) and RHOJ (**D**) is reported on the left. Both CDC42 and RHOJ are represented as cartoon, while the binding pocket is highlighted as blue transparent surface. Multiple MD snapshots of the pocket residues (blue) are shown as sticks. On the right is reported the Root-Mean Square Deviation (RMSD) of the pocket and protein backbone and the volume of the pocket for both CDC42 (**C**) and RHOJ (**D**). The running average is in bold in each graph. **Binding pocket on CDC42 and RHOJ at the protein-protein interface, analyzed from MD simulations** (related to **Fig.1D-E**). The structural representation of both GTP-bound CDC42 (**E**) and RHOJ (**F**) is reported, with the pocket represented in green spheres. The software pocketron was used to run the unsupervised analysis of our MD trajectories and identify such pockets at the protein-protein interface. The volume of such pockets is reported for both the proteins. **Close-view of the RHOQ-PAK1 interaction interface** (related to **Fig.1D-E**). **G)** The modeling of the interaction between Trp103 (red) of PAK1 (transparent red) and the effector binding pocket (light blue) of RHOQ (grey) is shown. Switch regions are highlighted in yellow and green cartoon.

Figure S2

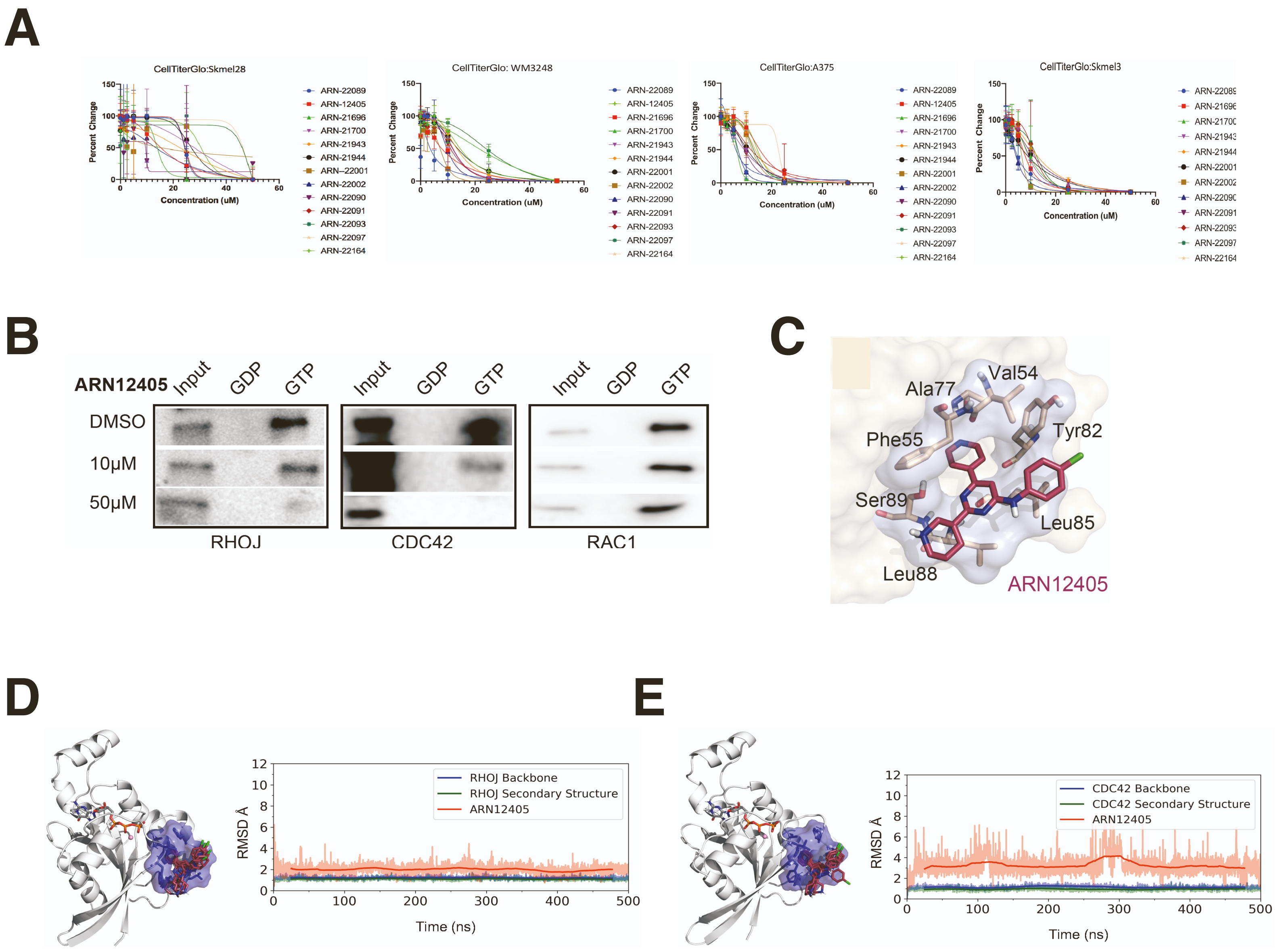

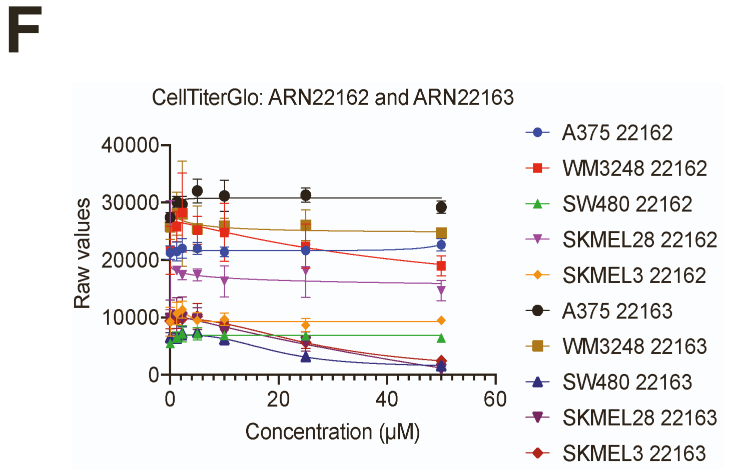

G

Tumor Type	Mutation	Cell Line	IC50 [M] ARN22089
Skin	BRAF	SK-MEL-3	3.3.0E-06
Skin	BRAF	A2058	3.70E-06
Skin	BRAF	A375	4.50E-06
Skin	BRAF	MDA MB 435	1.00E-05
Brain	KRAS and/or NRAS	SK-N-SH	8.10E-06
Stomach	KRAS and/or NRAS	SNU-1	7.80E-06
Bone	KRAS and/or NRAS	SJSA-1	9.50E-06
Colon	None	DLD-1	5.00E-06
Brain	None	SK-N-MC	4.70E-06

Figure S2: Characterization of putative CDC42/RHOJ interaction inhibitors in multiple cell lines and lead compound showing binding to effector interaction (related to **Fig.2C**). **(A)** Graphical representation of the IC50 data displayed in Figure 2C. Results are from 3 independent experiments (five replicates per data point) in each of five cell lines. **Lead compound shows inhibition of the effector interaction** (related to **Fig.2B-C**). **(B)** Model structure of ARN12405 bound to CDC42/RHOJ is also shown. **(C)** WM3248 cells were treated with the indicated doses of ARN12405 and the effect on RHOJ/CDC42 interaction was measured using a CDC42 interaction assay. **MD simulations of protein-ligand complexes** (related to **Fig.2B-C**). The structural representation of RHOJ (**D**) and CDC42 (**E**) in complex with ARN12405 is reported on the left. Both RHOJ and CDC42 are represented as cartoon, while the binding pocket is highlighted as blue transparent surface. On the right, the RMSD over time for both RHOJ (**D**) and CDC42 (**E**) binding complexes. The RMSD running averages is in bold. **Compounds that do not bind to the effector interaction domain** (related to **Fig.2C**). **(F)** Graphical representation of the IC50 data from two analogs that showed no response in five cell lines (3-5 replicates per data point, each cell line repeated at least twice). **Cells with non BRAF or RAS mutations are also responsive to the inhibitor** (related to **Fig.2D**). **(G)** Representation of cells with and without BRAF or RAS mutations and their IC50 values are shown (see Supplemental Table S4 for more details).

Figure S3

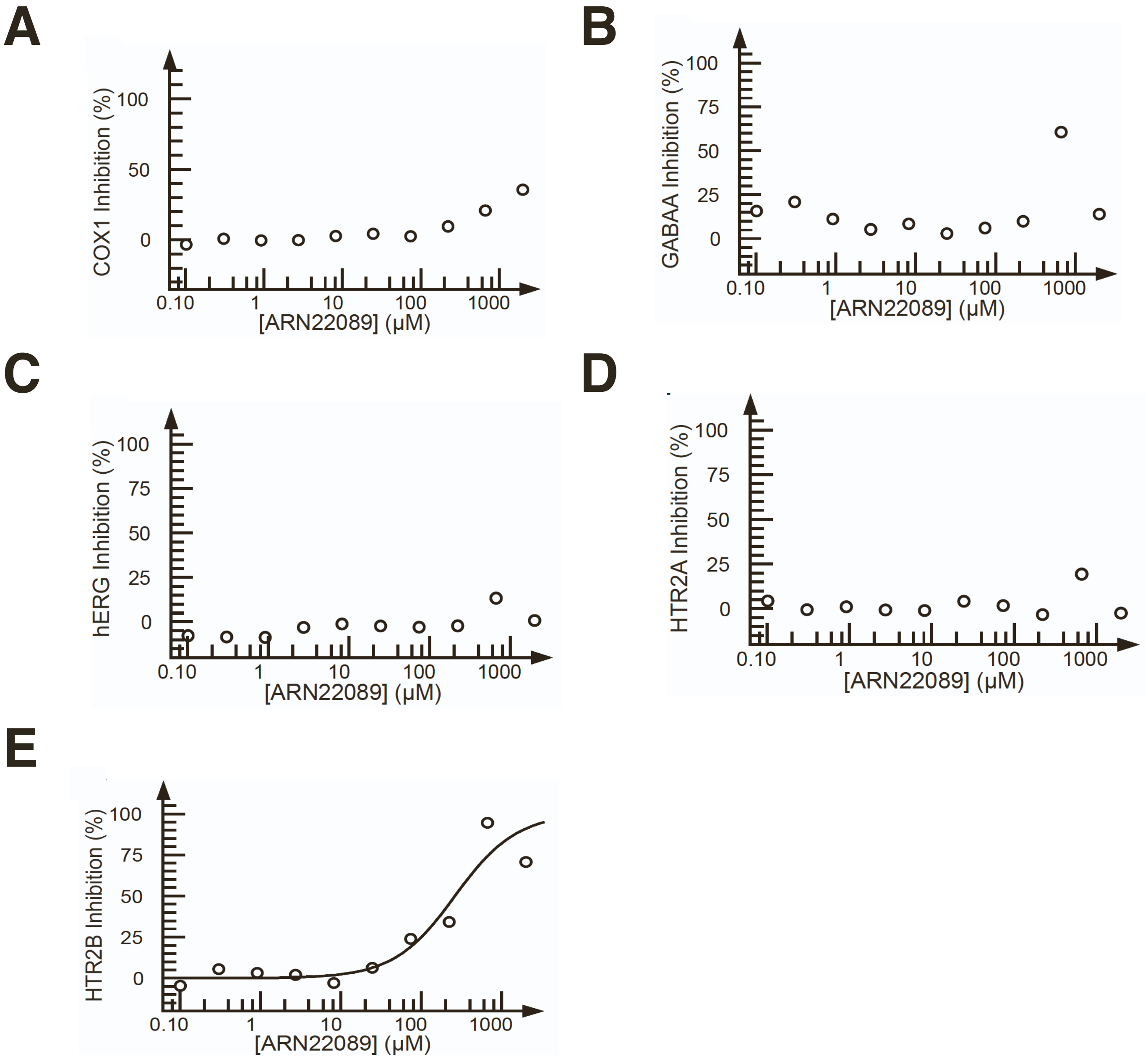
A
COX1 Inhibition (%)
100
50
0
0.10
1
10
100
1000
[ARN22089] (μM)
B
GABAA Inhibition (%)
100
75
50
25
0
0.10
1
10
100
1000
[ARN22089] (μM)
C
hERG Inhibition (%)
100
75
50
25
0
0.10
1
10
100
1000
[ARN22089] (μM)
D
HTR2A Inhibition (%)
100
75
50
25
0
0.10
1
10
100
1000
[ARN22089] (μM)
E
HTR2B Inhibition (%)
100
75
50
25
0
0.10
1
10
100
1000
[ARN22089] (μM)

Figure S3: Activity of ARN22089 measured against a panel of common off-target proteins (related to **Fig.3**). Curves for **(A)** COX1, **(B)** GABAA, **(C)** hERG, **(D)** HTR2A, **(E)** HTR2B are shown.

Figure S4

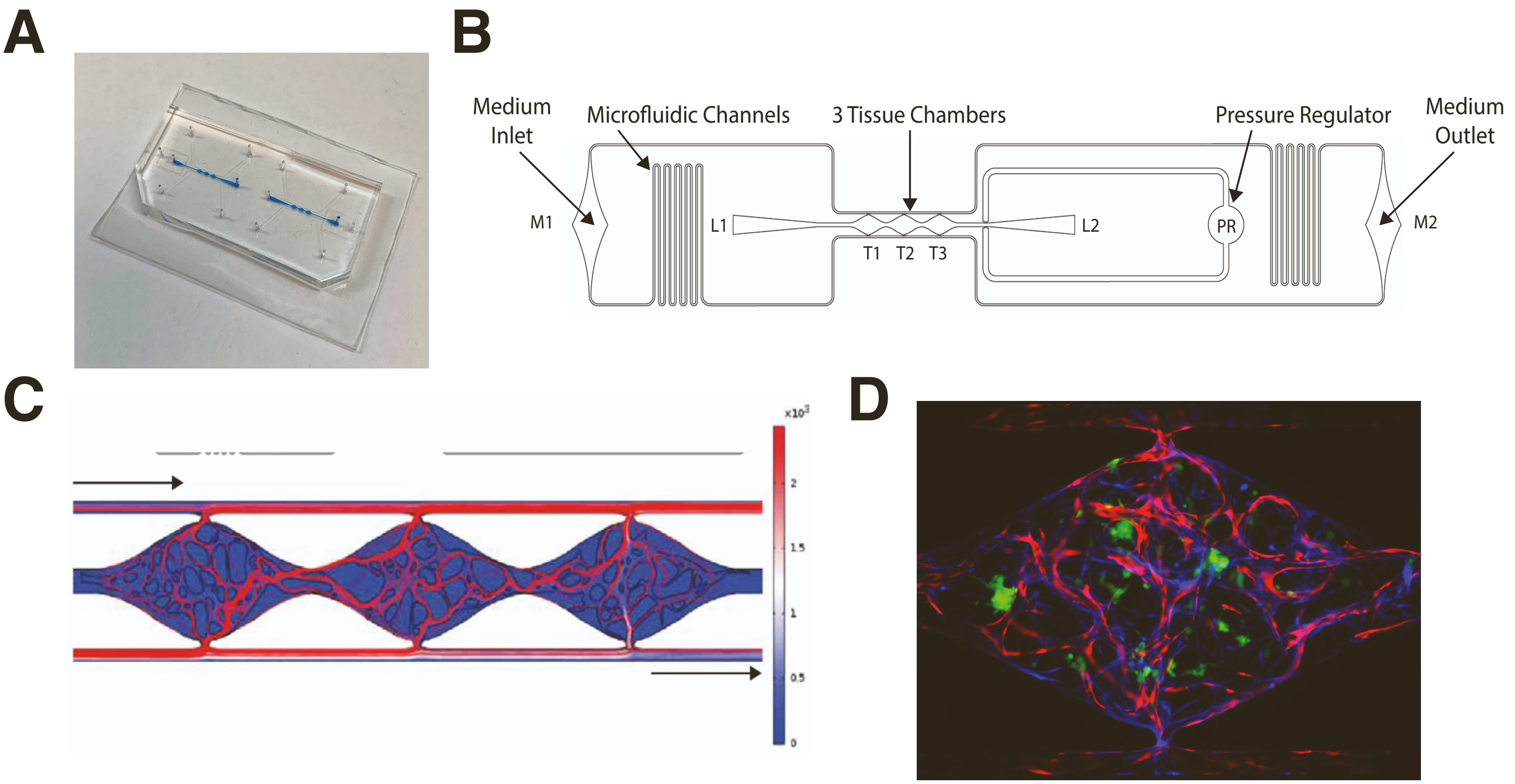
A
B
Medium
Inlet
Microfluidic Channels
3 Tissue Chambers
Pressure Regulator
Medium
Outlet
M1
L1
T1
T2
T3
L2
PR
M2
C
D

Figure S4: Vascularized microtumor (VMT) model (related to **Fig.4**). **(A)** Photograph of microfluidic platform with blue dye injected into tissue chambers. (**B**) Zoom view shows a single device unit with 3 tissue chambers (T1-3) fed through microfluidic channels, 2 loading ports (L1-2), medium inlet and outlet (M1-2), and pressure regulator (PR) to prevent gel bursting during loading. (**C**) Simulation of flow through a formed and perfused vascular network. Top left is high pressure and bottom right is low pressure. (**D**) Fluorescent micrograph of the VMT with vessels labeled red (mCherry), fibroblasts labeled blue (azurite), and cancer cells labeled green (GFP). Chamber is 2 mm x 1 mm x 0.1 mm.

Figure S5

A

Chk1_pS345.R.C	c.IAP2.R.C	FABP5.R.C	P.Cadherin.R.C
DAPK1_pS308.M.C	CD134.R.V	Gli3.R.C	PR.R.V
EGFR_pY1068.R.C	CD171.M.V	HSP27.M.C	PYGB.R.V
MAPK_pT202_Y204.R.C	CD2.M.C	Lck.R.V	Rad50.R.V
MDM2_pS166.R.V	CD29.M.V	MACC1.R.V	Smad3.R.V
p27_pT157.R.C	CD31.M.V	Merlin.R.C	Snail.M.Q
PRAS40_pT246.R.V	CD45.M.V	MIG6.R.V	Src.M.V
S6_pS235_S236.R.V	CD74.M.V	MMP14.R.V	WIPI1.R.C
S6_pS240_S244.R.V	eEF2K.R.V	MSH6.R.C	
SHP.2_pY542.R.C	EGFR.R.V	N.Ras.M.V	

B

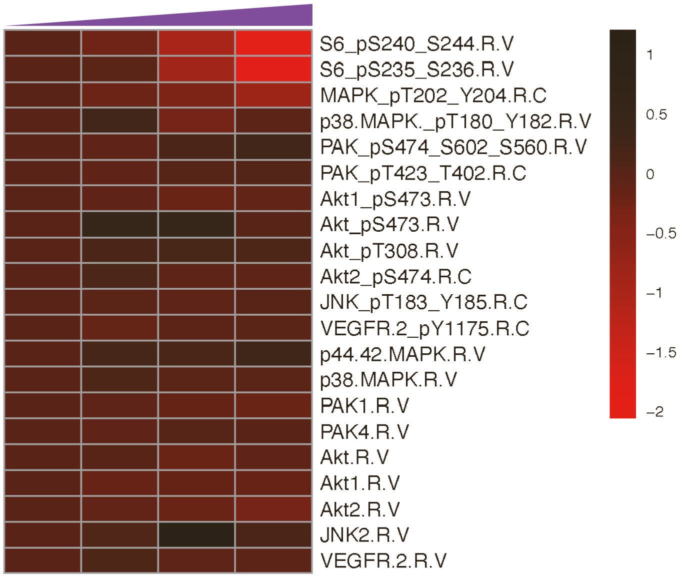

C

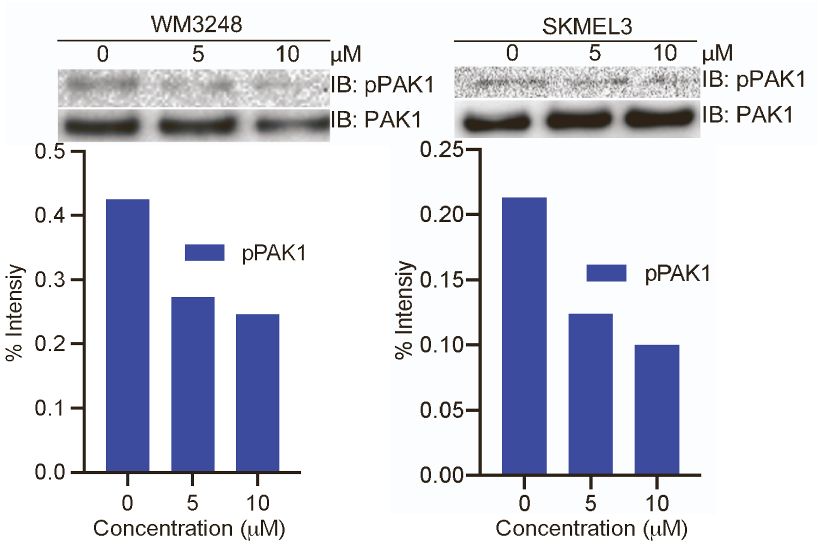

Figure S5: ARN22089 affects the phosphorylation of kinases and enzymes downstream of the CDC42 GTPases in melanoma (related to **Fig.3**). **(A)** Highlighted in orange are 10 phosphorylated proteins that are part of the 38 proteins that were found significantly changed in the RPPA (See Supplemental Table S7). **(B)** Heatmap shows the profile of some phosphorylated events influenced by the inhibitor. Purple triangle indicates increasing doses (0, 5, 10 and 20 μM) for 6 h. **(C)** Immunoblot for phosphorylated and total PAK1 from WM3248 and SKMEL3 that were treated with indicated doses of ARN22089 for 6 h. ImageJ was used to quantify the bands that are represented in the bar graphs.

Figure S6

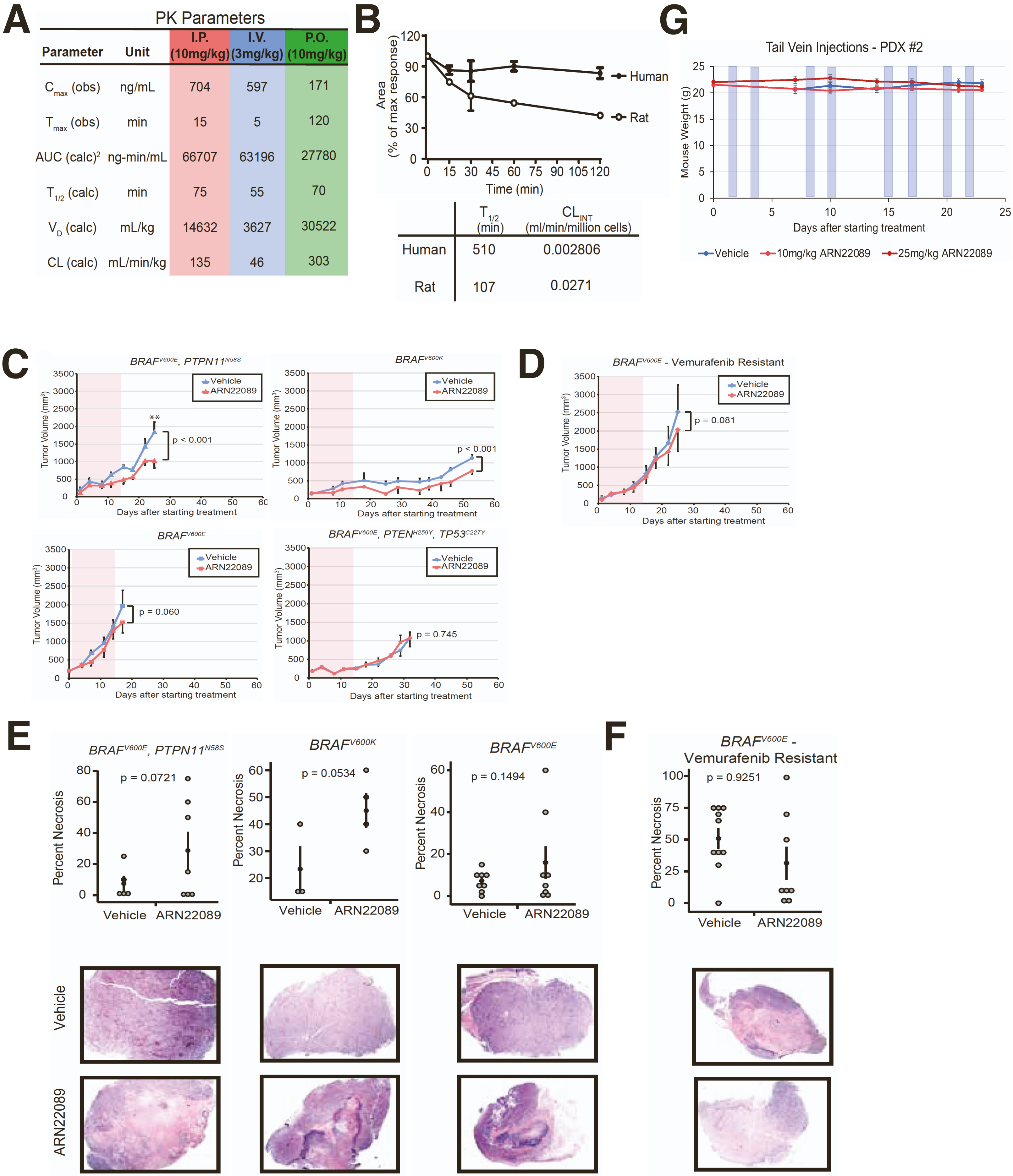

A
PK Parameters
Parameter | Unit | I.P. (10mg/kg) | I.V. (3mg/kg) | P.O. (10mg/kg)
Cmax (obs) | ng/mL | 704 | 597 | 171
Tmax (obs) | min | 15 | 5 | 120
AUC (calc)2 | ng-min/mL | 66707 | 63196 | 27780
T1/2 (calc) | min | 75 | 55 | 70
VD (calc) | mL/kg | 14632 | 3627 | 30522
CL (calc) | mL/min/kg | 135 | 46 | 303
B
Area (% of max response)
Time (min)
Human
Rat
T1/2 (min) | CLINT (ml/min/million cells)
Human | 510 | 0.002806
Rat | 107 | 0.0271
G
Tail Vein Injections - PDX #2
Mouse Weight (g)
Days after starting treatment
Vehicle
10mg/kg ARN22089
25mg/kg ARN22089
C
BRAFV600E, PTPN11N58S
BRAFV600K
BRAFV600E
BRAFV600E, PTENH259Y, TP53C227Y
Tumor Volume (mm3)
Days after starting treatment
Vehicle
ARN22089
**
p < 0.001
p < 0.001
p = 0.060
p = 0.745
D
BRAFV600E - Vemurafenib Resistant
p = 0.081
E
BRAFV600E, PTPN11N58S
BRAFV600K
BRAFV600E
Percent Necrosis
p = 0.0721
p = 0.0534
p = 0.1494
Vehicle
ARN22089
F
BRAFV600E - Vemurafenib Resistant
p = 0.9251

Figure S6: ARN22089 has drug like properties and inhibits tumor growth in vivo (related to **Fig.5**). PK values of ARN22089 delivered I.P., I.V., and P.O. at the indicated doses was determined and is shown **(A)**. **ARN22089 is stable in plasma** (related to **Fig.2C**). The stability of ARN22089 after incubation with human and rat liver microsomes was determined. Graph represents percent of maximum response as a function of time. Table below shows the half-life and intrinsic clearance for human and mouse **(B)**. **Individual tumor growth curves for ARN22089 carrying PDX tumors** (related to **Fig.5C**). Tumors with listed mutations treated with vehicle (blue) or 10 mg/kg ARN22089 (red) I.P. for 14 days. Treated days by I.P. are indicated by red shading. $BRAF^{V600E}$, $PTPN11^{N58S}$, n=6 tumors; $BRAF^{V600K}$, n=4 tumors; $BRAF^{V600E}$, n=8 tumors, $BRAF^{V600E}$, $PTEN^{H259Y}$, $TP53^{C227Y}$, n=6 vehicle and 8 ARN22089 treated tumors**.** Two-way ANOVA statistical tests compared treatment groups for each PDX over time with TukeyHSD pairwise comparisons between timepoints; * $p < 0.05$, ** $p < 0.01$, *** $p < 0.001$ (**C**). **BRAF inhibitor resistant tumors can respond to CDC42 inhibitors** (related to **Fig.5C-D**). $BRAF^{V600E}$ – Vemurafenib Resistant, n=10 tumors were treated with ARN22089 as described in (**C**) and shown (**D**), **ARN22089 induces tumor necrosis** (related to **Fig.5C-D**). Degree of necrosis present in hematoxylin and eosin (H&E) stained sections was determined by a blinded pathologist; n=5 vehicle and 7 ARN22089 treated for $BRAF^{V600E}$, $PTPN11^{N58S}$; n=3 vehicle and 4 ARN22089 treated for $BRAF^{V600k}$; n=7 vehicle and 8 ARN22089 treated $BRAF^{V600E}$; (shown in (**E**)) and n=10 vehicle and 8 ARN22089 treated for $BRAF^{V600E}$ – Vemurafenib Resistant (shown in (**F**) Necrosis scores for vehicle and ARN22089 treated tumors were compared using student's t-test; *$p < 0.05$. Representative images of H&E slides of vehicle and ARN22089 treated tumors shown below. **ARN22089 treatment does not induce cachexia**. Line graph represent mouse weights (g) during treatment, blue shaded boxes indicate I.V. treatments (related to **Fig.5D**) (shown in (**G**)).

Figure S7

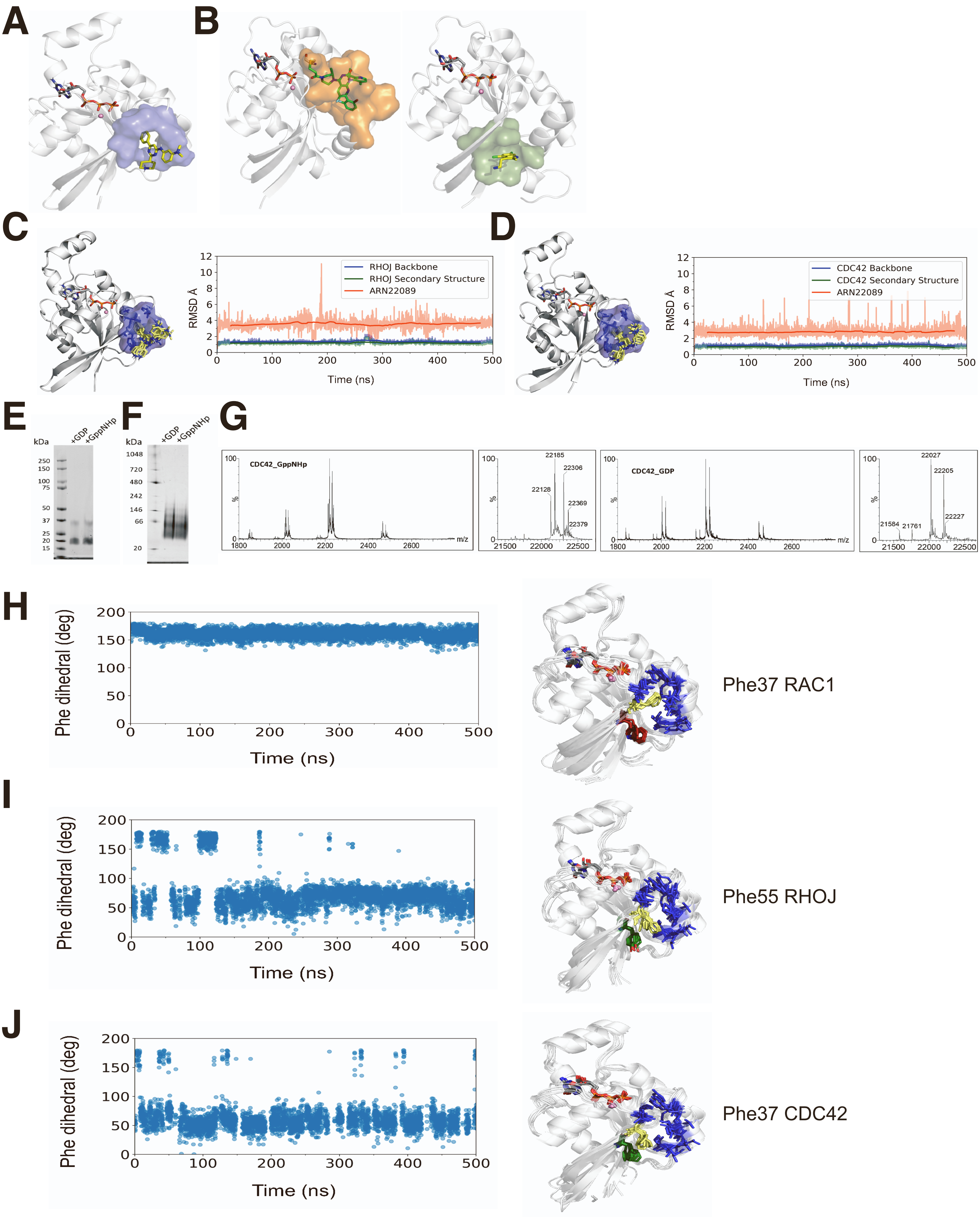
A
B
C
RMSD Å
Time (ns)
RHOJ Backbone
RHOJ Secondary Structure
ARN22089
D
CDC42 Backbone
CDC42 Secondary Structure
ARN22089
E
F
kDa
+GDP
+GppNHp
G
CDC42_GppNHp
CDC42_GDP
m/z
%
22185
22306
22128
22369
22379
22027
22205
22227
21584
21761
H
Phe dihedral (deg)
Phe37 RAC1
I
Phe55 RHOJ
J
Phe37 CDC42

Figure S7: Hit compounds inhibit RHOJ/CDC42 effector interactions. Structural analysis and comparison of the pocket of the CDC42 GTPases compared to K-RAS (related to **Fig.6A-B**). **(A)** Structural representation of lead compound ARN22089 (yellow sticks) docked into the binding site (blue surface) of CDC42. **(B)** *Left*, The K-RAS(G12C) covalent inhibitors AMG-510 (green sticks, PDB: 6OIM) at the inhibitor's pocket (orange surface). *Right*, The K-RAS-selective inhibitor DCAI bound to the pocket (green surface, PDB: 4DST). Notably, in **(B)**, the pocket of **(A)** is missing. **MD simulations of protein-ligand complexes** (related to **Fig.6A-B**). The structural representation of RHOJ (**C**) and CDC42 (**D**) in complex with ARN22089 is reported on the left. Both RHOJ and CDC42 are represented as cartoon, while the binding pocket is highlighted as blue transparent surface. Multiple MD snapshots of the ARN22089 (yellow) binding poses are shown as sticks. On the right, the RMSD over time for both RHOJ (**C**) and CDC42 (**D**) binding complexes. The RMSD running averages is in bold. **Quality control of His-CDC42 protein production and nucleotide loading** (related to **Fig.6C**). GDP or GppNHp-loaded samples were compared of a His-CDC42 fragment (residues 4-182) using: **E)** SDS-PAGE 4-12 % v/v acrylamide; **F)** Native gel 4-16 % v/v acrylamide. **G)** ESI MS+ spectra and deconvoluted molecular weight of nucleotide bound His-CDC42. >98% of His-Cdc42 is estimated to be loaded with GppNHp and around 90% with GDP (with the remaining as apo-protein). Molecular weights + / - α-N-gluconoylation (+ 178 Da)[61]: 21584 / 21761 Da – apo-protein; 22027/ 22205 Da – His-Cdc42 + GDP; 22128 / 22306 Da – His-Cdc42 + GppNHp + Mg^{2+}; 22185 / 22369 Da – His-Cdc42 + GppNHp + Mg^{2+} + Ni^{2}. **MD simulations of GTP-bound proteins** (related to **Fig.6A-B and 1F-H**). We looked for the allosteric pocket on the surface of RAC1 by comparing the X-ray structures of RAC1 and CDC42, and our RHOJ homology model. Surprisingly, this structural analysis revealed that one residue that defines the allosteric pocket, Phe37, is captured in a "closed" conformation only in RAC1. Specifically, Phe37 (CDC42) and Phe55 (RHOJ) adopt always an open conformation, which allows binding to the allosteric pocket. Instead, in RAC1, the Phe37 is found in either open or closed conformations, with the latter that occludes the binding pocket. Notably, the Phe37 is stably maintained in its closed conformation during 500ns of MD simulations of RAC1. This is likely due to the presence of the bulky Trp56, which is next to Phe37. Trp56 corresponds to less bulky residues in CDC42 and RHOJ (specifically Phe56 and Tyr74, respectively), which may allow for more conformational flexibility of Phe37. The value and time evolution of the side chain dihedral angle of Phe37 in RAC1 (**H**) vs Phe55 in RHOJ (**I**) and Phe37 in CDC42 (**J**) highlights its different orientation and conformational flexibility. Proteins are represented as cartoon, while guanine nucleotides and Mg^{2+} ions are illustrated as sticks and balls, respectively. The residues defining the binding pocket are are shown as blue sticks, and the Phe37 (Phe55 in RHOJ) is highlighted in yellow. The Trp56 residue in Rac1 is shown as red sticks while the corresponding Phe56 (CDCD42) and Tyr74 (RHOJ) are reported in green. In RAC1, the presence of Trp56 favors the stabilization of Phe37 in a closed conformation, thus hampering the accessibility of the drug-binding pocket.

Figure S8

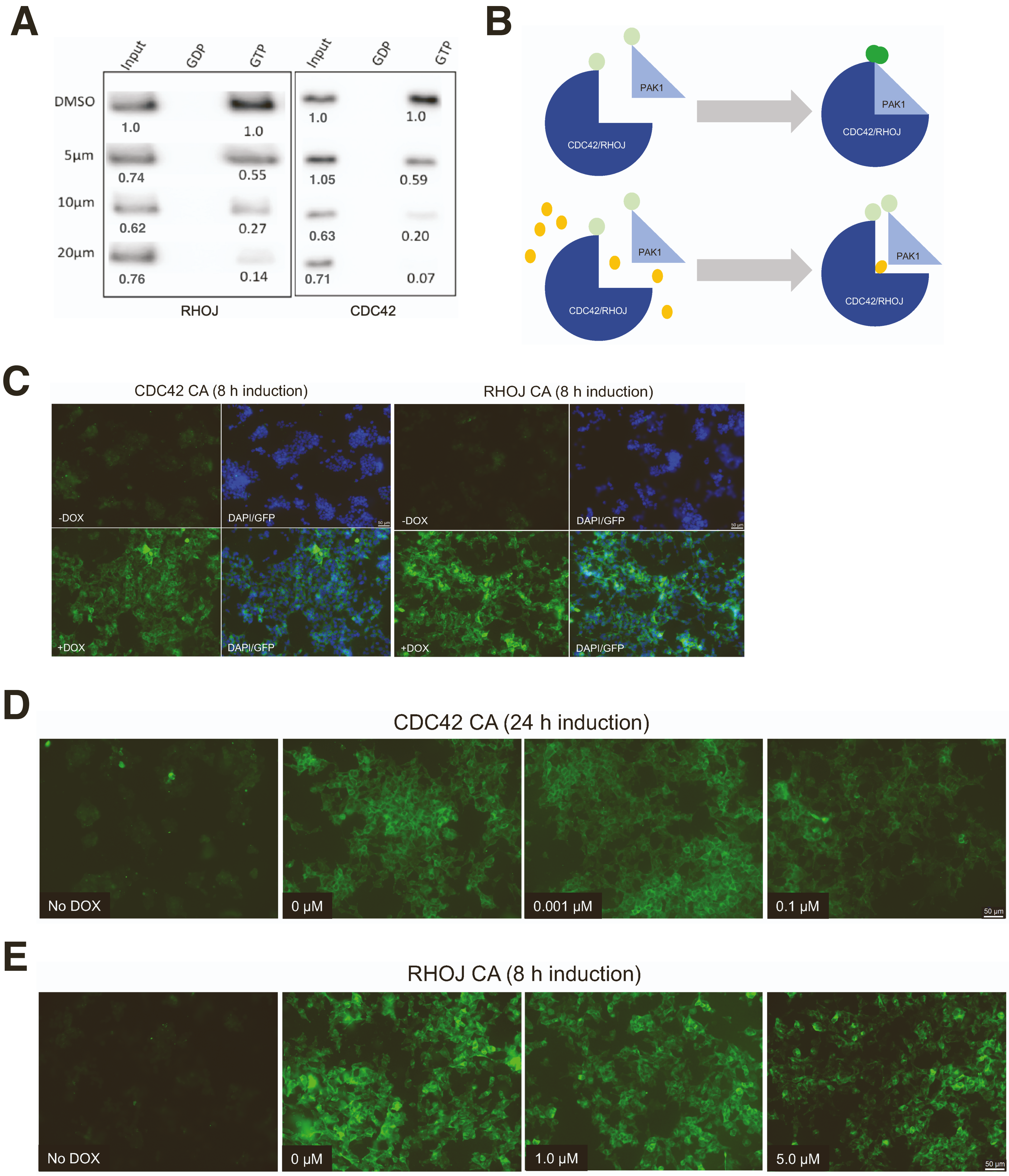
A
Input
GDP
GTP
Input
GDP
GTP
DMSO
1.0
1.0
1.0
1.0
5µm
0.74
0.55
1.05
0.59
10µm
0.62
0.27
0.63
0.20
20µm
0.76
0.14
0.71
0.07
RHOJ
CDC42
B
PAK1
CDC42/RHOJ
PAK1
CDC42/RHOJ
PAK1
CDC42/RHOJ
PAK1
CDC42/RHOJ
C
CDC42 CA (8 h induction)
RHOJ CA (8 h induction)
-DOX
DAPI/GFP
-DOX
DAPI/GFP
+DOX
DAPI/GFP
+DOX
DAPI/GFP
D
CDC42 CA (24 h induction)
No DOX
0 µM
0.001 µM
0.1 µM
50 µm
E
RHOJ CA (8 h induction)
No DOX
0 µM
1.0 µM
5.0 µM
50 µm

Figure S8: CDC42 pulldown assay of unloaded and loaded guanine nucleotides (related to **Fig.6C**). **(A)** Western Blot of Cdc42 activation assay. ImageJ was used to quantify the bands of input and GTP for each condition. Background was subtracted and each dose was normalized to DMSO. **Schematic diagram of the BiFC assay** (related to **Fig.6D-E**). **(B)** In the absence of the lead drug, RHOJ/CDC42 and PAK1 bind and the cell exhibits YFP fluorescence. In the presence of the drug, RHOJ/CDC42 and PAK1 interaction is inhibited and no fluorescence is observed. **Development of a BiFC interaction assay to measure the cell activity of CDC42 interaction inhibitors** (related to **Fig.6D-E**)**. (C)** Images of CDC42 constitutive active (CA) and RHOJ constitutive active (CA) BiFC cells. Cells were treated with or without DOX (2 µg/mL) for 8 h to induce protein expression. **CDC42 interaction inhibitors block CDC42 PAK interactions** (related to **Fig.6D-E**)**.** Representative images of BiFC cells induced (2 µg/mL DOX) and treated with indicated doses for **(D)** CDC42 CA at 0, 0.001, and 0.1 µM for 24 h and **(E)** RHOJ CA at 0, 1, 5 µM for 8 h.

Table S1: CDC42 X-ray structures used for the structural analysis and pocket identification. The 18 CDC42 structures used in the analyses are listed (see **Fig.1** and **Fig.S1A-B**).

PDB ID	*Resolution (Å)*	*State*	*Substrate*	*Pocket Folded*
2ODB	2.4	Active	GCP	YES
5UPK	2.4	Active	GNP	YES
5CJP	2.6	Active	GTP	YES
1NF3	2.1	Active	GNP	YES
3EG5	2.7	Active	GNP	YES
4JS0	1.9	Active	GNP	YES
4YC7	2.5	Active	GNP	YES
4YDH	3.8	Active	GNP	YES
1AM4	2.7	Active	GNP	YES
1E0A	NMR-Structure	Active	GNP	YES
1DOA	2.6	Inactive	GDP	NO
3QBV	2.65	Inactive	GDP	NO
4DID	2.35	Inactive	GDP	NO
4ITR	2.3	Inactive	GDP	NO
5HZK	3.3	Inactive	GDP	NO
6SIU	2.49	Inactive	GDP	NO
2WMN	2.391	Inactive	GDP	NO
6TKZ	2.64	Inactive	GDP	NO

Table_S2: Compounds tested with a dose response detected in the range 1.2 µM - 50 µM.
List of compounds tested in SkMeL28 with IC50s less than 50 µM (see **Fig.2**)

Inhibitor	Chemical Structure	Single Agent IC50
ARN12405	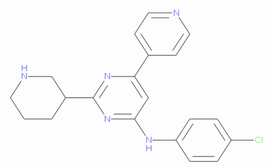	16.37
ARN21696	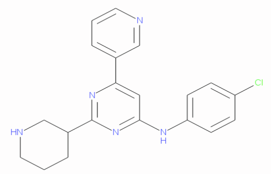	14.93
ARN21697	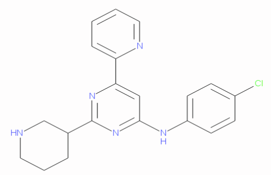	28.97
ARN21698	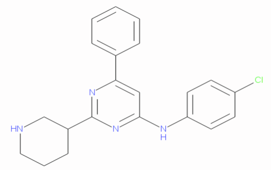	10.21
ARN21700	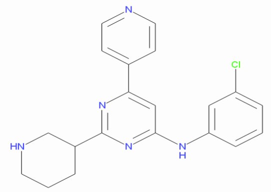	38.31
ARN21943	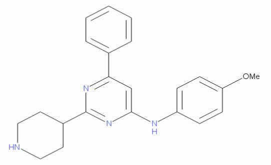	32.54

ARN21944	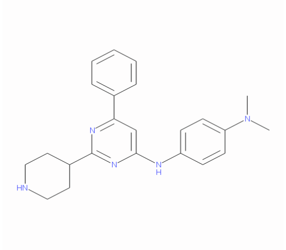	26.95
ARN22001	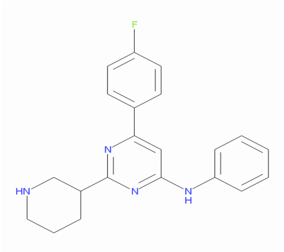	40.11
ARN22002	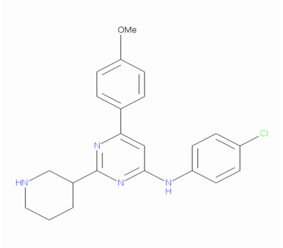	19.72
ARN22089	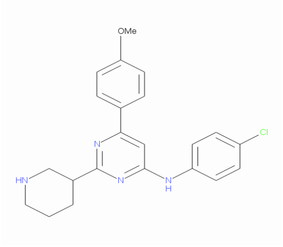	24.8
ARN22090	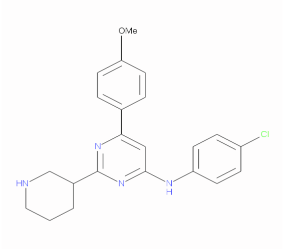	38.12
ARN22091	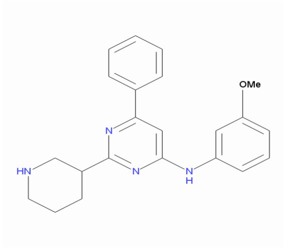	24.2

ARN22093	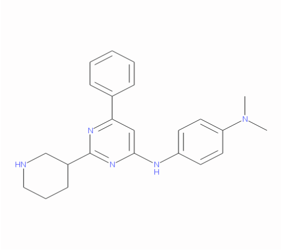	45.02
ARN22164	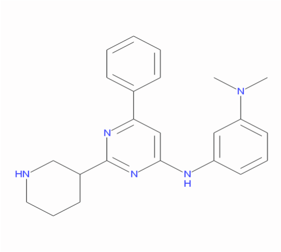	31.44

Table S3: Compounds tested without a dose response detected in the range 1.2 µM - 50 µM.
List of compounds with their chemical structure with IC50 above 50 µM in SKMel28 cells (see **Fig.2**)

Compound	Chemical Structure	Compound	Chemical Structure
ARN1690	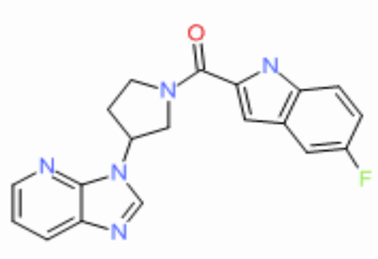	ARN8101	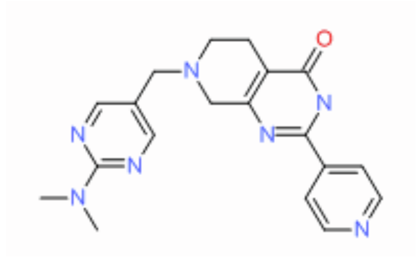
ARN1744	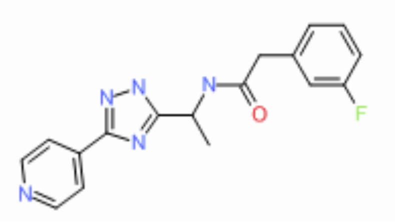	ARN8102	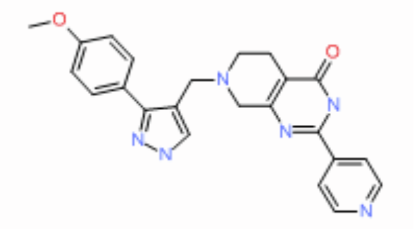
ARN1750	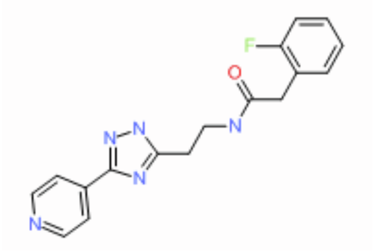	ARN8167	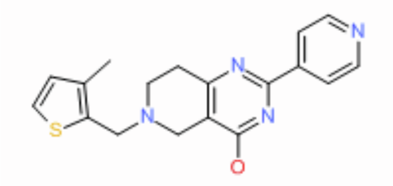
ARN1878	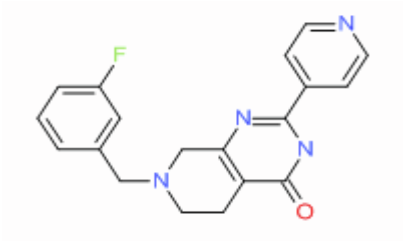	ARN8522	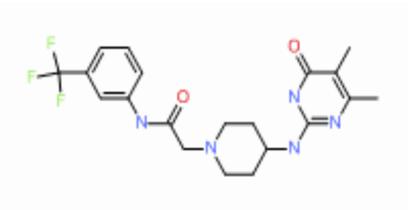
ARN2039	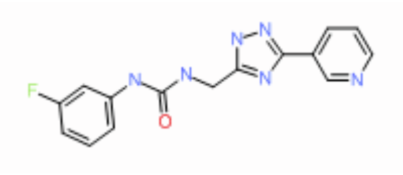	ARN8607	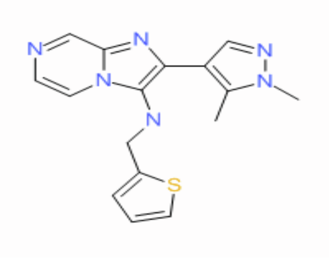

ARN2053	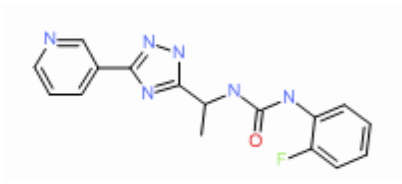	ARN8611	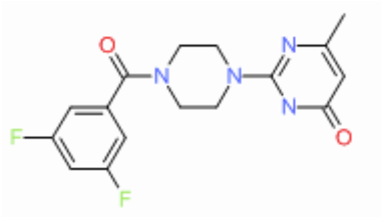
ARN2630	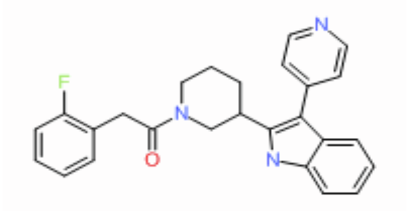	ARN8670	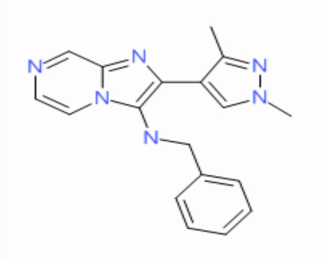
ARN2685	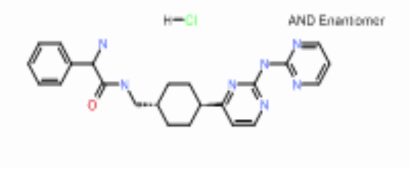	ARN8773	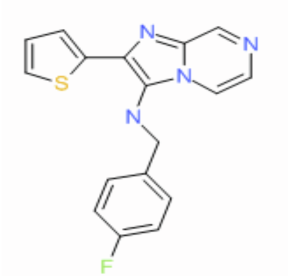
ARN3450	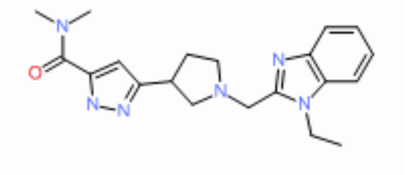	ARN9925	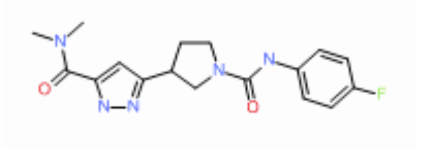
ARN3818	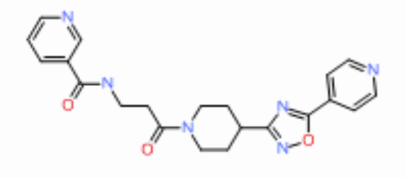	ARN10185	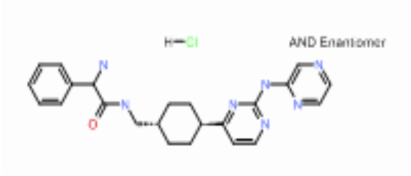
ARN3868	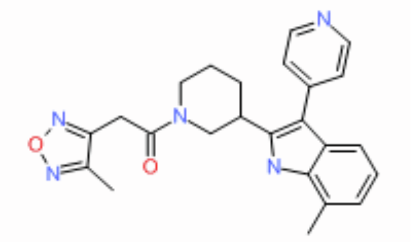	ARN10187	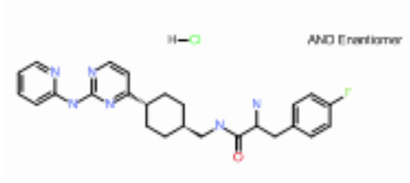

ARN3869	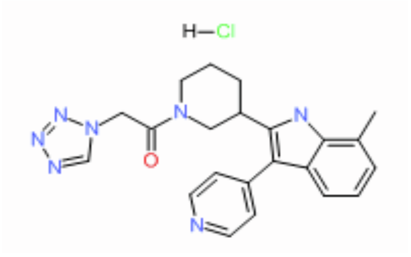	ARN10220	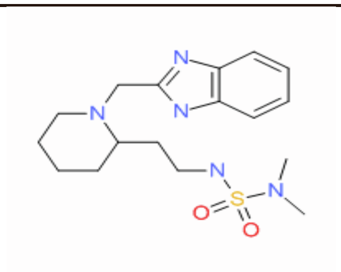
ARN4306	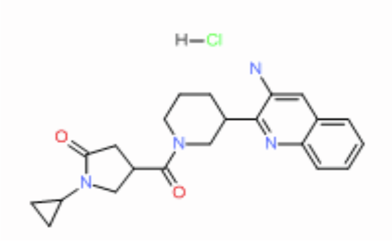	ARN10386	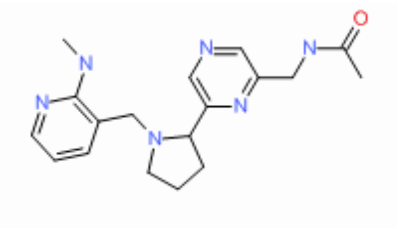
ARN4935	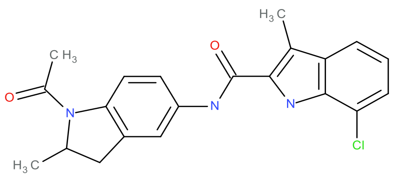	ARN10458	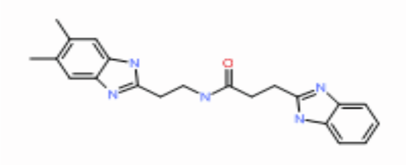
ARN4866	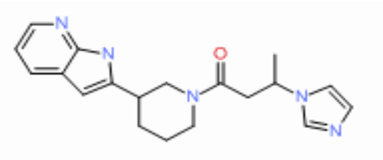	ARN10489	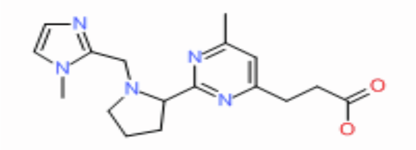
ARN5450	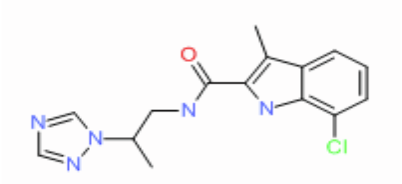	ARN10698	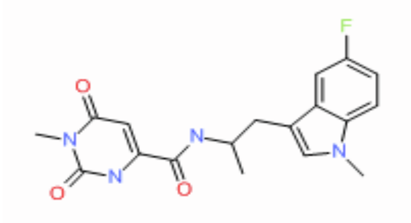
ARN5564	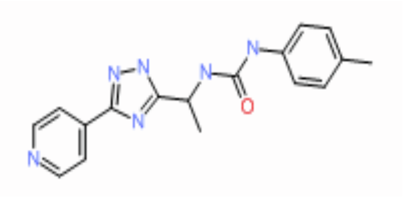	ARN10700	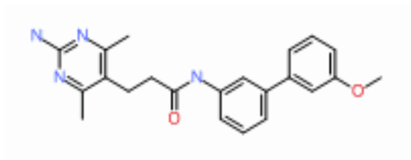

ARN5632	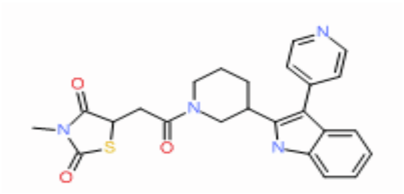	ARN11206	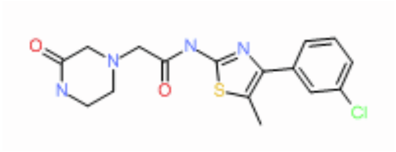
ARN5694	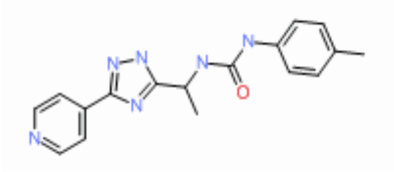	ARN11314	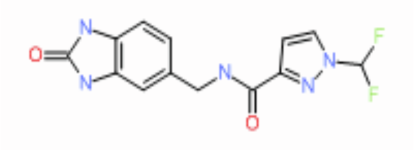
ARN5695	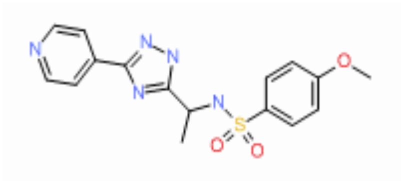	ARN11556	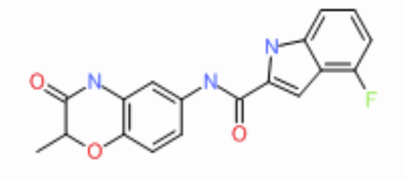
ARN5701	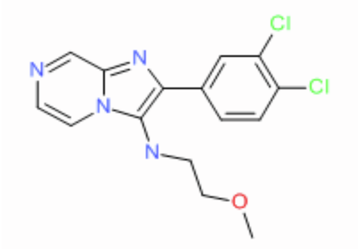	ARN12000	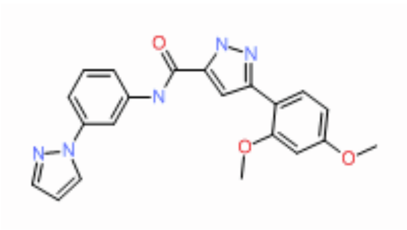
ARN5762	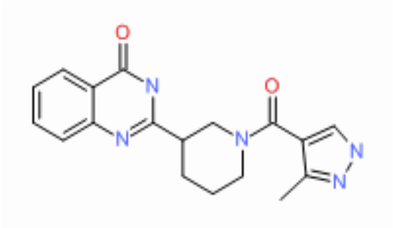	ARN12454	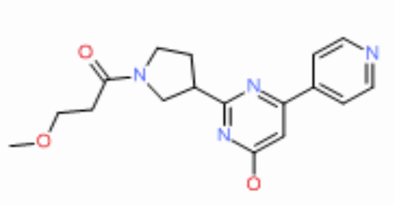
ARN5921	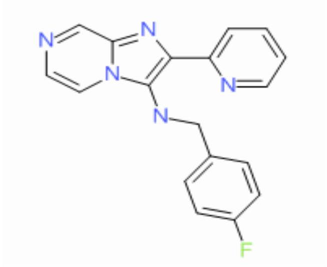	ARN12885	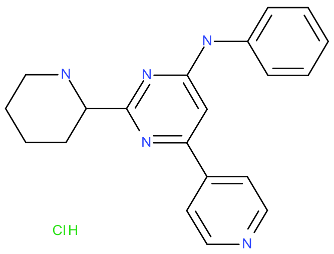

ARN6231	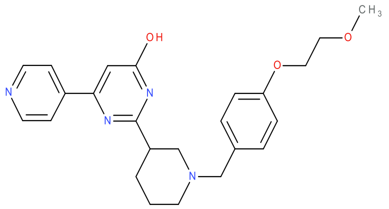	ARN12927	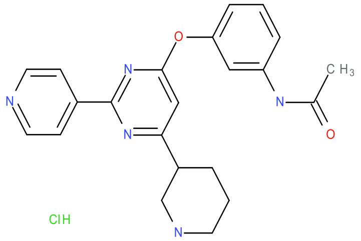
ARN6329	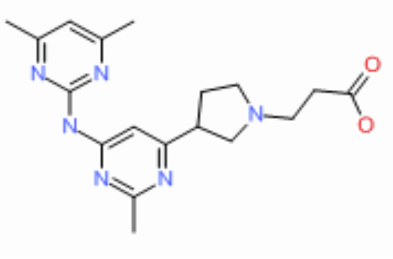	ARN8086	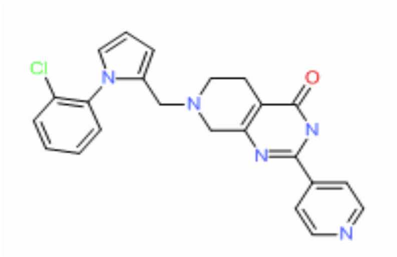
ARN6368	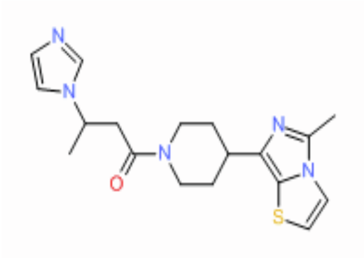	ARN8097	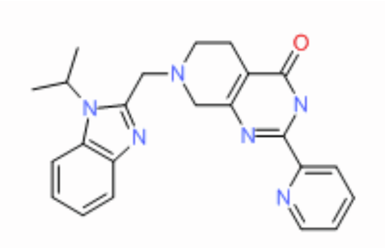
ARN6409	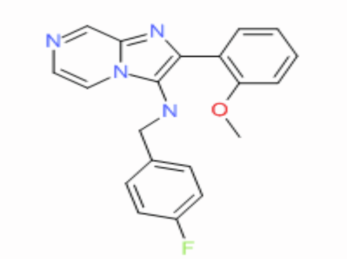	ARN7675	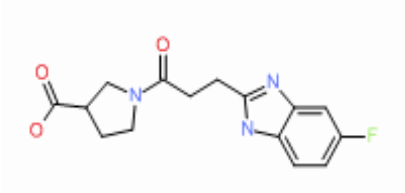

Table S4: ARN22089 IC50 in Cancer Cell Line panel: 100 cancer cell lines derived from different tumor types with various mutation status are listed in the table. These cells were treated with ARN22089 and their IC50 are listed in the table (related to **Fig.2D**).

Tumor Type	Cell line	IC50 [M] ARN22089	BRAF	TP53	KRAS	NRAS	PIK3C	PTEN
			Mutations Present					
Skin	SK-MEL-3	3.30E-06	x	x				
Colon	SW 480	3.40E-06		x	x			
Blood	U-937	3.50E-06		x				
Colon	HT-29	3.50E-06	x	x				
Liver	Hep3B2.1-7	3.60E-06						
Skin	A2058	3.70E-06	x	x				
Colon	RKO	3.90E-06	x				x	
Stomach	MKN-45	4.10E-06		x				
Blood	MV4-11	4.40E-06						
Skin	A375	4.50E-06	x					
Ovary	MCAS	4.60E-06			x		x	
Brain	SK-N-MC	4.70E-06						
Colon	DLD-1	5.00E-06						
Colon	HCT116	5.20E-06			x		x	
Colon	T84	5.30E-06			x		x	
Lung	SCLC-21H	5.40E-06		x				
Ovary	COV434	5.50E-06						
Stomach	NCI-N87	5.70E-06		x				
Ovary	RL95-2	5.70E-06						x
Blood	RPMI 8226	5.80E-06		x	x			
Brain	A172	5.80E-06						
Colon	SW 620	5.90E-06		x	x			
Bladder	J82	5.90E-06		x				
Brain	U87MG	6.10E-06						x
Ovary	SNU840	6.50E-06					x	
Breast	SK.BR-3	6.70E-06		x				
Colon	Colo 205	7.00E-06		x				
Breast	MCF-7	7.30E-06					x	
Ovary	OVK18	7.50E-06		x				
Ovary	SK-OV3	7.60E-06		x			x	
Lung	SK-LU-1	7.80E-06		x	x			
Colon	LOVO	7.80E-06			x			
Stomach	SNU-1	7.80E-06			x			
Brain	LN229	7.80E-06						
Blood	HL-60	8.10E-06		x		x		
Brain	SK-N-SH	8.10E-06				x		
Pancreas	Mia PaCA 2	8.30E-06		x	x			
Colon	SW 948	8.30E-06			x		x	

Lung	H460	8.50E-06			x		x	
Endometrium	Ishikawa	8.50E-06						x
Brain	H4	8.60E-06						x
Colon	HCT-15	8.70E-06		x	x		x	
Brain	U118MG	8.80E-06		x				x
Blood	K562	9.00E-06						
Bone	Saos-2 EC	9.00E-06						
Prostate	PC3	9.10E-06						
Lung	RERF-LC-Ad2	9.20E-06		x	x			
Endometrium	HEC-1-A	9.50E-06		x	x			
Bone	SJSA-1	9.50E-06		x	x	x		
Kidney	Caki-2	9.90E-06						
Pancreas	AsPC-1	9.90E-06		x	x			
Breast	HCC38	1.00E-05		x				
Breast	MDA MB 231	1.00E-05		x	x			
Skin	MDA MB 435	1.00E-05	x	x				
Kidney	A498	1.00E-05						
Pancreas	PANC-1	1.10E-05		x	x			
Lung	HCC827	1.10E-05		x				
Prostate	DU-145	1.10E-05		x				
Brain	SK-N-FI	1.10E-05		x				
Blood	KARPAS 299	1.10E-05		x				
Breast	HCC 1569	1.10E-05		x				x
Blood	MOLT-4	1.10E-05		x		x		x
Lung	A549	1.10E-05			x			
Blood	M07e	1.10E-05				x		
Kidney	Caki-1	1.10E-05						
Bone	U2OS	1.10E-05						
Lung	H1299	1.10E-05				x		
Fibrosarcoma	HT-1080	1.10E-05				x		
Lung	NCI-H838	1.20E-05						
Lung	BEN	1.20E-05		x				
Lung	NCI-H441	1.20E-05		x	x			
Ovary	EFO-27	1.20E-05		x				
Stomach	MKN-1	1.20E-05		x			x	
Lung	COR-L279	1.20E-05		x				
Lung	RERF-LC-MS	1.20E-05		x				
Bone	SK-ES-1	1.20E-05		x				
Lung	NCI-H292	1.20E-05						
Duodenum	Hutu 80	1.20E-05						
Ovary	OV56	1.30E-05		x	x			
Blood	MOLM-13	1.30E-05						
Lung	NCI-H2286	1.40E-05						

Breast	EFM-192A	1.40E-05		x			x	
Blood	KG-1	1.40E-05		x				
Blood	P31/FUJ	1.40E-05		x		x		x
Prostate	LnCap	1.40E-05		x				
Lung	NCI-H2009	1.50E-05		x	x			
Blood	OCI-AML5	1.50E-05						
Lung	EPLC-272H	1.60E-05						
Lung	NCI-H1437	1.60E-05		x				
Lung	NCI-H1563	1.60E-05					x	x
Lung	NCI-H1838	1.70E-05		x				
Endometrium	HEC-1-B	1.80E-05		x	x			
Lung	NCI-H2110	2.10E-05		x				
Lung	NCI-H1048	2.20E-05		x			x	
Lung	NCI-H1573	2.40E-05		x	x	x		
Lung	NCI-H1581	2.40E-05		x				
Lung	LOU-NH91	2.90E-05		x			x	
Breast	BT-20	2.90E-05		x			x	
Lung	DV90	2.90E-05			x			
Lung	NCI-H1703	3.70E-05		x				
